# eMCI: An Explainable Multimodal Correlation Integration Model for Unveiling Spatial Transcriptomics and Intercellular Signaling

**DOI:** 10.34133/research.0522

**Published:** 2024-11-01

**Authors:** Renhao Hong, Yuyan Tong, Hui Tang, Tao Zeng, Rui Liu

**Affiliations:** ^1^ School of Mathematics, South China University of Technology, Guangzhou 510640, China.; ^2^ School of Mathematics and Big Data, Foshan University, Foshan 528000, China.; ^3^ Guangzhou National Laboratory, Guangzhou, China.; ^4^ GMU-GIBH Joint School of Life Sciences, The Guangdong-Hong Kong-Macau Joint Laboratory for Cell Fate Regulation and Diseases, Guangzhou Laboratory, Guangzhou Medical University, Guangzhou, China.

## Abstract

Current integration methods for single-cell RNA sequencing (scRNA-seq) data and spatial transcriptomics (ST) data are typically designed for specific tasks, such as deconvolution of cell types or spatial distribution prediction of RNA transcripts. These methods usually only offer a partial analysis of ST data, neglecting the complex relationship between spatial expression patterns underlying cell-type specificity and intercellular cross-talk. Here, we present eMCI, an explainable multimodal correlation integration model based on deep neural network framework. eMCI leverages the fusion of scRNA-seq and ST data using different spot–cell correlations to integrate multiple synthetic analysis tasks of ST data at cellular level. First, eMCI can achieve better or comparable accuracy in cell-type classification and deconvolution according to wide evaluations and comparisons with state-of-the-art methods on both simulated and real ST datasets. Second, eMCI can identify key components across spatial domains responsible for different cell types and elucidate the spatial expression patterns underlying cell-type specificity and intercellular communication, by employing an attribution algorithm to dissect the visual input. Especially, eMCI has been applied to 3 cross-species datasets, including zebrafish melanomas, soybean nodule maturation, and human embryonic lung, which accurately and efficiently estimate per-spot cell composition and infer proximal and distal cellular interactions within the spatial and temporal context. In summary, eMCI serves as an integrative analytical framework to better resolve the spatial transcriptome based on existing single-cell datasets and elucidate proximal and distal intercellular signal transduction mechanisms over spatial domains without requirement of biological prior reference. This approach is expected to facilitate the discovery of spatial expression patterns of potential biomolecules with cell type and cell–cell communication specificity.

## Introduction

Single-cell technologies enable the simultaneous assessment of gene expression in thousands of cells [1,2]. The traditional single-cell RNA sequencing (scRNA-seq) requires tissue dissociation to the single-cell or single-nuclei level, resulting in the loss of spatial information and preventing direct linkage of the molecular features of the analyzed cell types to their anatomical and functional features [3]. By contrast, spatial transcriptomics (ST) methods bypass tissue dissociation, so that they can retain spatial information and enable gene expression analysis across thousands of cells within the context of tissue structural organization [1,4]. These ST technologies facilitate the exploration of the interactions among neighboring cells as well as intracellular and extracellular states, thereby improving our understanding of cellular functions across various species, organs, and tissues [5]. However, limited by spatial resolution, the gene expressions measured at each spatial spot are often from a mixture of several cells, where such mixture can obscure genuine transcriptional patterns and result in a biological misunderstanding of tissue structure, leading to distorted reconstructions of cell distributions and inter- and intramolecular cross-talk at the cellular level [5,6].

Recently, there has been a rise in integration methods that combine ST data and scRNA-seq data to simultaneously explore cell populations and their spatial locations within tissue [7]. In this way, they unveil biological insights that would otherwise not be accessible through a single experimental approach. For example, Cell2location uses the gene expression signature of the cell subpopulations in scRNA-seq data to estimate the abundance of each cell type at each spot [8]; SPOTlight applies the seeded non-negative matrix factorization for the deconvolution of spots [9]; Seurat applies canonical correlation analysis [10] to embed ST and scRNA-seq data into a common latent space, projecting cells from scRNA-seq data onto spots from ST data [11]; DestVI adopts the variational inference and latent variable models to delineate cell-type proportions [12]; STRIDE uses the topic profiles trained from scRNA-seq data to decompose cell types from spatial mixtures [3]; and SpaOTsc [13] and novoSpaRc [14] each use optimal transport methods [15] to construct spatial metrics of cells based on scRNA-seq data. Despite the availability of these existing tools, they are not straightforward for exploring potential molecular features and their phenotype associations in histological sections by integrating scRNA-seq and ST data. Each tool exhibits specific applicability, with varying performance observed across different datasets. Indeed, these methods lack the capacity to interpret molecular features within tissue morphology, limiting the understanding of biological and pathological processes at the cellular level. Consequently, there is a pressing need for a systematic and convenient approach for spatial pattern analysis.

**Fig. 1.**
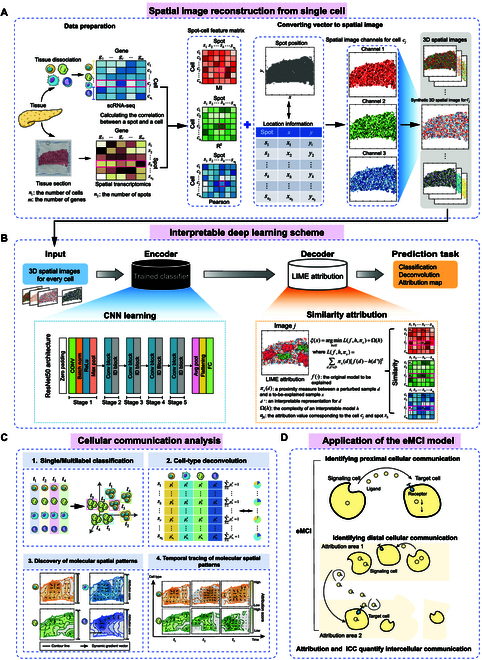
A schematic overview of the eMCI framework. (A) Spatial image reconstruction by integrating scRNA-seq and ST profiles obtained from the same or related tissues. eMCI initially calculates the correlation between spots and cells to generate 3 spot–cell feature matrices based on different correlation indices, including MI, *R*^2^, and the PCC. eMCI then converts the feature vectors into different channels for the cell *c_j_* according to the provided location information of each spot, reconstructing a 3-dimensional spatial image for the cell. (B) Schematic of the internal architecture of the classifier in eMCI, wherein the LIME algorithm is employed to interpret the classification outcome. (C) eMCI can be used for a series of downstream analysis: performing single/multilabel classification for scRNA-seq data (especially with temporal and cell-type information in this study); detecting cell-type deconvolution of ST data; constructing cell-type-specific attribution maps within a tissue to help the interpretation of molecular features in histomorphology; and further exploring the spatial–temporal cell signaling landscape within a tissue. (D) eMCI quantitatively measures intercellular communication networks based on cell-type-specific attribution maps and new developed ICC metric, to better elucidate underlying proximal and distal cellular interactions.

In this work, we present eMCI, an explainable multitask deep neural network framework, to integrate scRNA-seq and ST data based on the deep fusion of multimodal spot–cell correlation spectrums on the same ST slice (Fig. 1). ST data provide spatial location information of gene expression in tissue sections, with each spot typically containing mixed gene expression data from 10 to 20 cells [1]. Therefore, we hypothesize that the gene expression patterns in ST data are associated with those in single-cell data. To comprehensively explore the various types of relationships between the single-cell and ST data, eMCI employs multimodal correlation metrics [16–18], including Pearson correlation coefficient (PCC) measuring the linear relationship between variables, mutual information (MI) accounting for nonlinear dependencies, and coefficient of determination (*R*^2^) assessing how well the variability of one variable can be explained by the other in a linear model, to fuse single-cell data with ST into grayscale images in different channels. These grayscale images are then combined to generate a full-color RGB pseudo-image representation [19], thereby facilitating cell-type prediction by a Convolutional Neural Network (CNN) classifier [20]. Then, eMCI incorporates the Local Interpretable Model-agnostic Explanations (LIME) attribution algorithm [21] to detect crucial cellular signal transduction across the spatial domains, explaining specificity on both cell types and distributions. By applying eMCI to datasets from different tissues and species (including mouse cortex, zebrafish melanomas, soybean nodule maturation, and human embryonic lung) generated with different techniques (such as STARmap, 10x Genomics, and Stereo-seq), eMCI demonstrated better or similar performance compared with state-of-the-art methods in cell-type deconvolution and classification tasks. Based on the cell-type-specific attribution maps derived by eMCI, we developed an integrated correlation coefficient (ICC) measurement to quantify underlying cell–cell communication (CCC) within the biological microenvironment. From the perspective of ligand–receptor interactions in cell signaling analysis, eMCI can further infer the underlying dynamics of cellular signal transduction across spatial domains with statistical and biological validation. Noteworthily, eMCI is a flexible and scalable computational framework, which can be adapted to suit various research contexts. Its components, such as correlation metrics and image classifiers, are customizable, making it suitable for analyzing heterogeneous data and addressing diverse research needs. In summary, our analyses demonstrate that eMCI is a valuable tool for the integrative analysis of ST and scRNA-seq data, effectively elucidating intercellular communication without the need for biological prior reference.

## Results

### Accurate and robust performance of eMCI on cell-type classification compared to baseline methods

Cell-type classification is a critical task in various biological research areas, such as cell reprogramming and cancer studies [22]. eMCI integrates single-cell data with ST data to predict the cell types enriched in each spot, allowing for the full utilization of nonlinear biological association between cell clusters (types) and spatial spots.

On one hand, eMCI was evaluated using a multiclass classification task with a collection of 6 datasets from different biological samples (see details in Table S1), including human embryonic lung, human pancreas, pancreatic ductal adenocarcinoma (PDAC) and breast cancer (BRCA), which have been widely used in previous studies [23–26]. To establish evaluation benchmarks, 5 popular automatic cell-type classification methods based solely on scRNA-seq data were employed, including CaSTLe, CHETAH, clustifyr, scmap-cluster, and scmap-cell [27–30]. The average accuracy of each method over 10 iterations on each dataset is summarized in Table and Fig. S1. Remarkably, eMCI achieved cell classification accuracies exceeding 98% on the lung development dataset, 94.48% and 88.24% on the 2 pancreas datasets, and 86.01% and 95.03% on the PDAC and BRCA datasets, respectively. Overall, eMCI achieved the highest accuracy across nearly all datasets, with the exception of one BRCA dataset where its accuracy was 1% lower than the best-performing method. Moreover, the confusion matrices of the eMCI’s performance and the comparison of the classification accuracy of eMCI with 2 other benchmarking methods across different cell types for the lung development (6 postconception weeks [PCW]), pancreas (12 PCW), and PDAC datasets are provided in Figs. S2 to S4. The results showed that eMCI achieved over 90% accuracy for most cell types, consistently exhibiting higher and more concentrated accuracy results compared to the other methods. These results suggest that eMCI not only achieves higher overall accuracy but also demonstrates greater stability and reliability in its classification results.

**Table.** Comparison of the cell-type classification accuracy of eMCI and other existing methods in multiple datasets

Method datasets	eMCI	CaSTLe	CHETAH	clustifyr	scmap-cluster	scmap-cell
Lung 6 PCW	****99.08%** ^a^**	96.53%	92.92%	94.03%	93.74%	98.31%
Lung 7 PCW	****99.10%** ^a^**	98.66%	90.77%	97.14%	85.71%	98.26%
Lung 8 PCW	****98.51%** ^a^**	95.71%	94.88%	98.24%	90.44%	97.41%
Lung 8.5 PCW	****98.29%** ^a^**	96.44%	88.72%	96.45%	87.04%	96.67%
Lung 10 PCW	****98.28%** ^a^**	95.72%	94.05%	97.09%	82.97%	94.12%
Lung 11.5 PCW	****98.83%** ^a^**	98.36%	92.32%	98.58%	72.56%	96.74%
Lung 13 PCW	****98.78%** ^a^**	97.59%	89.56%	98.78%	87.85%	98.53%
Pancreas 12 PCW	****94.48%** ^a^**	87.62%	68.88%	85.73%	86.46%	91.27%
Pancreas 20 PCW	****88.24%** ^a^**	81.20%	79.55%	63.93%	87.95%	73.74%
PDAC	****86.01%** ^a^**	60.18%	64.20%	62.33%	82.95%	64.38%
BRCA	95.03%	95.12%	81.41%	91.64%	85.30%	****95.87%** ^a^**

^a^ The highest average accuracy over 10 iterations is marked by bold typeface.

On the other hand, we evaluated the performance of eMCI with the human embryonic lung datasets on a multilabel classification task. We integrated data from 7 stages of lung development, comprising a total of 52,732 cells and 7 tissue slices. Each cell was annotated with 2 labels (development stage and cell type). To accomplish multilabel classification across time and cell types, 70% of the annotated cells were allocated to the training set for model training. As a result, eMCI achieved 98.49% accuracy in the test data (Table S2). Additionally, when tested individually on 2 labels (development stage and cell type), the pretrained eMCI model fusing ST data achieved 100% accuracy for stage classification and 98.49% accuracy for cell-type classification (Table S2). Furthermore, the eMCI framework using only single-cell data as input was used as a comparable baseline (the details are provided in Figs. S5 and S6), achieving 94.71% accuracy, which is lower than the accuracy achieved by the eMCI framework integrating scRNA-seq and ST data.

These results suggest that eMCI can achieve higher accuracy than other methods in most datasets, which is attributed to the integration of scRNA-seq data and ST based on robust spot–cell correlation information rather than sparse gene expression information.

### Accurate and robust deconvolution performance of eMCI compared with existing methods

**Fig. 2.**
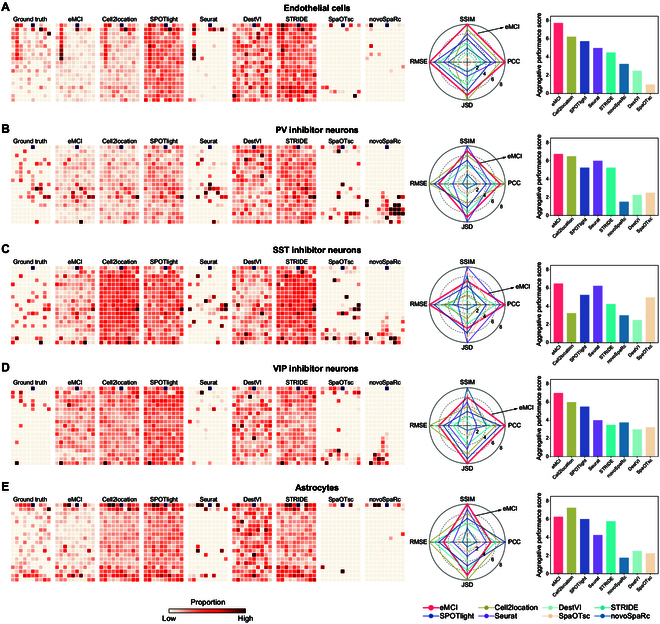
Performance comparison of 8 methods capable of cell deconvolution. (A to E) Left panel: The proportion of different cell types in the spots from mouse visual cortex dataset, including the ground truth and the predicted results of 8 methods. Right panel: The performance level of different benchmark metrics for deconvolution. The radar chart depicts the performance level of each method under specific quantitative metrics, ranging from 1 to 8. Specifically, the method with the highest PCC/SSIM value will have PL_PCC/SSIM_ = 8, and the method with the lowest PCC/SSIM value will have PL_PCC/SSIM_ = 1. The method with the highest RMSE/JSD value will have PL_RMSE/JSD_ = 1, and the method with the lowest RMSE/JSD value will have PL_RMSE/JSD_ = 8. The bar chart illustrates the APS values of each method in the deconvolution performance for a specific cell type.

We then evaluated the performance of eMCI in predicting the composition of cell types in individual ST spots compared with existing deconvolution methods, including Cell2location [8], SPOTlight [9], Seurat [11], DestVI [12], STRIDE [3], SpaOTsc [13], and novoSpaRc [14]. In such evaluation, the cell-type composition of each spot has been reported and can be used as the ground truth when simulating a dataset with potentially ambiguous cell-type assignations in each spot [7]. Here, PCC, structural similarity (SSIM), root mean square error (RMSE) and Jensen–Shannon divergence (JSD) metrics were employed as the performance evaluation measurements, and a higher PCC/SSIM or lower RMSE/JSD value for each cell type indicates a better deconvolution accuracy and results in a higher performance level in comparison. By aggregating these 4 evaluation metrics, an aggregative performance score (APS) was calculated (see Materials and Methods) to comprehensively evaluate the accuracy of various methods (i.e., a higher APS value indicates better global performance). According to results on multiple datasets (Fig. 2 and Fig. S7), eMCI intuitively performed better than other methods, attaining the highest APS values (Fig. 2A). Specifically, eMCI achieved higher performance levels than those of other 7 benchmarking methods on PCC, SSIM, and RMSE, and eMCI was only lower than that of Cell2location on JSD. More detailed numerical results of PCC, SSIM, RMSE, and JSD for eMCI and other deconvolution methods are provided in Table S3. Overall, eMCI has competitive performance compared to the state-of-the-art methods in predicting the spatial distribution of different cell types, which can provide consistent analysis efficiency of ST data and simultaneously help downstream analysis for relevant key molecules, ligands/receptors, and their dynamical interactions underlying spatial expression pattern.

### Efficiency of eMCI on detecting key molecules relevant to tumors

Based on its improved ability of cell-type classification and cell-type deconvolution, eMCI’s functional capabilities can be enhanced and demonstrated at both molecular and cellular levels.

**Fig. 3.**
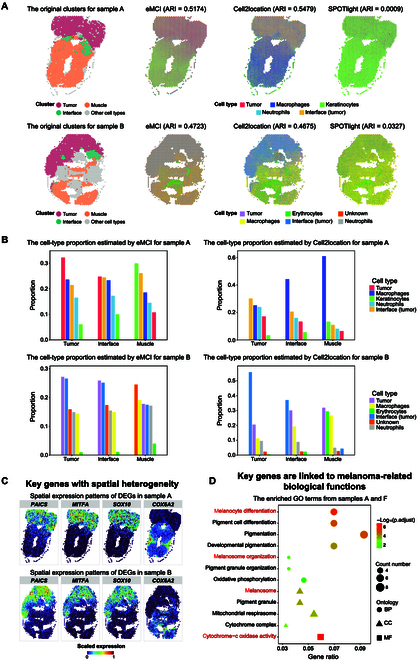
Deconvolution analysis of case study on zebrafish melanoma datasets. (A) Deconvolution performance of eMCI for 2 samples compared with Cell2location and SPOTlight. eMCI displayed comparable performance as Cell2location on cell composition inference. (B) Bar charts for the region-specific compositions of cell types derived by eMCI and Cell2location, respectively. eMCI demonstrates better identification of global and local cell distribution than Cell2location. (C) Spatial expression distribution of DEGs obtained based on the deconvolution results of eMCI. (D) Functional enrichment analysis for the DEGs from samples A and F. eMCI found many new molecules and functions relevant to melanoma, in addition to previous study and Cell2location analysis.

We carried out the first case study of eMCI on 2 paired datasets of zebrafish melanomas, including ST data from samples A and B, as well as scRNA-seq data from samples E and F. The Visium array spots were segmented into 4 regions in original study [31], including the tumor region, interface region, muscle region, and a region encompassing other cell types (Fig. 3A).

After determining the cell type from the scRNA-seq data (see details in Fig. S8), eMCI effectively characterized the spatial heterogeneity of cell-type composition (Fig. 3A and Fig. S9) in tissue slices A and B of zebrafish (adjusted rand index [ARI] = 0.5174/0.4723 for sample A/B), yielding results similar to those obtained with Cell2location (ARI = 0.5479/0.4675 for sample A/B). Further, the cell-type proportions of all spots within 4 regions were averaged to represent the region-specific cell-type composition. We found that, as inferred by eMCI, F-cluster 0 (tumor cells) and E-cluster 0 (tumor) exhibit the highest proportions in the tumor regions of ST samples A and B, respectively (Fig. 3B), validating the effectiveness of eMCI in robustly locating tumor cells across different samples. However, F-cluster 4 (interface (tumor) cells) and E-cluster 3 (interface (tumor) cells) occupy the highest proportions inferred by Cell2location in the tumor regions of samples A and B, respectively. The details of the matching relationship between clusters and cell-type annotations from the original study (Table S4) suggested the efficiency of eMCI for characterizing global and local spatial heterogeneity while Cell2location would miss details in the local spatial domain.

In addition, through differential expression analysis on the cell types that predominantly occupy the tumor and muscle regions based on the deconvolution of eMCI, some key genes with spatial heterogeneity of spatial expression patterns were detected, such as *PAICS*, *MITFA*, *SOX10*, and *COX6A2* (Fig. 3C). *MITFA* and *SOX10* have previously been identified as specific markers of malignant zebrafish melanomas in the original study [31], while *PAICS* and *COX6A2* are newly discovered by our study, which actually play important biological roles and molecular functions in tumor development [32,33]. According to functional enrichment analysis, these key genes were found to be mainly involved in biological functions related to melanoma, such as melanocyte differentiation (Gene Ontology [GO]:0030318), melanosome organization (GO:0032438), and oxidative phosphorylation pathway [34] (Fig. 3D and Figs. S10 and S11). For example, the overexpression of *PAICS*, which is involved in GO:0043473, in melanocytes could inhibit tumor cell apoptosis and promote the growth by regulating related signaling pathways [32]; and *COX6A2* in GO:0004129 is also implicated in the pathogenesis of melanoma through the cytochrome *c* oxidase subunit [33]. Besides, oxidative phosphorylation plays an important role in facilitating the invasive growth of primary melanoma [34]. As a comparison, the similar analysis based on the deconvolution from Cell2location has led to the findings of biological processes related to DNA replication and cell cycle (Table S5). These results strongly indicated that eMCI can capture key genes and pathways associated with tumorigenesis specificity more effectively than conventional methods, enabling the comprehensive characterization of spatial heterogeneity of tumor cells and molecules within the pathogen tissue context.

### Efficiency of eMCI on uncovering spatial expression patterns of ligands/receptors based on the interpretable attribution maps

The second case study of eMCI was carried out on a paired snRNA-seq and ST dataset of soybean, where the ST was acquired by Stereo-seq. The ST dataset consists of 4 sections, including 2 sections from the 12-days postinfection (12 dpi) nodule and 2 sections from the 21-days postinfection (21 dpi) nodule, whereas the snRNA-seq dataset includes expression profile data from nodules at 12 and 21 dpi, respectively. The clustering and annotation results of snRNA-seq and Stereo-seq datasets were retained from the original publication [35].

**Fig. 4.**
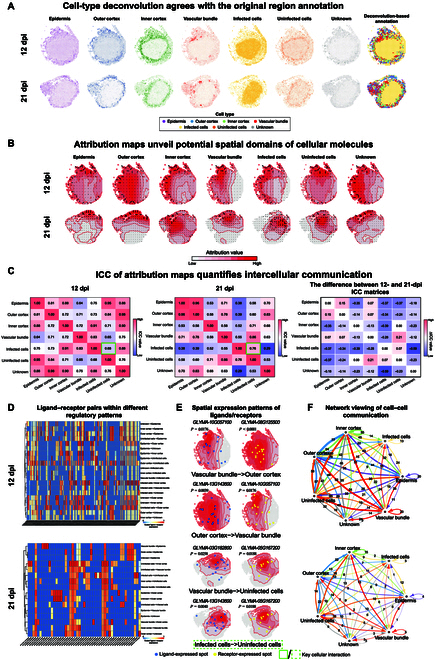
Case study of soybean nodule maturation by eMCI. (A) The cell-type deconvolution by eMCI for the 12- and 21-dpi nodules. (B) The cell-type-specific attribution maps with dynamic gradient field derived by eMCI for the 12- and 21-dpi nodules. (C) ICC quantifies the similarity of cell-type-specific attribution maps at 12 and 21 dpi. PlantPhoneDB validated the attribution-based similarity from a biological perspective. (D) A diverse range of ligand–receptor pairs shows different regulatory patterns. Columns are scaled by max ligand–receptor expression. (E) The spatial expression patterns of certain pairwise ligands and receptors within intercellular interactions are significantly associated with the attribution maps (*P* < 0.05). Blue dots represent expressed spots. (F) Network viewing of CCC between pairwise cell types.

First, the cell-type deconvolution for all the soybean ST data was obtained by eMCI (Fig. 4A and Fig. S12); the map of cellular spatial distribution on the far right is determined by the predominant cell type in each spot, roughly consistent with the annotation for ST data in the original publication.

Next, eMCI performed attribution analysis for each cell image and aggregate the cell images based on cell type to produce cell-type-specific attribution maps, identifying key spatial segmentations responsible for different cell types. The regions with high attribution values are considered to be important regions for the corresponding cell type, annotated as attribution maps. For each cell type, its attribution map is summarized in Fig. 4B. An interesting observation is that for a specific cell type, the attribution distribution would differ from its spatial distribution. Meanwhile, there is an overlap between the attribution distribution and the spatial distribution to some extent (see details in Table S6). This fact indicates the potential contribution of neighboring cells/spots on particular cell-type recognition and distribution in spatial locations, which is just a key focus of cellular communication considered by the model and analysis of eMCI.

The similarity of between different cell-type attribution maps aggregating individual cell attribution maps is further quantified from a statistical perspective by calculating the ICC (see details in Materials and Methods), where a larger ICC value indicates a greater similarity between 2 attribution maps. Such a combined correlation measurement has been validated to be effective through an ablation study (see more detailed results in Fig. S13 and Table S7). We found that some ICC values for cell-type-specific attribution maps across various time points increased, such as ICC (∆ICC = 0.21) between uninfected and vascular bundle cells, while some ICC values decreased across various time points, such as ICC (∆ICC =  −0.12) between vascular bundle and unknown cells, consistent with the observation in the other replicate sections (Fig. 4C and Fig. S12C). Notably, ICC between infected and uninfected cells slightly increased (∆ICC = 0.07), and the same trend was observed in the other replicate sections (∆ICC = 0.42), indicating the possible spread and invasion of infected cells at 21 dpi.

Subsequently, we aimed to validate such attribution-based similarity from a biological perspective by CCC, based on the CCC analysis on snRNA-seq data using PlantPhoneDB (see Materials and Methods). Notably, many cellular communications involving a diverse range of ligand–receptor pairs align with the high ICC observed in the aforementioned attribution-based similarity, such as intercellular communications between outer cortex cells and inner cortex cells, as well as uninfected cells at 12 dpi (Fig. 4D). Furthermore, the spatial expression patterns of certain ligand–receptor pairs in intercellular communication exhibit significant association (*P* < 0.05) to the attribution maps with dynamic gradient fields (Fig. 4E). The hypergeometric test (see Materials and Methods) statistically validated the relevance of the cell-type-specific attribution maps and spatial expression patterns of ligands and receptors within the tissue context, inspired by multimodal intersection analysis [36]. For example, the ligand *GLYMA-10G057100* (*P* = 0.0176) and receptor *GLYMA-08G125500* (*P* < 0.0001) participate in the intercellular communication from vascular bundle to outer cortex cells; the ligand *GLYMA-13G143600* (*P* = 0.0039) and receptor *GLYMA-10G057100* (*P* = 0.0176) are involved in the intercellular communication from outer cortex to vascular bundle cells at 12 dpi; the ligand *GLYMA-03G182800* (*P* = 0.0258) and receptor *GLYMA-05G167200* (*P* = 0.0188) play intercellular communication roles between vascular bundle and infected cells; and the ligand *GLYMA-13G143600* (*P* = 0.0040) and receptor *GLYMA- 05G167200* (*P* = 0.0188) contribute to the intercellular communication between infected and uninfected cells at 21 dpi.

Moreover, the CCC network at 21 dpi showed a marked reduction in the number of ligand–receptor pairs compared to the network at 12 dpi (Fig. 4F). This indicated a weakening of intercellular interactions and instability of the network structure over the course of postinfection time, validating the effectiveness of the differential ICC matrix with a predominance of negative values (70% ΔICC < 0). Additional analytical details of eMCI across the entire dataset, including other sections, are provided in Figs. S12, S14, and S15. These results suggest that eMCI could effectively characterize spatial expression patterns of cell signaling between proximal and distal cells within the spatial context, even without the guidance of prior-knowledge of ligands/receptor information.

### Efficiency of eMCI on characterizing temporal–spatial expression patterns of intercellular communication based on dynamical attribution maps with multilabel prediction

For the complicate case study of eMCI on human embryonic lung development data, we have utilized both scRNA-seq and ST data of human embryonic lung at multiple developmental stages at 6, 7, 8, 8.5, 10, 11.5, and 13 PCW, including a total of 52,732 cells and 7 tissue slices [37].

**Fig. 5.**
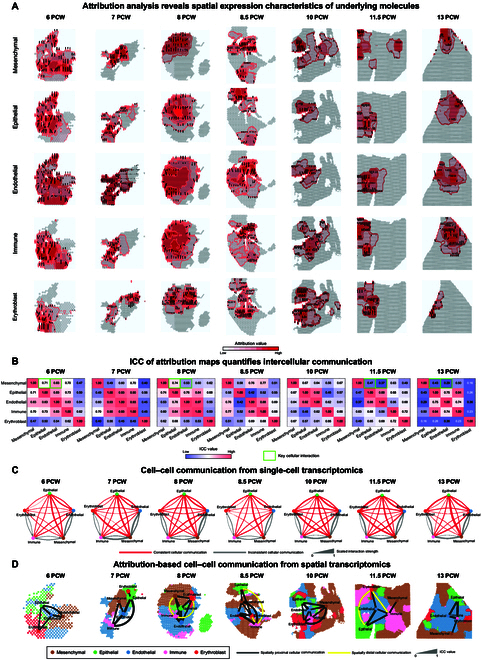
Case study of human embryonic lung development by eMCI. (A) The cell-type-specific attribution maps with dynamic gradient field derived by eMCI for each cell type at different stages. (B) ICC quantifies the similarity of cell-type-specific attribution maps at different stage, revealing the dynamic evolution of essential cellular interactions over the lung development. (C and D) Systematic analysis of CCC from single-cell and spatially resolved transcriptomics, respectively. (C) The dynamic trends of most cellular interaction strengths (colored by red) inferred by CellChat v2 are positively correlated with those of the corresponding ICC values during the pseudoglandular stage. (D) Attribution and ICC-based CCC analysis effectively revealed proximal–distal patterning within the spatial context during the human lung development.

After conducting eMCI in complete multitask analysis (see deconvolution results in Fig. S16), we calculated cell-type-specific dynamical attribution maps at each stage (Fig. 5A), characterizing temporal–spatial expression patterns of key molecules for 5 main cell types at individual stage, including mesenchymal, epithelial, endothelial, immune, and erythroblast/red blood cells (see more details in Fig. S17).

Next, we calculated the ICC values among attribution maps of various cell types at different stages (Fig. 5B), the ICC values across different stages exhibit dynamic changes of intercellular interactions or other biological processes during lung development (see more details in Fig. S18). For example, the correlation matrices representing intercellular communication at 6 and 7 PCW, as well as those at 10 and 11.5 PCW, appear remarkably similar intuitively, with the quantification of similarity for these correlation matrices depicted in Fig. S19.

Then, for mesenchymal cells, we performed a differential expression analysis for ST data between its specific high-attribution spots and other spots, along with functional enrichment analysis on corresponding differentially expressed genes (DEGs) at each stage. During the early stages of the embryonic lung development (e.g., at 6 and 8 PCW), there were up to 27 enriched GO terms related to endothelial and epithelial cells, including endothelium development, endothelial cell differentiation, regulation of epithelial cell proliferation and epithelial cell migration, and numerous GO terms related to mesenchymal cells, such as embryonic organ development and embryonic organ morphogenesis (Table S1). This suggested that ICC matrices revealed close cell–cell association between focused mesenchymal cells and other endothelial and epithelial cells (Fig. 5B), which are consistent with the developmental program underlying distal patterning of the human lung reported by other study [38]. By contrast, as lung development progressed into later stages, the number of enriched functions related to endothelial and epithelial cells drastically decreased, suggesting that the connection between mesenchymal cells and both endothelial and epithelial cells became weak at later development stage, consistently reflected by the changes of ICC values in Fig. 5B (e.g., 0.37/0.47 for endothelial/epithelial cells at 11.5 PCW and 0.29/0.43 for endothelial/epithelial cells at 13 PCW). Indeed, numerous studies have highlighted that mesenchymal–epithelial and mesenchymal–endothelial interactions result from the coordinated activity of multiple signaling pathways [39,40]. During the pseudo glandular stage of lung development, the activity and intensity of these pathways, such as the Sonic hedgehog [39] and fibroblast growth factor [40] signaling pathways, gradually diminish over time, remarkably aligning with the observation from new ICC matrices provided by eMCI.

Besides, we performed the CCC analysis by CellChat v2 based on the cell type and distribution from eMCI to seek additional reasonability of intercellular interactions for above dynamical attribution maps (see more details in Materials and Methods). Notably, the dynamic trends of some cellular interaction strengths inferred by CellChat v2 are positively correlated with those of the corresponding ICC values for the stage (Fig. 5C and Fig. S20), where these interactions highlighted by red edges validated again the effectiveness of eMCI in uncovering proximal intercellular interactions, such as mesenchymal–epithelial communication [38] (see more computational details in Tables S8 and S9).

Notably, the ICC’s changing trends of other cell communications, which are negatively correlated with those in CellChat v2, might provide new insights for revealing distal cellular interactions within the spatial context, such as the attribution-based CCC networks from ST data in Fig. 5D. On one hand, there are shared spatially proximal CCCs from different methods, related to human embryonic lung development. For example, at 6 PCW, there are observable CCCs among mesenchymal, epithelial, and endothelial cells, consistent with our ICC matrix in Fig. 5B. On the other hand, eMCI can identify some spatially distal cellular communications that are not easily recognizable by conventional CCC methods but really exist in the biologically intercellular communication network (e.g., yellow edges), such as the mesenchymal-immune [41] and immune-erythroblast [42] intercellular interactions at 8 PCW (with ICC values 0.60 and 0.53, respectively). As previously reported, such spatially distal cellular communications over long spatial distances may be achieved by extracellular vesicles traversing the extracellular matrix [43].

Together, taking advantage of the lung development datasets, eMCI can provide systematic analysis of proximal and distal CCCs and expand the scope of human lung developmental biology at early pseudoglandular stages, deepening the understanding of proximal–distal patterning in human lung development and opening up new avenues for regenerative medicine.

## Discussion

Along with the advancements in spatial and single-cell transcriptome sequencing technologies, the accumulation of large single-cell and ST data has facilitated the characterization of the intercellular molecular information flow within tissue morphology. This endeavor is crucial for unveiling potentially cell-type-specific intermolecular signaling and its relevance across multimodality biological data. In this work, we propose eMCI, an explainable multitask deep learning model, for the integrative analysis of scRNA-seq and ST data from the same tissue. Specifically, eMCI integrated multimodal biological data into pseudo-images by preserving spatial information and utilizing multiple correlations, which allowed the CNN to extract deeper patterns and improve cell-type classification. By applying LIME for interpretability, eMCI identified key spatial domains enriched with cell-type-specific information. Furthermore, using ICC to analyze the colocalization of cell-type-specific attribution maps, eMCI inferred potential molecular communication between cells, with higher ICC values indicating stronger colocalization between the cell-type-specific information and increased likelihood of biological interactions. As a result, eMCI can achieve cell-type classification, cell-type deconvolution, and recognition of cell-type-specific molecular spatial patterns in a unified framework. The detailed descriptions for a specific example that demonstrates how eMCI identifies key spatial expression patterns and intercellular interactions are provided in Note S1.

By conducting comparisons between eMCI and other state-of-the-art methods across datasets from different tissues, including PDAC, BRCA, human pancreas, and embryonic lung, we confirmed the stable and outstanding performance of eMCI for cell-type classification across diverse biological microenvironments. Particularly, eMCI also has shown exceptional performance in multilabel cell-type classification tasks, underscoring its robustness in more complicated application scenarios. Noteworthily, scRNA-seq and ST represent 2 distinct data modalities, and it is inevitable that some degree of batch effect exists between them [44]. We strive to select paired single-cell and ST data from the same donor and the same tissue, thereby eliminating sample batch effects. For technical batch effects, we compared the performance of eMCI on raw count data with its performance on batch-corrected data of the lung development (6 PCW), pancreas (12 PCW), and PDAC datasets (Fig. S21), with transfer component analysis as the batch-correction method [45]. The results showed that batch correction effectively improved classification accuracy, though the improvement was not statistically significant (*P* = 0.1306), indicating that batch effects are indeed non-negligible and should be carefully considered. In addition, we have conducted benchmark experiments on the datasets with different sequencing quality (i.e., about 3,000 genes, about 5,000 genes, and about 7,000 genes) to assess how eMCI depends on the sequencing quality of both ST and scRNA-seq datasets. The results indicated that eMCI maintains robust performance under different levels of sequencing quality (Fig. S22). We have also compared eMCI’s performance on the paired single-cell and ST data from the same donor with the paired data from different donors and found that eMCI still achieved strong accuracy (Fig. S23), indicating that eMCI can also work well with scRNA-seq and ST data of separate donors.

Next, we benchmarked eMCI’s performance of cell-type deconvolution within the spatial context not only on simulated data but also on several actual biological datasets from diverse species, such as zebrafish and soybean. Besides, we conducted ablation experiments for the cell-type deconvolution and classification tasks, comparing the performance of the multiple correlations used in eMCI with that of individual correlation metrics, as well as Kullback–Leibler (KL) divergence and the Wilcoxon rank-sum test. The results indicate that the multiple correlations used in eMCI consistently outperformed other individual metrics in overall performance (Figs. S24 and S25), particularly KL divergence and the Wilcoxon rank-sum test, across all cases, validating the effectiveness and robustness of our approach in cell-type deconvolution and classification. Further, we also demonstrated the comparably good and robust performance of eMCI in deriving spatial distributions of distinct cell types in different biological systems. On one hand, by utilizing our developed ICC measurement and PlantPhoneDB information, we demonstrate eMCI’s ability to elucidate patterns of molecular information flow in cell communication. For example, the attribution maps of soybean are biologically and statistically validated through the spatial expression patterns of ligands and receptors. On the other hand, we demonstrated the accurate identification of spatial domains by eMCI and its potential for understanding tissue organization and biological functions. For instance, the inference of spatially proximal communication between cell types by eMCI using ICC matrix is consistent to that by CellChat v2 in human embryonic lung, supported by statistical and biological validation. Noteworthily, eMCI can not only elucidate global intercellular communication within the spatial context but also detect the colocalization of distinct cell types within local pockets. Specifically, LIME, which serves as the attribution method of eMCI, explains the model’s predictions in a localized scope around the input. When applied to local regions of the spatial context, eMCI computes ICC values between the local attribution maps generated by LIME, thereby aiding in the identification of local colocalization of distinct cell types. Moreover, with information from multiple cell subtypes, eMCI can calculate the ICC between subtype-specific attribution maps to infer the strength of communication between these subtypes, allowing for a more detailed characterization of cellular communication across the spatial domain.

Depending on the unified computational framework, eMCI has marked advantages in several aspects. First, for the cell-type classification task, eMCI capitalizes on the complementary characteristics of scRNA-seq and ST data, outperforming the methods solely relying on single-cell data in terms of classification accuracy. Second, for the cell-type deconvolution task, eMCI employs various linear and nonlinear correlation metrics to fuse single-cell data with ST into a new data space as pseudo-images, dissecting spatial spot–cell comodules and enhancing the interpretability and robustness of cell-type deconvolution. Third, eMCI leverages explainable learning methods to overcome the black-box nature of CNNs, characterizing cell-type-specific molecular information flow in tissue contexts and enabling a more comprehensive understanding of cellular interplay and region-specific physiological/pathological changes within the spatial context. Of note, many CCC methods limit their analysis to proximal cells and spots based on given ligand/receptor references, by contrast, eMCI can effectively elucidate more candidates of proximal and distal intercellular communication without biological prior knowledge.

Despite the aforementioned merits, eMCI still requires some potential enhancements in the future, considering the rapid development of ST and scRNA-seq techniques. Similar to many existing methods, cell deconvolution by eMCI depends on the assumption that cell populations are similar or consistent between ST and scRNA-seq data; thus, it is challenging when estimating the fractions of cell types present in ST data but absent in scRNA-seq data. In addition, eMCI is designed as a general computational framework with scalability and flexibility. Given the heterogeneity and complexity of real-world data, researchers can adapt components of this framework, such as correlation metric selection and image classifiers, to better suit their specific research objectives and contexts. This approach accommodates diverse research needs and opens new pathways for exploring and analyzing heterogeneous data. Although currently we can select various correlation metrics to implement the proposed algorithm depending on specific usage scenarios and actual data characteristics, we anticipate that eMCI could be further enhanced through learning of correlation metrics and image classifiers in different tasks by ongoing effort in deep learning method developments. Finally, with the aid of data from different modalities (e.g., genomics, proteomics, and metabolomics) [46], eMCI could provide a more comprehensive understanding of cellular heterogeneity, interplay, and molecular regulatory mechanisms in the spatial context, following the central dogma.

## Materials and Methods

### Data preprocessing

For all datasets, we initially filtered out genes with low expression levels (expressed in fewer than 5% of cells or 1% of spots) from both the single-cell and ST data and subsequently extracted the expression matrices of the common genes as the input of the eMCI workflow.

### Workflow of eMCI

#### Multimodal similarity correlation metrics calculation

The relationship between single-cell data and ST is complex and multifaceted. ST data provide spatial location information of gene expression in tissue sections, with each spot typically containing mixed gene expression data from 10 to 20 cells [1]. Generally, similarity measurements based on correlation coefficients between commonly examined genes in both ST data and scRNA-seq data were used to reconstruct spatial gene expression or map cells in scRNA-seq data to their potential spatial origins [13,47]. Therefore, we hypothesize that the expression distribution of a spot reflects a combination of the distributions of the various cells it contains. If a spot predominantly contains cells from the same type, the gene expression distribution of that spot will resemble the distribution of a single cell from that same population. To comprehensively explore the various types of relationships between the single-cell and ST datasets, we chose to employ multiple correlation metrics. In this study, given a preprocessed paired dataset containing single-cell/single-nucleus RNA sequencing data with *n*_1_ cells and ST data with *n*_2_ spots, we measure the relationship between each cell from scRNA-seq data and each spot from ST data with different correlation metrics, including MI, *R*^2^, and PCC (Fig. 1A). Different metrics can provide complementary perspectives on the data, enhancing the robustness and depth of our analysis. The details are as follows:

1. MI, a commonly used correlation analysis tool based on information theory, captures linear and nonlinear dependence between 2 random variables. MI is zero when variables are independent, and it increases as dependency increases. For a cell *c_j_* and spot *s_k_* (*j* = 1, ⋯, *n*_1_ and *k* = 1, ⋯, *n*_2_), their gene expression vectors are denoted as $U_{j}=\left(u_{1}^{\;j},u_{2}^{\;j},\ldots ,u_{m}^{\;j}\right)$ and $V_{k}=\left(v_{1}^{k},v_{2}^{k},\ldots ,v_{m}^{k}\right)$, respectively, where *m* represents the number of genes. The MI between ***U****_j_* and ***V****_k_* is defined as the following form:
  $$w_{1}^{\;\mathrm{jk}}=\text{MI}\left(U_{j},V_{k}\right)=\sum_{i=1}^{m}\sum_{i=1}^{m}p\left(u_{l}^{\;j},v_{i}^{k}\right)\text{log}\frac{p\left(u_{l}^{\;j},v_{i}^{k}\right)}{p\left(u_{l}^{\;j}\right)p\left(v_{i}^{k}\right)}$$ (1)
  where $p\left(u_{l}^{\;j},v_{i}^{k}\right)$ is the joint probability mass function of ***U****_j_* and ***V****_k_*, and $p\left(u_{l}^{\;j}\right)$ and $p\left(v_{i}^{k}\right)$ are the marginal probability mass functions of ***U****_j_* and ***V****_k_*, respectively. In light of another study [48], the above formulation can be expressed as follows.
  $$\text{MI}\left(U_{j},V_{k}\right)=\frac{1}{2}\text{log}\frac{\left|C\left(U_{j}\right)\right|\cdot \left|C\left(V_{k}\right)\right|}{\left|C\left(U_{j},V_{k}\right)\right|}$$ (2)
  where *C*(***U****_j_*)/*C*(***V****_k_*) is the covariance matrix of the vector ***U****_j_*/***V****_k_*, |*C*| is the determinant of matrix *C*, and *C*(***U****_j_*, ***V****_k_*) is the covariance matrix between the vectors ***U****_j_* and ***V****_k_*.
2. Coefficient of determination, often denoted as *R*^2^, is a statistical measure representing the proportion of the variation in the dependent variable that is predictable from the independent variable in a regression model, quantifying the strength of the relationship between the independent and dependent variables. The *R*^2^ normally ranges from 0 to 1, where high *R*^2^ values indicate a strong relationship. The *R*^2^ between ***U****_j_* and ***V****_k_* is defined as the following form:
  $$w_{2}^{\;\mathrm{jk}}=R^{2}\left(U_{j},V_{k}\right)=1-\frac{\sum_{i=1}^{m}{\left(u_{i}^{\;j}-v_{i}^{k}\right)}^{2}}{\sum_{i=1}^{m}{\left(u_{i}^{\;j}-{\bar{U}}_{j}\right)}^{2}}$$ (3)
  where ${\bar{U}}_{j}$ is the mean of the gene expression vector ***U****_j_*.
3. Pearson correlation coefficient, denoted as PCC, focuses exclusively on the level of linear dependence between pairs of variables, and has range of −1 (perfect but negative linear relationship) to +1 (perfect and positive linear relationship) with 0 denoting the absence of a linear relationship. PCC measures the similarity of trend, removing the dimensional difference of different variables in the calculation process. The PCC between ***U****_j_* and ***V****_k_* is defined as the following form:
  $$w_{3}^{\;\mathrm{jk}}=\text{PCC}\left(U_{j},V_{k}\right)=\frac{1}{m-1}\sum_{i=1}^{m}\left(\frac{u_{i}^{\;j}-{\bar{U}}_{j}}{\sigma_{U_{j}}}\right)\left(\frac{v_{i}^{k}-{\bar{V}}_{k}}{\sigma_{V_{k}}}\right)$$ (4)
  where ${\bar{U}}_{j}/\sigma_{U_{j}}$ and ${\bar{V}}_{k}/\sigma_{V_{k}}$ are the mean/standard deviation of expression vectors $U_{j}$ and $V_{k}$, respectively.
  Therefore, the multimodal similarity correlation matrix $W_{i}={\left(w_{i}^{\;\mathrm{jk}}\right)}_{n_{1}\times n_{2}}\left(i=1,2,3\right)$ containing *n*_1_ rows and *n*_2_ columns was calculated by the above methods.

#### Pseudo-images generalization

All elements of the row vector $w_{i}^{\;j}$ of *W_i_*(*i* = 1, 2, 3 and *j* = 1, 2, ⋯, *n*_1_) were mapped to the spatial positions of corresponding spots on the spatial scatter plot as pixels, generating a grayscale image *z_ij_* in one channel for cell *c_j_* (note: each row represents the correlation between a cell and all spots from one spatial image) (Fig. 1A). Subsequently, we merge grayscale images under 3 channels *z_ij_* (*i* = 1, 2, 3; *z*_1*j*_, *z*_2*j*_, *z*_3*j*_ represent grayscale images in the red, green and blue channels, respectively) to compose one 3-dimensional RGB full-color image *Z_j_* (*j* = 1, 2, 3, …, *n*_1_):

$$Z_{j}=\overset{3}{\underset{i=1}{⋃}}z_{\mathrm{ij}}$$ (5)

the above pseudo image *Z_j_* can serve as input for a CNN classifier.

#### CNN model configuration of eMCI by residual network

In this study, a CNN architecture was utilized to process the pseudo-image *Z_j_* (Fig. 1B). The default CNN model adopted a parallel ResNet50 architecture, consisting of convolutional layers, identity blocks, convolutional blocks, and fully connected layers. The convolutional layers extracted features from the input image through convolution operations followed by batch normalization and ReLU activation. Max pooling layers following the convolutional layers reduced the spatial dimensions while retaining important features. Identity blocks facilitated learning residual functions, and convolutional blocks included 1 × 1 convolutional layers to reduce filter numbers. Fully connected layers performed final classification using softmax activation. The architecture had few hyperparameters, with “MaxEpochs” fixed at 100 by default. Specifically, the ResNet50 architecture comprised 53 deep convolutional layers. The initial layer included a 7 × 7 convolutional layer (with a stride of 2) followed by a 3 × 3 max-pooling layer (with a stride of 2). This was succeeded by 4 groups of residual blocks containing 3, 4, 6, and 3 residual units, each unit comprising 1 × 1, 3 × 3, and 1 × 1 convolutional layers. Each convolutional layer was immediately followed by a ReLU activation function and batch normalization to enhance training stability and accelerate convergence. Skip connections were employed to mitigate the vanishing gradient problem [20], with He initialization ensuring effective signal propagation through the deep network [20]. In this study, training was performed using the stochastic gradient descent optimizer, with an initial learning rate of 0.00005 and a momentum factor of 0.8. Additionally, L2 regularization (with a weight decay of 0.0125) was applied to improve model generalization and optimize performance. The network concluded with a global average pooling layer that was connected to a fully connected layer with multiple output classes. The detailed architecture is described in Fig. S26.

Indeed, ResNet50 serves as the primary neural network model for our study here, due to its established performance in image classification. Meanwhile, other state-of-the-art CNNs like EfficientNet [49] and Scorenet [50] are also compatible options, given the framework’s flexibility of eMCI.

#### Interpretable model component of eMCI by the LIME algorithm

Various feature attribution methods have been proposed to explain deep neuronal networks by inferring the attribution score of each input variable to the final output, which provides a map guidance ability to trace molecular functional changes [21]. As a popular interpretability method, LIME can explain the CNN’s predictions by fitting a linear model in the local scope around the input, helping to identify the most influential features (spatial domains in the spatial images) contributing to each cell-type prediction (Fig. 1B). One can consider these influential spatial regions identified by LIME to be the areas most enriched with cell-type-specific information. By highlighting these regions, we can better understand the spatial domains that play a critical role in each cell-type prediction. In eMCI, cell-type-based attribution score maps are calculated using the LIME algorithm to deduce the contribution or importance of each spot in an input image to the classification outcome (Fig. 1B). Specifically, for an input image *Z_j_*, LIME first generates a new dataset consisting of numerous perturbations of *Z_j_* and the corresponding predictions of the CNN model *f*(·) by turning super-pixels of *Z_j_* on and off. It then trains a simpler interpretable model (decision tree in this work) *h*(·) on this dataset, weighting the perturbations based on their proximity to *Z_j_* to ensure that drastic perturbations have little impact. Finally, attribution values are generated when this interpretable model locally approximating the CNN model, thus creating an explanation for *Z_j_* and identifying which positions of the image play important roles in the classification decision. We utilized the imageLIME function in MATLAB (https://ww2.mathworks.cn/products/matlab.html) for explanation generation, with all parameters set to default. The mathematical formulation for LIME is defined as follows.

$$\xi \left(x\right)=\underset{h\in G}{\text{arg}\;\text{min}}\;L\left(f,h,\pi_{x}\right)+\Omega \left(h\right)$$ (6)

where:

$$L\left(f,h,\pi_{x}\right)=\sum_{d,d^{\prime }\in D}\pi_{x}\left(d\right){\left[f\left(d\right)-h\left(d^{\prime }\right)\right]}^{2}$$ (7)

*x* is a to-be-explained instance image, and *D* is the generated dataset containing perturbed samples with the associated labels. *d* and $d^{\prime }$ are the original and interpretable representations of a perturbed sample. *G* is a collection of potential interpretable models, such as feasible decision trees in this work. *π_x_* acts as proximity measure between a perturbed sample and *x*. Ω(*h*) is a measure of complexity of the interpretation *h* ∈ *G*. We consider an interpretable model *h* for the sample *x*, which aims to minimize the loss *L*(*f*, *h*, *π_x_*) while maintaining simplicity. For the image *Z_j_*, we denote the attribution matrix derived by LIME as *A_j_* = *ξ*(*Z_j_*), where each value represents the importance of the spot at corresponding position to the model. Given a cell type *C_t_* ∈ {*C*_1_, …, *C_q_*} (*q* is the total number of cell-type classes in the dataset), for the images with the same class label *C_t_*, we calculate the average attribution value of different images at each spot, thus generating a cell-type-specific attribution matrix ${\bar{A}}^{\;\left(t\right)}$. When each average attribution value in ${\bar{A}}^{\;\left(t\right)}$ is mapped to its corresponding position within a tissue image, an attribution map specific to the cell-type *C_t_* is generated. This map provides a quantitative measure for visualizing the contribution of spatial features to cell-type-specific observations. Contour lines on the map connect points of equal attribution value, while arrows indicate the directions of the attribution-based dynamic gradient field.

#### Deconvolution application by eMCI

Cell proportion in each spot is estimated by multimodal similarity correlation matrix *W_i_* (*i* = 1, 2, 3) (Fig. 1C). Specifically, we have computed the average of 3 spot–cell feature matrices, denoted as $\bar{W}$, where ${\bar{w}}^{j}=\frac{1}{3}\sum_{i=1}^{3}w_{i}^{\;j}$ represents the row vector corresponding to cell *c_j_* in $\bar{W}$. Subsequently, we further averaged the row vectors with the same cell-type label in $\bar{W}$, thus yielding a comprehensive correlation measure for each cell type within every spot. The mathematical formulation is depicted as follows:

$${\hat{w}}^{\left(t\right)}=\frac{1}{n_{t}}\sum_{c_{j}\in C_{t}}{\bar{w}}^{\;j}$$ (8)

where *n_t_* represents the number of cells with cell-type label *C_t_* ∈ {*C*_1_, *C*_2_, …, *C_q_*}, and ${\hat{w}}^{\left(t\right)}$ represents the average row vector with the comprehensive correlation measure corresponding to the cell type *C_t_*. Given cell types {*C*_1_, *C*_2_, …, *C_q_*}, we normalize the comprehensive correlation value to obtain the cell-type composition within each spot as follows:

$$p_{k}^{\left(t\right)}=\frac{{\hat{w}}_{k}^{\left(t\right)}}{\sum_{j=1}^{q}{\hat{w}}_{k}^{\left(j\right)}}$$ (9)

where ${\hat{w}}_{k}^{\left(j\right)}$ represents the element corresponding to the spot *s_k_* in ${\hat{w}}^{\left(j\right)}$, and $p_{k}^{\left(t\right)}$ represents the proportion of cell type *C_t_* within spot *s_k_*, satisfying $\sum_{t=1}^{q}p_{k}^{\left(t\right)}=1$. This is not a direct measure of mRNA fraction, but rather an inferred estimate, allowing us to estimate the relative abundance of mRNA from each cell type within the spot, under the assumption that the cell type with the highest correlation score is most likely to contribute the most mRNA.

#### Benchmarking eMCI with existing computational methods in cell deconvolution

To evaluate the cell deconvolution performance of eMCI, we compared it with the following existing 7 methods.

• Cell2location is a Bayesian model designed to delineate detailed cell types within ST data and generate comprehensive cellular maps across various tissues [8].

• SPOTlight is a deconvolution algorithm that builds upon a non-negative matrix factorization regression algorithm [9].

• Seurat computes the importance score reflecting the presence of various cell types within each spot based on enrichment analyses [11].

• DestVI is a Bayesian method based on a conditional deep generative model for multiresolution deconvolution of cell types in ST data [12].

• STRIDE is designed to deconvolve the cell-type composition of ST locations by topic modeling [3].

• SpaOTsc recovers spatial properties of scRNA-seq data based on structured optimal transport [13].

• novoSpaRc is a computational framework that probabilistically assigns cells to tissue locations [14].

#### Data preparation for benchmarking of cell deconvolution

To benchmark the performance of deconvolution algorithms, we used a single-cell resolution ST dataset from mouse visual cortex [7], which was originally acquired by STAR-map [7], including 1,549 cells that correspond to 15 cell types, and was gridded into 189 square pseudo-spots with each containing 1 to 18 cells, to simulate the “multicell spot” like ST datasets. The expression profiles of all cells in each grid were summed up as the expression vector of each pseudo-spot, with the center point of the grid used as the new coordinate. The scRNA-seq dataset based on Smart-seq from [7] was used as a reference.

#### Benchmark metrics for evaluating eMCI’s performance on ST data

We used the following 5 metrics to evaluate the performance of the deconvolution methods for simulated data.

1. PCC. The PCC value between the predicted result and the ground truth of cell type *C_t_* was calculated using the following equation:

$$\text{PCC}\left(p^{\left(t\right)},{\tilde{p}}^{\;\left(t\right)}\right)=\frac{1}{n_{2}-1}\sum_{k=1}^{n_{2}}\left(\frac{p_{k}^{\left(t\right)}-\mu_{t}}{\sigma_{t}}\right)\left(\frac{{\tilde{p}}_{k}^{\;\left(t\right)}-{\tilde{\mu }}_{t}}{{\tilde{\sigma }}_{t}}\right)$$ (10)

where *n*_2_ is the total number of spots; ***p***^(*t*)^ and ${\tilde{p}}^{\left(t\right)}$ are the spatial cell-type proportion vectors of cell type *C_t_* in the predicted result and the ground truth, respectively; $p_{k}^{\left(t\right)}$ and ${\tilde{p}}_{k}^{\left(t\right)}$ represent the proportion of cell type *C_t_* in spot *s_k_*, respectively; *μ_t_* and ${\tilde{\mu }}_{t}$ are the average proportion values of cell type *C_t_* in the predicted result and the ground truth, respectively; and *σ_t_* and ${\tilde{\sigma }}_{t}$ are the standard deviations of the spatial cell-type proportion of cell type *C_t_* in the predicted result and the ground truth, respectively. For one cell type, a higher PCC value indicates better prediction accuracy.

2. SSIM. We first scaled the proportion value as follows, thus obtaining the scaled proportion vectors $p^{\prime \left(t\right)}$ and ${\tilde{p}}^{\prime \left(t\right)}$, so that the proportion value of each cell type was between 0 and 1:

$${p^{\prime }}_{k}^{\left(t\right)}=\frac{p_{k}^{\left(t\right)}}{\text{max}\left(\left\{p_{1}^{\left(t\right)},\ldots ,p_{n_{2}}^{\left(t\right)}\right\}\right)}$$ (11)

Then, we used the scaled proportion and the following equation to calculate the SSIM value between the predicted proportion and the ground truth of cell type *C_t_*:

$$\text{SSIM}\left(p^{\left(t\right)},{\tilde{p}}^{\left(t\right)}\right)=\frac{\left(2\mu_{t}^{\prime }{\tilde{\mu }}_{t}^{\prime }+\alpha^{2}\right)\left[2\text{cov}\left(p^{\prime \left(t\right)},{\tilde{p}}^{\prime \left(t\right)}\right)+\beta^{2}\right]}{\left[{\left(\mu_{t}^{\prime }\right)}^{2}+{\left({\tilde{\mu }}_{t}^{\prime }\right)}^{2}+\alpha^{2}\right]\left[{\left(\sigma_{t}^{\prime }\right)}^{2}+{\left({\tilde{\sigma }}_{t}^{\prime }\right)}^{2}+\beta^{2}\right]}$$ (12)

where the definitions of $\mu_{t}^{\prime }$, ${\tilde{\mu }}_{t}^{\prime }$, $\sigma_{t}^{\prime }$, and ${\tilde{\sigma }}_{t}^{\prime }$ are similar to those used when calculating the PCC value (but for scaled proportion); *α* and *β* are 0.01 and 0.03, respectively; and $\text{cov}\left(p^{\prime \left(t\right)},{\tilde{p}}^{\prime \left(t\right)}\right)$ is the covariance between the scaled proportion vector of cell type *C_t_* in the predicted result (that is, $p^{\prime \left(t\right)}$) and that of the ground truth (that is, ${\tilde{p}}^{\prime \left(t\right)}$). For one cell type, a higher SSIM value indicates better prediction accuracy.

3. RMSE. We first calculated the *z*-score of the spatial proportion of cell type *C_t_* for all spots and then evaluated the RMSE as follows:

$$\text{RMSE}\left(p^{\left(t\right)},{\tilde{p}}^{\left(t\right)}\right)=\sqrt{\frac{1}{n_{2}}\sum_{k=1}^{n_{2}}{\left(r_{k}^{\left(t\right)}-{\tilde{r}}_{k}^{\;\left(t\right)}\right)}^{2}}$$ (13)

where $r_{k}^{\left(t\right)}$ and ${\tilde{r}}_{k}^{\;\left(t\right)}$ are the *z*-score of the spatial proportion of cell type *C_t_* in spot *s_k_* in the predicted result and the ground truth, respectively. For one cell type, a lower RMSE value indicates better prediction accuracy.

4. JSD. JSD uses relative information entropy (that is, KL divergence) to determine the difference between 2 distributions. We first calculated the spatial distribution probability of cell type *C_t_* as follows:

$$P_{k}^{\left(t\right)}=\frac{p_{k}^{\left(t\right)}}{\sum_{j=1}^{n_{2}}p_{j}^{\left(t\right)}}$$ (14)

where $P_{k}^{\left(t\right)}$ is the spatial distribution probability of cell type *C_t_* in spot *s_k_*. The KL divergence is defined as:

$$\text{KL}\left(P^{\left(t\right)}\|{\tilde{P}}^{\left(t\right)}\right)=\sum_{k=1}^{n_{2}}P_{k}^{\left(t\right)}\cdot \text{log}\frac{P_{k}^{\left(t\right)}}{{\tilde{P}}_{k}^{\left(t\right)}}$$ (15)

where ***P***^(*t*)^ and ${\tilde{P}}^{\left(t\right)}$ are the spatial distribution probability vectors of cell type *C_t_* in the predicted result and the ground truth, respectively, and $P_{k}^{\left(t\right)}$ and ${\tilde{P}}_{k}^{\left(t\right)}$ are the predicted and real probabilities of cell type *C_t_* in spot *s_k_*, respectively. The JSD can thus be defined as:

$$\begin{array}{c} \text{JSD}\left(p^{\left(t\right)},{\tilde{p}}^{\left(t\right)}\right)=\\ \frac{1}{2}\text{KL}\left(P^{\left(t\right)}\|\frac{P^{\left(t\right)}+{\tilde{P}}^{\left(t\right)}}{2}\right)+\frac{1}{2}\text{KL}\left({\tilde{P}}^{\left(t\right)}\|\frac{P^{\left(t\right)}+{\tilde{P}}^{\left(t\right)}}{2}\right) \end{array}$$ (16)

For one cell type, a lower JSD value indicates better prediction accuracy.

5. APS. An APS was defined by aggregating over PCCs, SSIMs, RMSEs, and JSDs. A higher APS indicates better overall performance. Specifically, for a cell type *C_t_* in one dataset, APS represents the average performance level of PCC, SSIM, RMSE, and JSD of an algorithm:

$$\text{APS}=\frac{1}{4}\left({\text{PL}}_{\text{PCC}}+{\text{PL}}_{\text{SSIM}}+{\text{PL}}_{\text{RMSE}}+{\text{PL}}_{\text{JSD}}\right)$$ (17)

where PL_PCC_, PL_SSIM_, PL_RMSE_ and PL_JSD_ are the performance levels of PCC, SSIM, RMSE, and JSD for the algorithm on cell type *C_t_*, respectively. The method with the highest PCC/SSIM value will have PL_PCC/SSIM_ = *Q*, and the method with the lowest PCC/SSIM value will have PL_PCC/SSIM_ = 1, where *Q* represents the number of all the algorithms for comparison. The method with the highest RMSE/JSD value will have PL_RMSE/JSD_ = 1, and the method with the lowest RMSE/JSD value will have PL_RMSE/JSD_ = *Q*. For cell type *C_t_*, the method with the highest APS value has the best performance among the integration methods.

### Validation for attribution maps of soybean nodule maturation with PlantPhoneDB

We use PlantPhoneDB, a manually curated pan-plant database of ligand–receptor pairs, for inferring CCC. First, due to the current absence of ligand–receptor interactions specific to soybean in PlantPhoneDB, the g:Profiler web tool (http://biit.cs.ut.ee/gprofiler/gost) leveraged its built-in cross-species homology information, with *Arabidopsis thaliana* set as the target species, to perform homologous gene name conversion for soybean genes. The expression of soybean genes is then used as the transformed expression values for their Arabidopsis homologs. In cases where multiple soybean genes mapped to the same Arabidopsis gene, we averaged the expression values of those soybean genes as the expression data of the corresponding Arabidopsis gene, generating an Arabidopsis gene expression matrix with soybean cell labels. Finally, we inferred CCC for soybean using PlantPhoneDB, incorporating its built-in ligand–receptor interactions specific to *Arabidopsis thaliana* along with the above gene expression matrix. This approach served to validate the results of the attribution-based dynamic gradient field obtained by eMCI from a biological perspective. The homologous gene matching information between soybean and *Arabidopsis thaliana* is provided in Table S10.

### Transfer between cell types and clusters in zebrafish melanomas

To validate the stability of eMCI across different zebrafish melanoma tissue sections, we transferred the clusters of different samples with cell-type annotations provided by the original study. Specifically, for each cluster in samples E and F, we assigned the cell type that had the highest proportion within the cluster as the substitute cell type for that cluster. Subsequently, we found that eMCI could effectively identify the spatial distribution of tumor cells even when using single-cell data from different samples. This demonstrates the efficacy and robustness of eMCI in exploring the tumor microenvironment.

### Statistics analysis

To further statistically characterize the relevance of the cell-type-specific attribution map and potential molecular features, we carried out the hypergeometric test to explore whether the spatial expression distribution of a ligand/receptor is significantly enriched by the high-attribution region, inspired by multimodal intersection analysis [36]. Specifically, we denote the number of the background spots (i.e., all the spots covered by the tissue in the dataset) as *M*, the number of ligand/receptor-expressed spots as *N*, the number of high-attribution spots (with top 20% attribution values) as *H*, and the number of the common spots shared by the ligand/receptor-expressed spots and high-attribution spots as *C*. Then, under the null hypothesis that the distribution of high-attribution spots is independent of the distribution of ligand/receptor-expressed spots, the probability of getting *C* common spots is as follows:

PC;M,N,H=NCM−NH−CMH (18)

The *P* value is defined as follows:

$$P=\sum_{k=C}^{\text{min}\left(H,N\right)}P\left(k;M,N,H\right)$$ (19)

The cell-type-specific attribution map is considered to be significantly related to the ligand/receptor-expressed map when *P* < 0.05.

### ICC index for quantifying the cell-type-specific attribution maps

It is obvious that multiple correlation metrics can comprehensively quantify the relationship between different variables. However, using just one or two of these metrics would limit the ability of the model to fully capture the complex relationships between the variables. Therefore, we propose ICC to analyze the colocalization of cell-type-specific information in the attribution maps with various correlation measurements, including MI for measuring the shared information between 2 maps and revealing nonlinear relationship [48], cosine similarity for assessing the similarity between the spatial patterns based on the angle between 2 variables [51], and PCC for evaluating their linear correlation [13] in this study. Given 2 random attribution matrices ${\bar{A}}^{\left(m\right)}$ and ${\bar{A}}^{\left(n\right)}\in \left\{{\bar{A}}^{\left(1\right)},\ldots ,\;{\bar{A}}^{\left(q\right)}\;\right\}$, the ICC was calculated using the following equations:

ICC=MIvecA¯mvecA¯n+SCvecA¯mvecA¯n+PCCvecA¯mvecA¯n3 (20)

with

$$S_{C}\left[\text{vec}\left({\bar{A}}^{\left(m\right)}\right),\;\text{vec}\left({\bar{A}}^{\left(n\right)}\right)\right]=\frac{\sum_{i=1}^{T}{\bar{A}}_{i}^{\left(m\right)}{\bar{A}}_{i}^{\left(n\right)}}{\sqrt{\sum_{i=1}^{T}{\left({\bar{A}}_{i}^{\left(m\right)}\right)}^{2}}\cdot \sqrt{\sum_{i=1}^{T}{\left({\bar{A}}_{i}^{\left(n\right)}\right)}^{2}}}$$ (21)

where ${\bar{A}}_{i}^{\left(m\right)}$ and ${\bar{A}}_{i}^{\left(n\right)}$ are the *i*th components in the vectorizations of matrices ${\bar{A}}^{\left(m\right)}$ and ${\bar{A}}^{\left(n\right)}$, denoted as $\text{vec}\left({\bar{A}}^{\left(m\right)}\right)$ and $\text{vec}\left({\bar{A}}^{\left(n\right)}\right)$, respectively, and *T* is the length of $\text{vec}\left({\bar{A}}^{\left(m\right)}\right)$ or $\text{vec}\left({\bar{A}}^{\left(m\right)}\right)$. Furthermore, an ablation study was performed to test the effectiveness of the ICC metrics. The results confirmed that using the combined correlation metrics provides a more accurate and robust measurement of cellular communication patterns, validating the need for all 3 metrics (Fig. S13 and Table S7).

In summary, for each cell, a corresponding correlation image is produced by the aforementioned procedure. After classification, eMCI performs attribution analysis for each cell image and aggregates the images based on cell type to produce cell-type-specific attribution maps. In subsequent analyses of cell communication using ICC metrics, eMCI specifically works with these cell-type images aggregating individual cell attribution maps. This approach enables the capture of cell-type-level communication patterns.

### Validation for attribution maps of human embryonic lung with CellChat v2

To biologically validate the attribution-based results, we utilized CellChat v2 to perform CCC analysis from single-cell data. Specifically, we characterized proximal cellular communication networks based on single-cell data and summarized cellular interaction strength across different stages to validate the dynamic trends of ICC values between cell-type-specific attribution results, wherein the cell labels for scRNA-seq data are directly adopted from the original work [37]. Moreover, we assigned the cell type corresponding to the highest attribution value in each spot as its label. Subsequently, we characterized the spatial cellular communication network based on ICC values to validate distal cellular communication which is not easily recognizable by conventional CCC methods (Fig. 1D).

### ARI

The ARI is used for comparing the clusters based on the deconvolution result (labeled as the predominant cell type in each spot) with the original region labels for spots. We denote the number of spots in the *i*th cluster based on the deconvolution as *λ_i_* (*i* = 1, 2, ⋯, *k*_1_, *k*_1_ represents the total number of clusters), the number of spots with the *j*th region label as *τ_j_* (*j* = 1, 2, ⋯, *k*_2_, *k*_2_ represents the total number of region labels), and the number of spots with both the *i*th cluster and *j*th region label as *ω_ij_*. The ARI is calculated as follows:

ARI=∑i=1k1∑j=1k2ωij2−∑i=1k1λi2∑j=1k2τj2n2212∑i=1k1λi2+∑j=1k2τj2−∑i=1k1λi2∑j=1k2τj2n22 (22)

where *n*_2_ represents the total number of spots. The ARI value ranges from −1 to 1, with higher values indicating a greater correspondence between the spatial distributions of the predominant cell types inferred from our deconvolution method and the true tissue segmentation.

### Functional enrichment analysis for lung development data

In this study, we selected mesenchymal cells as a target cell type. We conducted differential expression analysis by DESeq2 for the count matrices from high-attribution spots (with top 20% attribution values) and other spots in an attribution map. After obtaining DEGs from the above procedure, with *P* − *adjust* < 0.05, we carried out the GO and Kyoto Encyclopedia of Genes and Genomes functional enrichment analysis by the clusterProfiler package [52].

### Datasets

#### Mouse visual cortex dataset

The annotated scRNA-seq reference and gridded ST data of mouse visual cortex were downloaded from https://github.com/QuKunLab/SpatialBenchmarking/tree/main/FigureData/Figure4/Dataset10_STARmap, including 1,549 cells that correspond to 15 cell types and 189 square pseudo-spots.

#### Zebrafish melanoma datasets

The ST data from 3 samples (A, B, and C, with 2,425, 2,179, and 2,677 spots, respectively) and scRNA-seq data from 2 samples (E and F, with 1,911 and 1,085 cells, respectively) of zebrafish melanoma were available from the Gene Expression Omnibus (GEO) database under accession code GSE159709.

#### Soybean datasets

For soybean, the snRNA-seq data, including 8,229 cells from 12 dpi and 12,004 cells from 21 dpi, along with ST data from 4 tissue slices at 12 and 21 dpi with 2,229, 1,871, 1,593, and 1,981 spots per slice, was available from the Open Archive for Miscellaneous Data under accession code OMIX002290.

#### Lung development datasets

The scRNA-seq data for human embryonic lung utilized in this study consisted of samples across 7 developmental stages, with cell counts ranging from 3,781 to 15,845, available from GEO (GSE215895). Similarly, the ST data used in this study comprised samples across 7 developmental stages, with spot counts ranging from 355 to 4,400, accessible from GEO (GSE215897).

#### Human fetal pancreas datasets

The scRNA-seq data of human fetal pancreases at 12 and 20 PCW, with 4,321 and 2,122 cells, respectively, used for this study, were available on GEO with accession numbers GSE197064. The paired ST data at 12 and 20 PCW, with 294 and 2,126 spots, respectively, were available on GEO with accession number GSE197317.

#### PDAC dataset

The scRNA-seq data of pancreatic ductal adenocarcinoma containing 1,926 cells and the ST data containing 428 spots can be accessed at GEO under accession number GSE111672.

#### BRCA dataset

The scRNA-seq data of BRCA, originally consisting of 100,064 cells, was publicly available from GEO under accession code GSE176078, while the ST data of BRCA, containing 4,727 spots, was publicly available from the 10x Genomics website (https://support.10xgenomics.com/spatialgene-expression/datasets).

The details for the paired single-cell and ST datasets from different tissues and species are provided in Table S1.

## Data Availability

The processed data used for this study have been deposited on the Zenodo repository (https://zenodo.org/records/12176330). All the scripts in this study are available in the GitHub repository: https://github.com/Hongrenhao/eMCI.

## Supplementary Materials

Supplementary 1 Section S1. Figs. S1 to S26 Section S2. Tables S1 to S10 Section S3. Note S1 Supplementary References
