# Supplementary material for: eMCI: An Explainable Multimodal Correlation Integration Model for Unveiling Spatial Transcriptomics and Intercellular Signaling
Source: Research (Wash D C). 2024 Nov 1;7:0522. doi: 10.34133/research.0522 (PMC11528068; doi:10.34133/research.0522)
Supplement: Supplementary 1 — Section S1. Figs. S1 to S26 Section S2. Tables S1 to S10 Section S3. Note S1 Supplementary References [file research.0522.f1.zip › Research_eMCI_SI_finalversion2.docx]

**Supplementary Information of**

**“eMCI: an Explainable Multimodal Correlation Integration Model for Unveiling Spatial Transcriptomics and Intercellular Signaling”**

Renhao Hong^1#^, Yuyan Tong^1#^, Hui Tang^2*^, Tao Zeng^3,4*^, Rui Liu^1*^

^1^ School of Mathematics, South China University of Technology, Guangzhou, 510640, China.

^2^ School of Mathematics and Big Data, Foshan University, Foshan 528000, China.

^3^ Guangzhou Laboratory, Guangzhou, China.

^4^ GMU-GIBH Joint School of Life Sciences, The Guangdong-Hong Kong-Macau Joint Laboratory for Cell Fate Regulation and Diseases, Guangzhou Laboratory, Guangzhou Medical University.

^#^ These authors contributed equally to this work.

^*^ Address correspondence to: Hui Tang; tanghui@fosu.edu.cn; Tao Zeng; zeng_tao@gzlab.ac.cn or zengtao@sibs.ac.cn; Rui Liu; scliurui@scut.edu.cn.

Content

[**Section S1. The supplementary figures** 3](#_Toc177988765)

[Fig. S1. Performance evaluations of eMCI on multi-class classification task 3](#_Toc177988766)

[Fig. S2. Confusion matrices of eMCI for the real datasets 4](#_Toc177988767)

[Fig. S3. Confusion matrices of eMCI for PDAC 5](#_Toc177988768)

[Fig. S4. Comparison of classification accuracy of the eMCI method with two other methods across various cell types 6](#_Toc177988769)

[Fig. S5. A schematic illustration of eMCI for multi-label classification 6](#_Toc177988770)

[Fig. S6. A schematic illustration of the eMCI framework without fusing spatial transcriptomics data for multi-label classification 7](#_Toc177988771)

[Fig. S7. Deconvolution performance of eMCI for other cell types in the mouse cortex dataset 8](#_Toc177988772)

[Fig. S8. Dimension reduction by UMAP for zebrafish melanomas 9](#_Toc177988773)

[Fig. S9. Deconvolution based on eMCI for all the paired datasets of zebrafish melanoma 10](#_Toc177988774)

[Fig. S10. KEGG enrichment analysis for zebrafish melanoma 11](#_Toc177988775)

[Fig. S11. GO enrichment analysis for zebrafish melanoma 12](#_Toc177988776)

[Fig. S12. Analysis for the eMCI result of other tissue slices in soybean nodule maturation 13](#_Toc177988777)

[Fig. S13. An ablation study for validating the effectiveness of the ICC metrics 14](#_Toc177988778)

[Fig. S14. The average percentage of different cell types in diverse regions of each soybean nodule’s replicate section 14](#_Toc177988779)

[Fig. S15. Overview of ligand–receptor interactions between different cell types in soybean nodule maturation 15](#_Toc177988780)

[Fig. S16. Cellular deconvolution for all the cell types in the human embryonic lung 16](#_Toc177988781)

[Fig. S17. Attribution analysis for all the cell types in the human embryonic lung 17](#_Toc177988782)

[Fig. S18. Quantification for similarity between cell type-specific attribution maps in the human embryonic lung at different stages 18](#_Toc177988783)

[Fig. S19. Correlation analysis for ICC matrices at different stages 18](#_Toc177988784)

[Fig. S20. Comparison of dynamic trends between cellular interaction strengths inferred by CellChat v2 and ICC values 19](#_Toc177988785)

[Fig. S21. Comparison of eMCI performance on the raw count data and batch-corrected data 20](#_Toc177988786)

[Fig. S22. Benchmarking eMCI’s sensitivity to sequencing quality in ST and scRNA-seq datasets 20](#_Toc177988787)

[Fig. S23. Benchmarking eMCI’s sensitivity to sequencing quality in ST and scRNA-seq datasets 21](#_Toc177988788)

[Fig. S24. Comparison of individual correlation metrics with the combination of eMCI in the cell-type deconvolution task 21](#_Toc177988789)

[Fig. S25. Comparison of the model’s performance in the cell-type classification task using multiple correlations versus individual metrics in eMCI 22](#_Toc177988790)

[Fig. S26. An illustration for the ResNet50 architecture 22](#_Toc177988791)

[**Section S2. The supplementary tables** 23](#_Toc177988792)

[Table S1. Detailed information for the applied datasets 23](#_Toc177988793)

[Table S1.1. Detailed information for the applied single-cell RNA-seq data 23](#_Toc177988794)

[Table S1.2. Detailed information for the applied spatial transcriptomics data 26](#_Toc177988795)

[Table S2. Classification accuracy of the eMCI framework under different conditions for different datasets 26](#_Toc177988796)

[Table S3. Comparison of eMCI deconvolution performance with other methods using different evaluation metrics on simulated data 26](#_Toc177988797)

[Table S4. Matching relationship between clusters and cell type annotations in zebrafish melanomas 28](#_Toc177988798)

[Table S5. GO enrichment analysis for zebrafish melanoma based on the cell-type deconvolution by Cell2location 28](#_Toc177988799)

[Table S5.1. GO enrichment analysis for the DEGs selected from the paired dataset of samples A and F 28](#_Toc177988800)

[Table S5.2. GO enrichment analysis for the DEGs selected from the paired dataset of samples B and E 29](#_Toc177988801)

[Table S6. Quantification of the overlap between the attribution distribution and the spatial distribution for each cell type 29](#_Toc177988802)

[Table S6.1. The number of intersections between high-attribution spots and high-proportion spots for each cell type 29](#_Toc177988803)

[Table S6.2. The proportion of intersection spots within high-attribution/high-proportion spots for each cell type 30](#_Toc177988804)

[Table S7. The error between the communication strengths inferred by different metrics and that of PlantPhoneDB 30](#_Toc177988805)

[Table S8. Scaled cellular interaction strength by CellChat v2 30](#_Toc177988806)

[Table S9. Comparison of dynamic trends between cellular interaction strengths inferred by CellChat v2 and ICC values 32](#_Toc177988807)

[Table S10. Matching information for homologous genes between soybean and Arabidopsis thaliana 33](#_Toc177988808)

[**Section S3. The Supplementary Notes** 35](#_Toc177988809)

[Supplementary Note S1. Detailed description for the application workflow of eMCI 35](#_Toc177988810)

[**Supplementary references** 37](#_Toc177988811)

# Section S1. The supplementary figures

## Fig. S1. Performance evaluations of eMCI on multi-class classification task


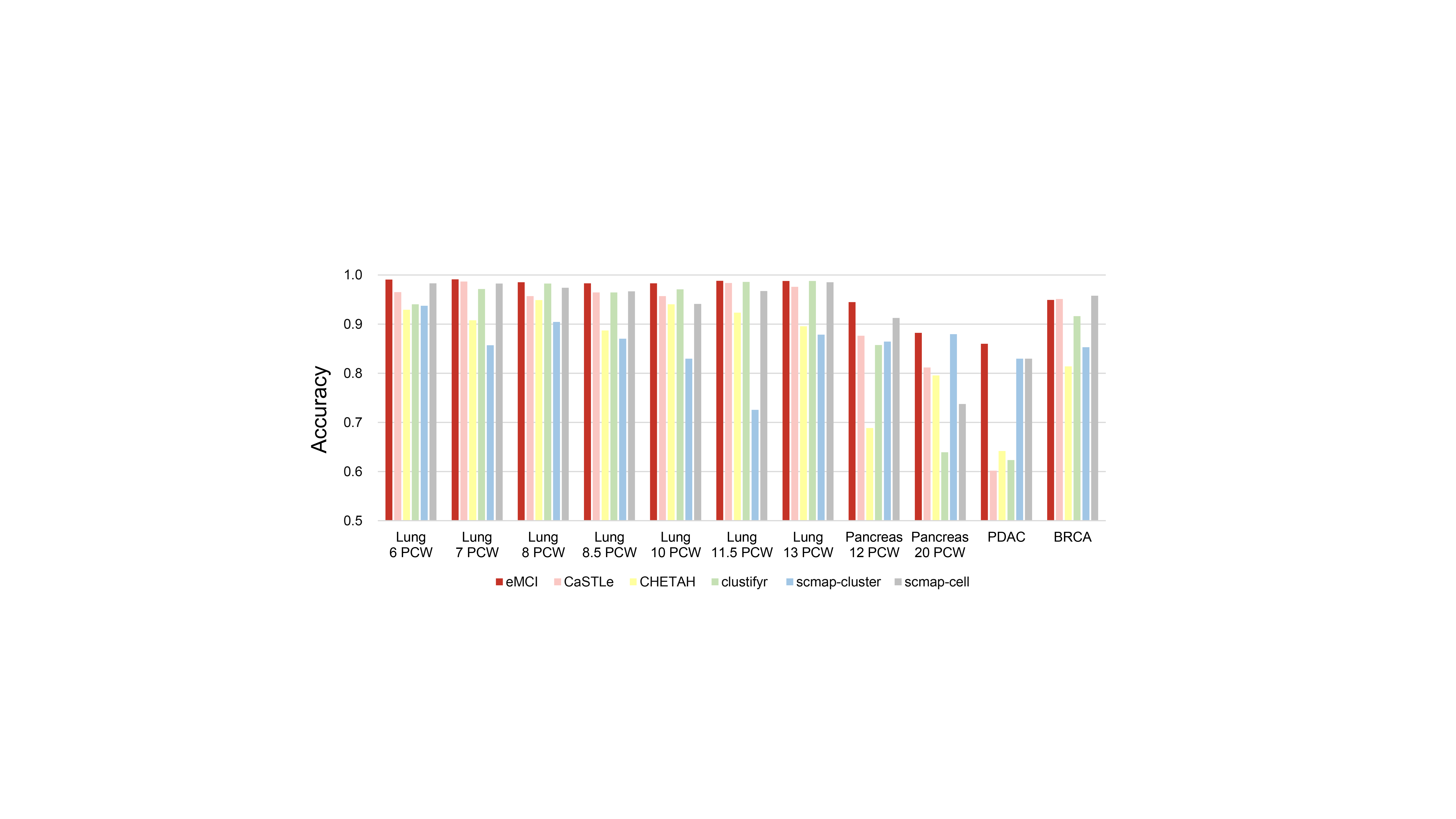


**Figure S1. Performance evaluations of eMCI on multi-class classification tasks.** Comparison of the cell-type classification performance of eMCI in 11 datasets with that of the five other existing methods. eMCI demonstrates similar or better accuracy in cell-type classification compared with other existing methods.

## Fig. S2. Confusion matrices of eMCI for the real datasets


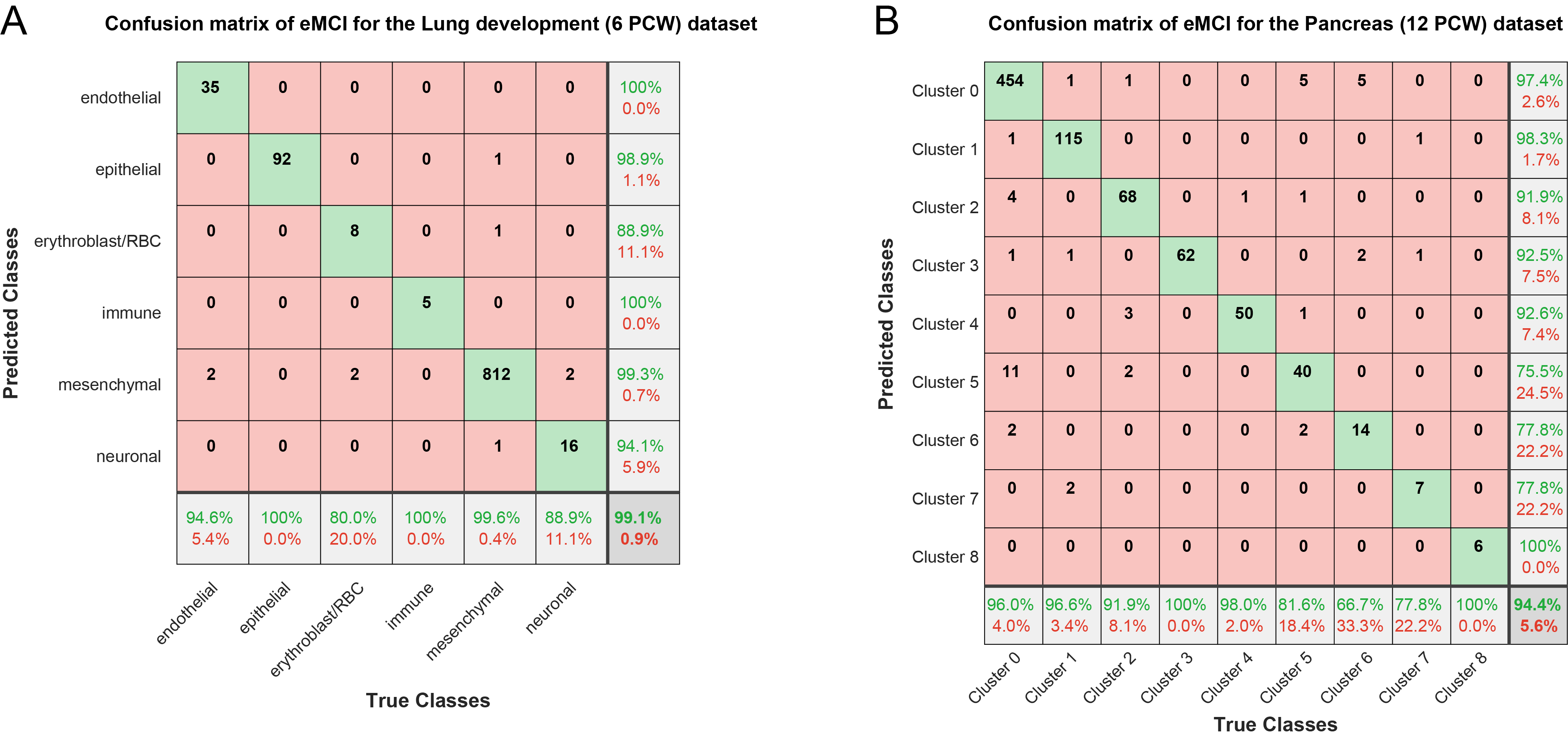


**Figure S2. Confusion matrices of eMCI for the real datasets.** **(A)** Confusion matrix of eMCI for the lung development (6 PCW) dataset. **(B)** Confusion matrix of eMCI for the pancreas (12 PCW) dataset. Overall, eMCI achieved a classification accuracy of over 90% for most cell types in the real datasets, *i.e.*, the lung development (6 PCW) and pancreas (12 PCW) datasets, demonstrating its strong ability to accurately differentiate between cell populations.

## Fig. S3. Confusion matrices of eMCI for PDAC


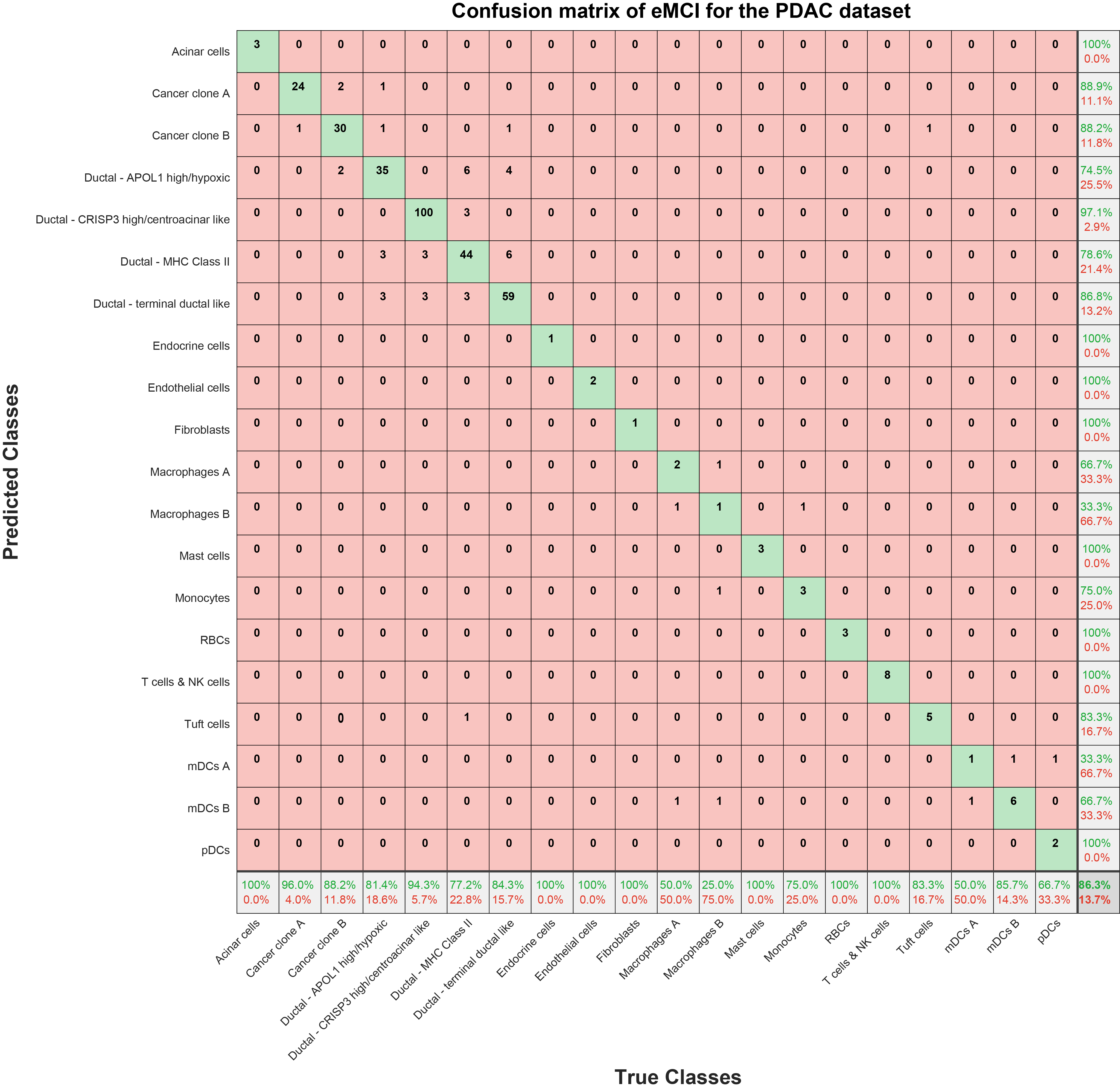


**Figure S3. Confusion matrices of eMCI for the PDAC dataset.** eMCI achieved a classification accuracy of over 80% for most cell types in the PDAC dataset, demonstrating its strong ability to accurately differentiate between cell populations.

## Fig. S4. Comparison of classification accuracy of the eMCI method with two other methods across various cell types


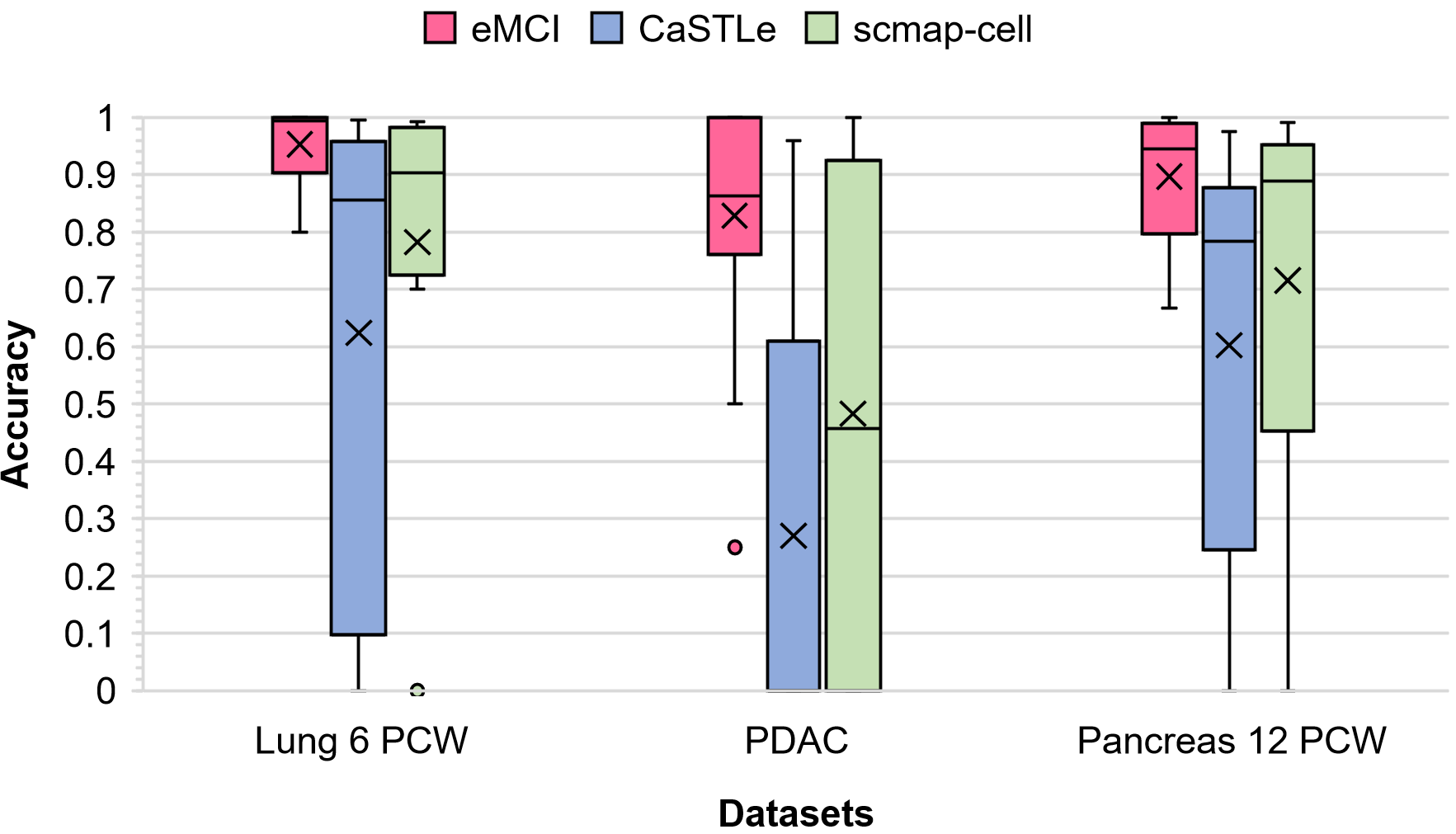


**Figure S4. Comparison of classification accuracy of the eMCI method with two other methods across various cell types.** The box plots show that eMCI achieves higher average accuracy and exhibits less variability compared to the other methods, indicating superior performance and stability. Note: The symbol “$\times$” represents the mean of classification accuracy.

## Fig. S5. A schematic illustration of eMCI for multi-label classification


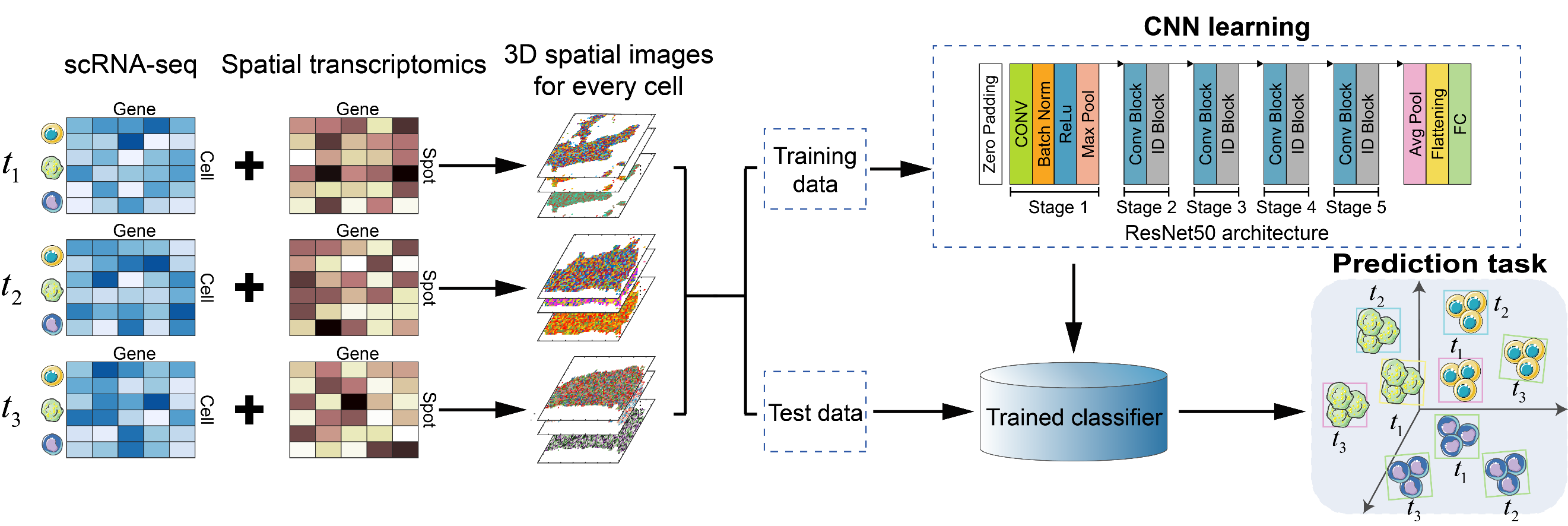


**Figure S5. A schematic illustration of eMCI for multi-label classification.** Based on different cell and spot correlations incorporating the common genes across all time points, we fused single-cell and spatial transcriptomics data across all time points into 3D spatial-image representations, achieving the multi-label classification task of time and cell type using the eMCI framework.

## Fig. S6. A schematic illustration of the eMCI framework without fusing spatial transcriptomics data for multi-label classification


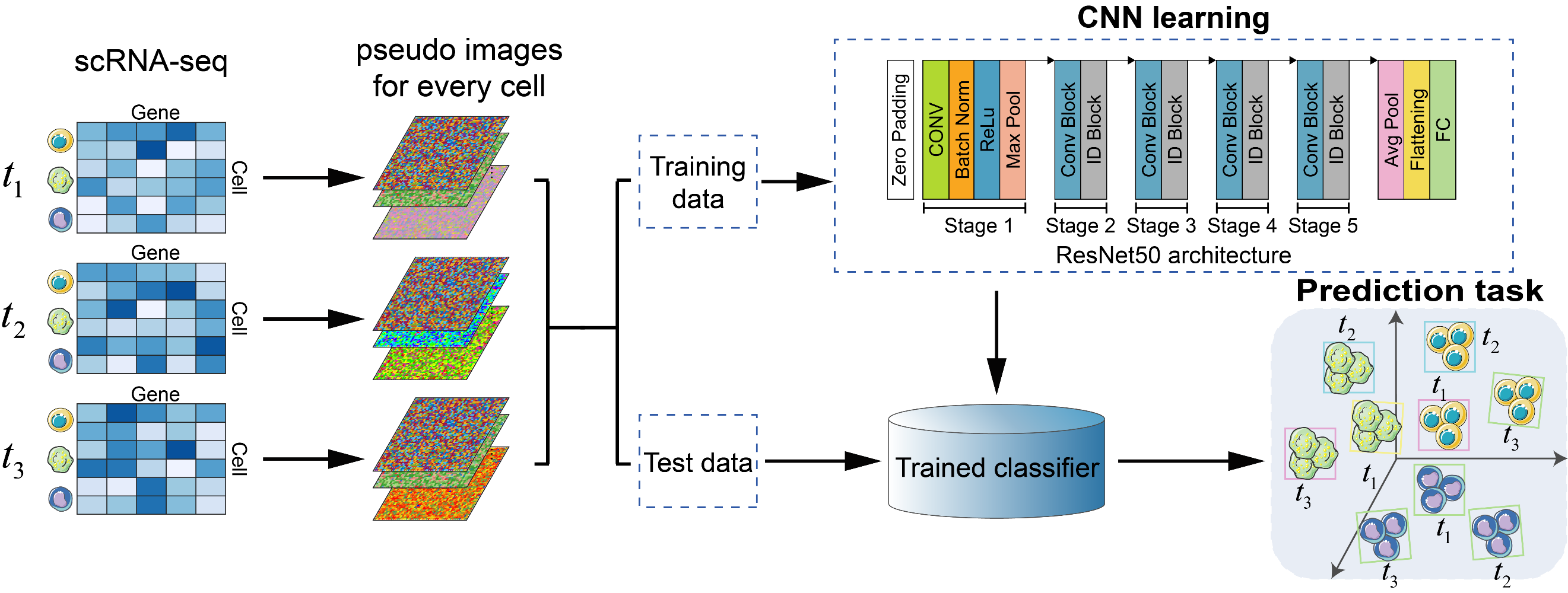


**Figure S6. A schematic illustration of the eMCI framework without fusing spatial transcriptomics data for multi-label classification.** Considering the common genes across all time points, we transformed expression data of each cell across all time points into pseudo-image representations, wherein the expression value of each gene is assigned to the specific position on the pseudo image for a cell, achieving the multi-label classification task of time and cell type using the trained CNN classifier.

## Fig. S7. Deconvolution performance of eMCI for other cell types in the mouse cortex dataset


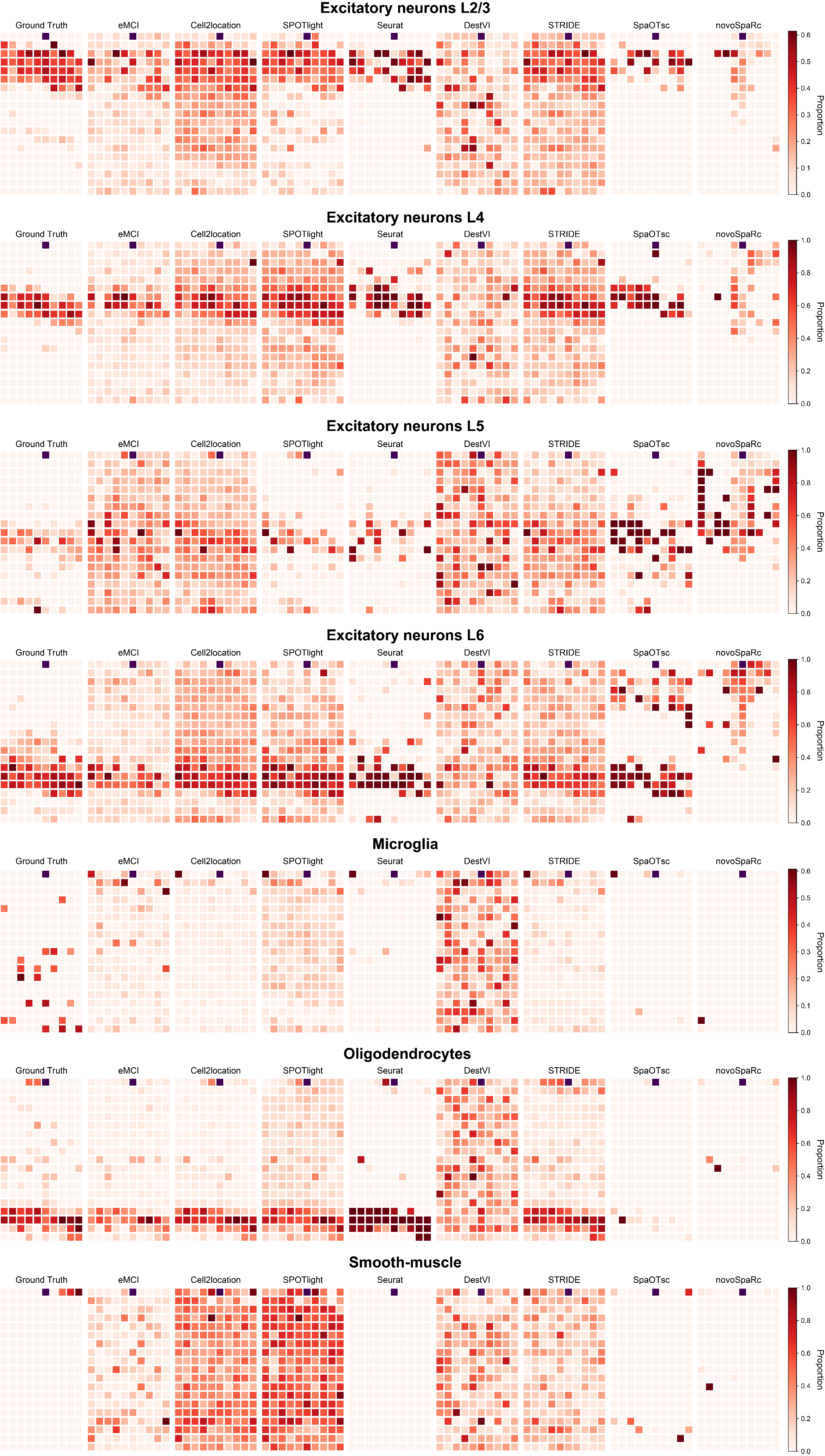


**Figure S7. Deconvolution performance of eMCI for other cell types in the mouse cortex dataset.** Comparison of the cell-type deconvolution performance of eMCI in seven cell types with that of the seven other existing methods. eMCI demonstrates similar performance in cell-type deconvolution compared with other existing methods. Detailed descriptions of other methods are provided in the Methods.

## Fig. S8. Dimension reduction by UMAP for zebrafish melanomas


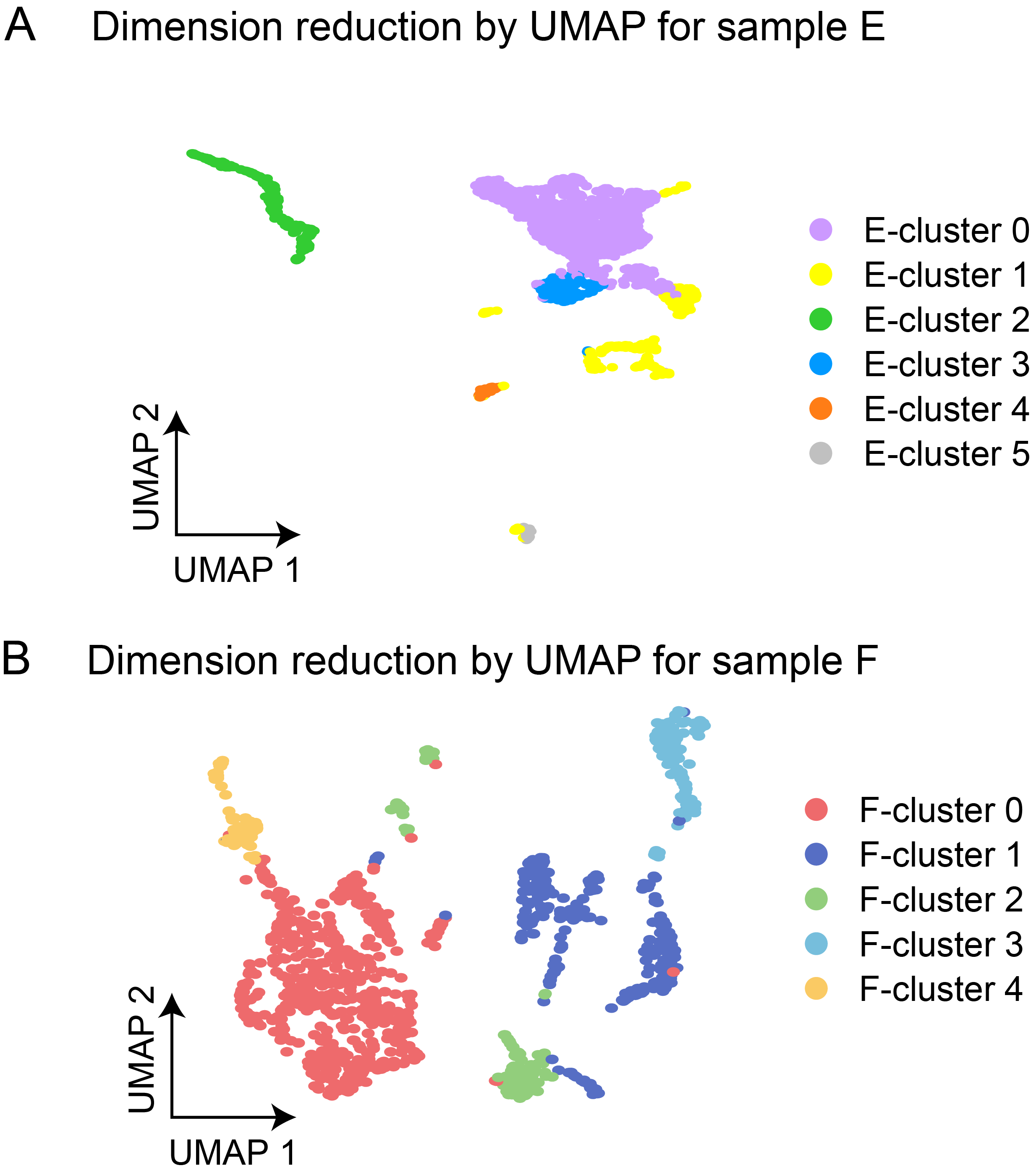


**Figure S8. Dimension reduction by UMAP for zebrafish melanomas.** UMAP embedding of cells from two samples of zebrafish melanomas. (A, B) UMAP projection of 1911 cells from the sample E and 1085 cells from the sample F. Clusters are inferred and colored according to specifically expressed genes.

## Fig. S9. Deconvolution based on eMCI for all the paired datasets of zebrafish melanoma


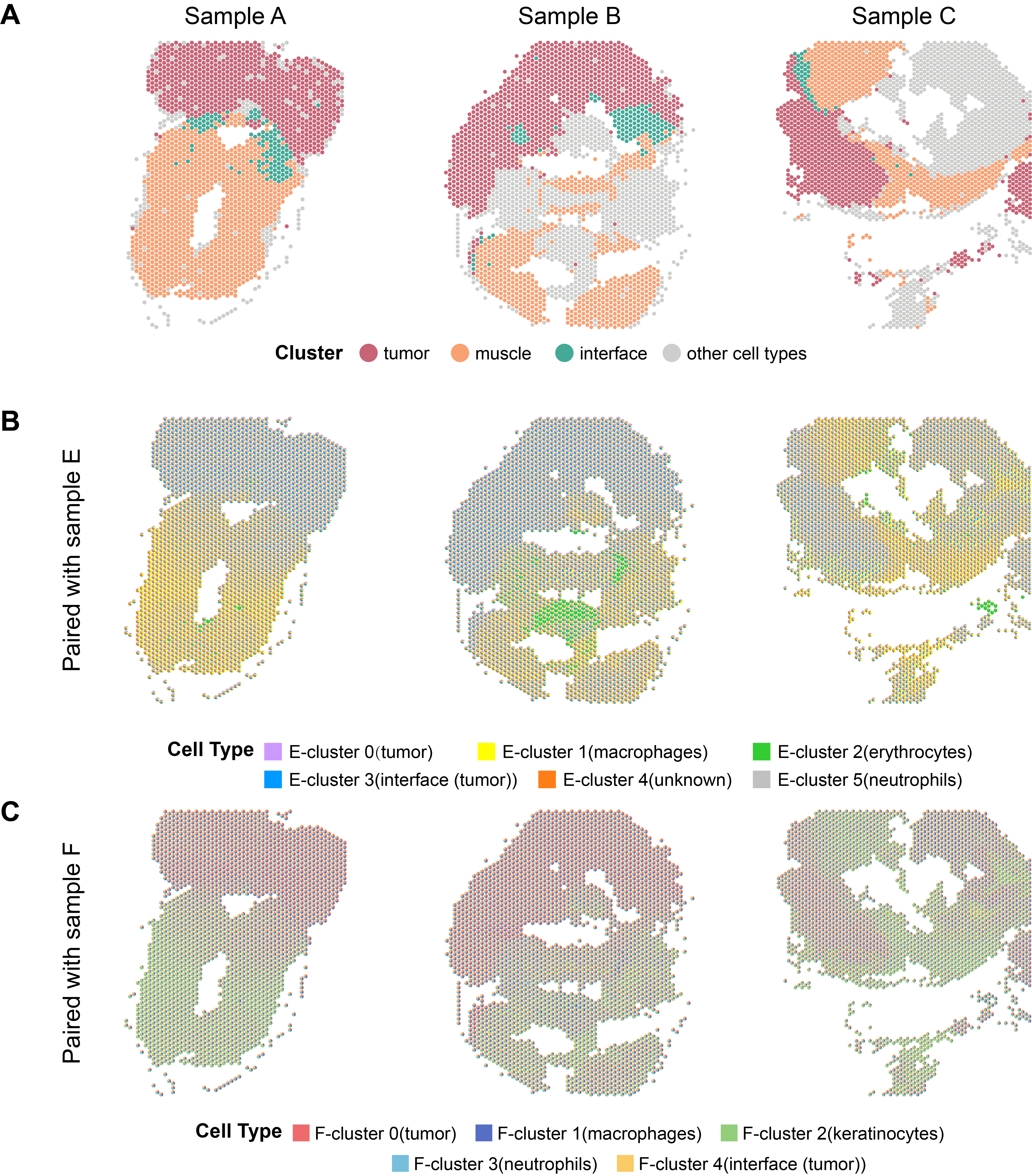


**Figure S9. Deconvolution based on eMCI for all the paired datasets of zebrafish melanoma.** (A) Visium array spots colored by clustering assignments for samples A, B and C in the original literature. (B) Deconvolution performance of eMCI for the paired datasets of the spatial transcriptomics data from three samples (*i.e.*, A, B and C) and scRNA-seq data from sample E. (C) Deconvolution performance of eMCI for the paired datasets of the spatial transcriptomics data from three samples (*i.e.*, A, B and C) and scRNA-seq data from sample F.

## Fig. S10. KEGG enrichment analysis for zebrafish melanoma


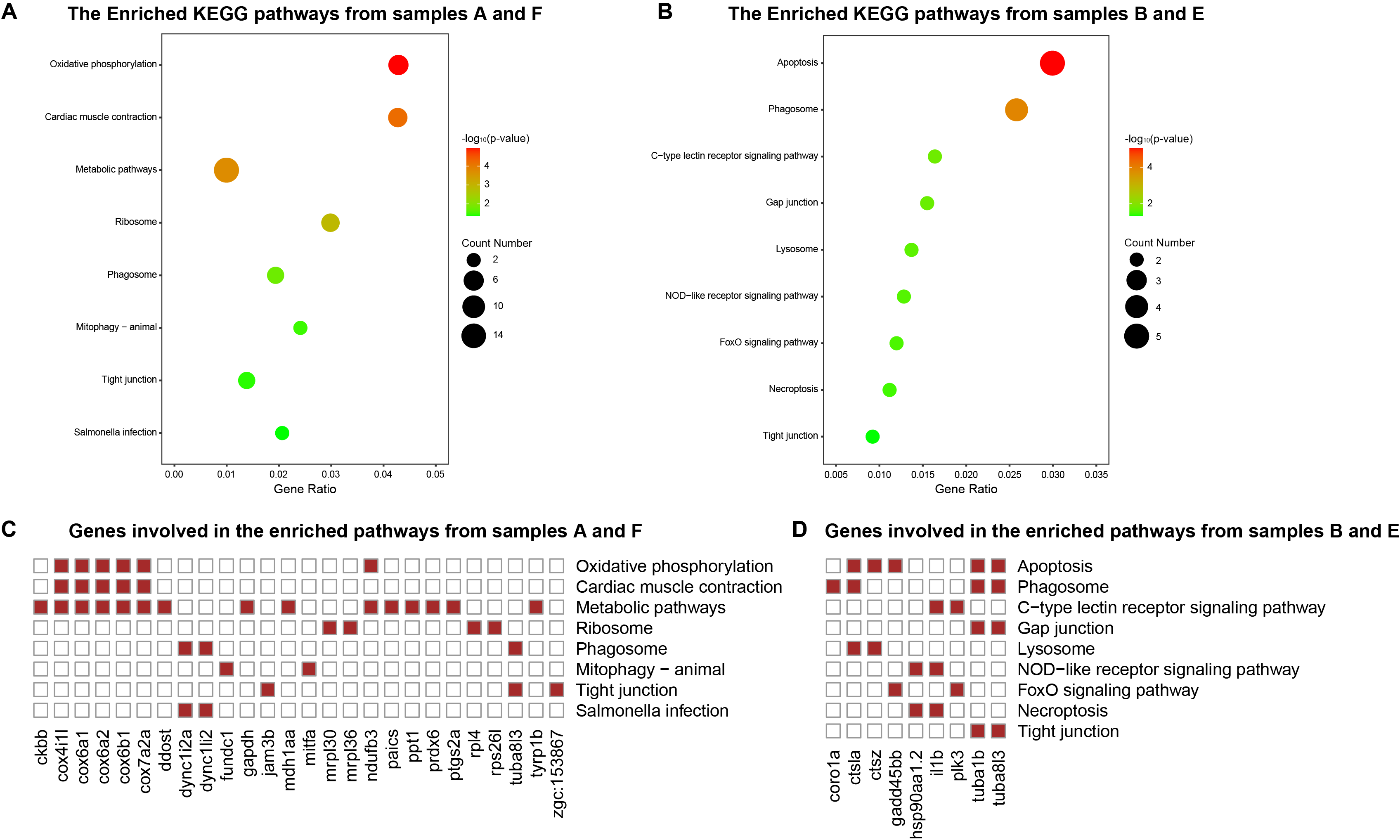


**Figure S10. KEGG enrichment analysis for zebrafish melanoma.** (A, C) KEGG pathway enrichment analysis for the DEGs selected based on the deconvolution results by eMCI for the paired dataset of samples A and F. (B, D) KEGG pathway enrichment analysis for the DEGs selected based on the deconvolution results by eMCI for the paired dataset of samples B and E.

## Fig. S11. GO enrichment analysis for zebrafish melanoma


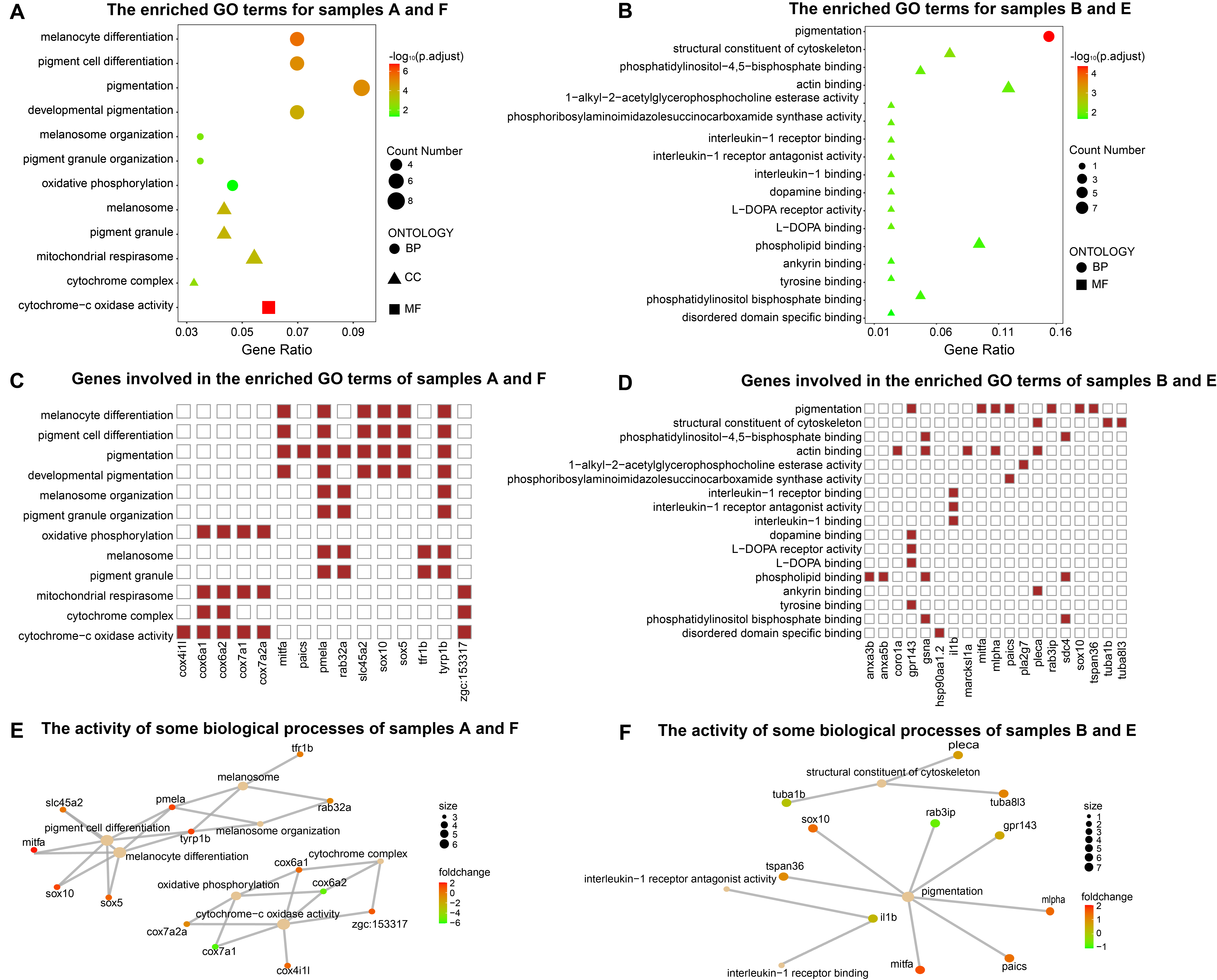


**Figure S11. GO enrichment analysis for zebrafish melanoma.** (A, C and E) Gene ontology enrichment analysis for the DEGs selected based on the deconvolution results by eMCI for the paired dataset of samples A and F. (B, D and F) Gene ontology enrichment analysis for the DEGs selected based on the deconvolution results by eMCI for the paired dataset of samples B and E.

## Fig. S12. Analysis for the eMCI result of other tissue slices in soybean nodule maturation


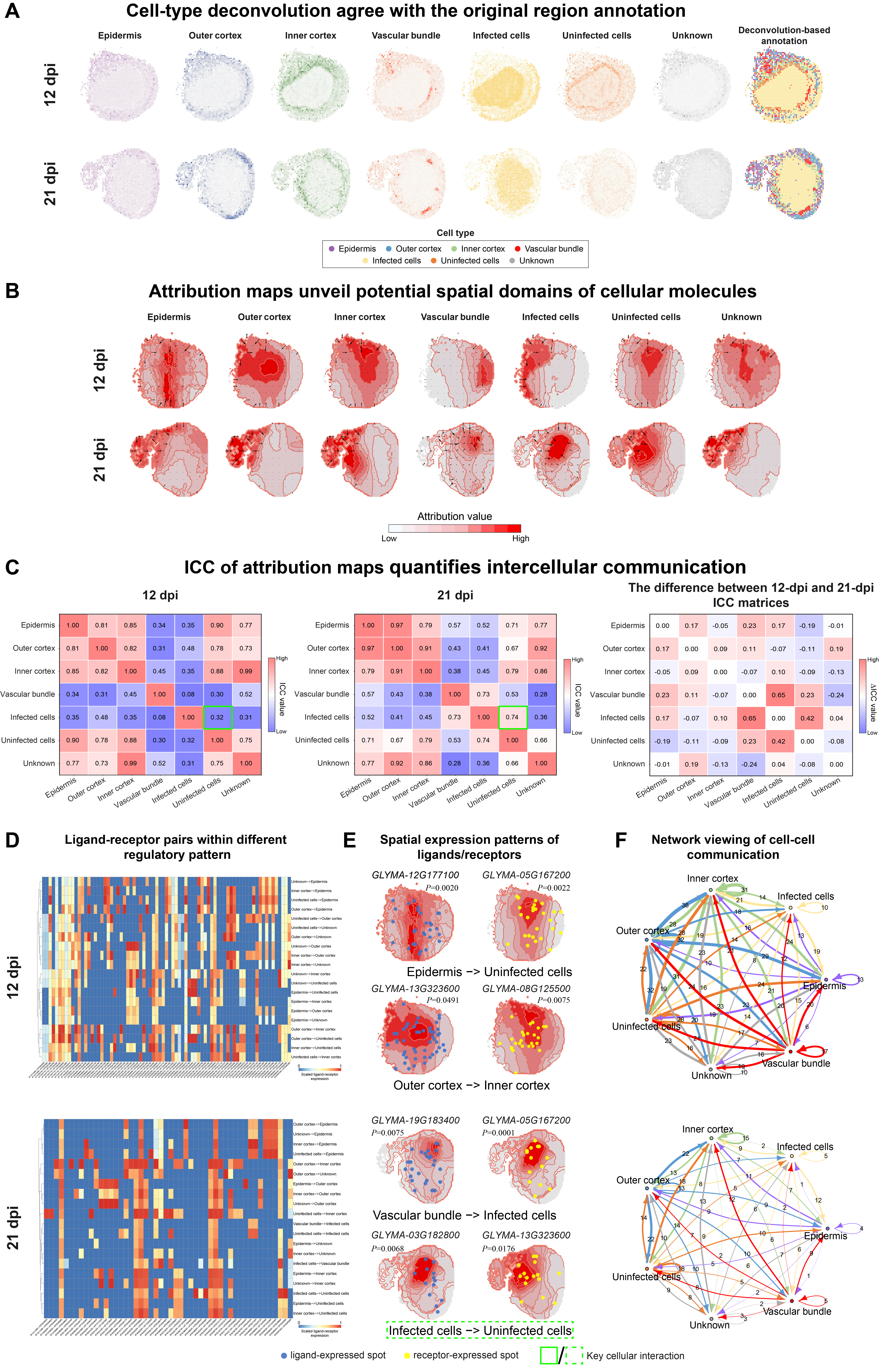


**Figure S12. Analysis for the eMCI result of soybean nodule maturation.** (A) The cell-type deconvolution by eMCI for the 12-dpi and 21-dpi nodules. (B) The cell type-specific attribution maps with dynamic gradient field derived by eMCI for the 12-dpi and 21-dpi nodules. (C) ICC (integrated correlation coefficient) quantifies the similarity of cell type-specific attribution maps at 12 dpi and 21 dpi. PlantPhoneDB validated the attribution-based similarity from a biological perspective. (D) A diverse range of ligand-receptor pairs shows different regulatory patterns. Columns are scaled by max ligand-receptor expression. (E) The spatial expression patterns of certain pairwise ligands and receptors within intercellular interactions are significantly associated with the attribution maps. Blue dots represent expressed spots. (F) Network viewing of cell–cell communication between pairwise cell types.

## Fig. S13. An ablation study for validating the effectiveness of the ICC metrics


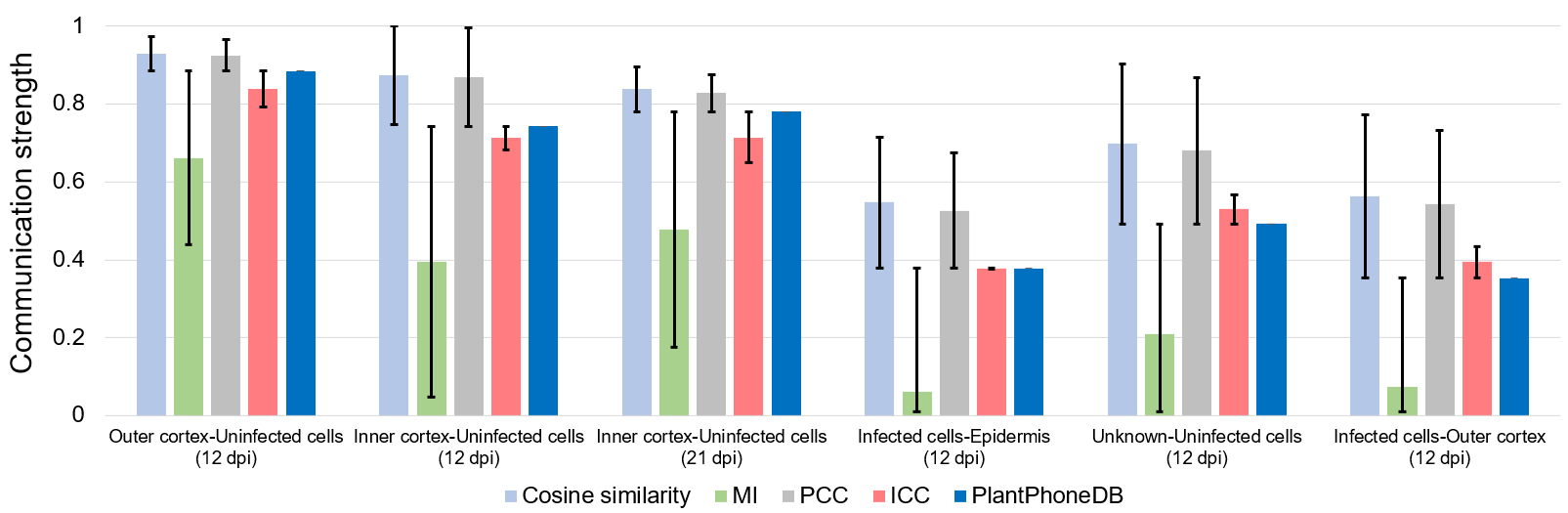


**Figure S13.** **An ablation study for validating the effectiveness of the ICC metrics.** In some key cellular interactions during the soybean nodule maturation, we found that the communication strength indicated by ICC aligned more closely with the results from PlantPhoneDB compared to those obtained using individual correlation metrics.

## Fig. S14. The average percentage of different cell types in diverse regions of each soybean nodule’s replicate section


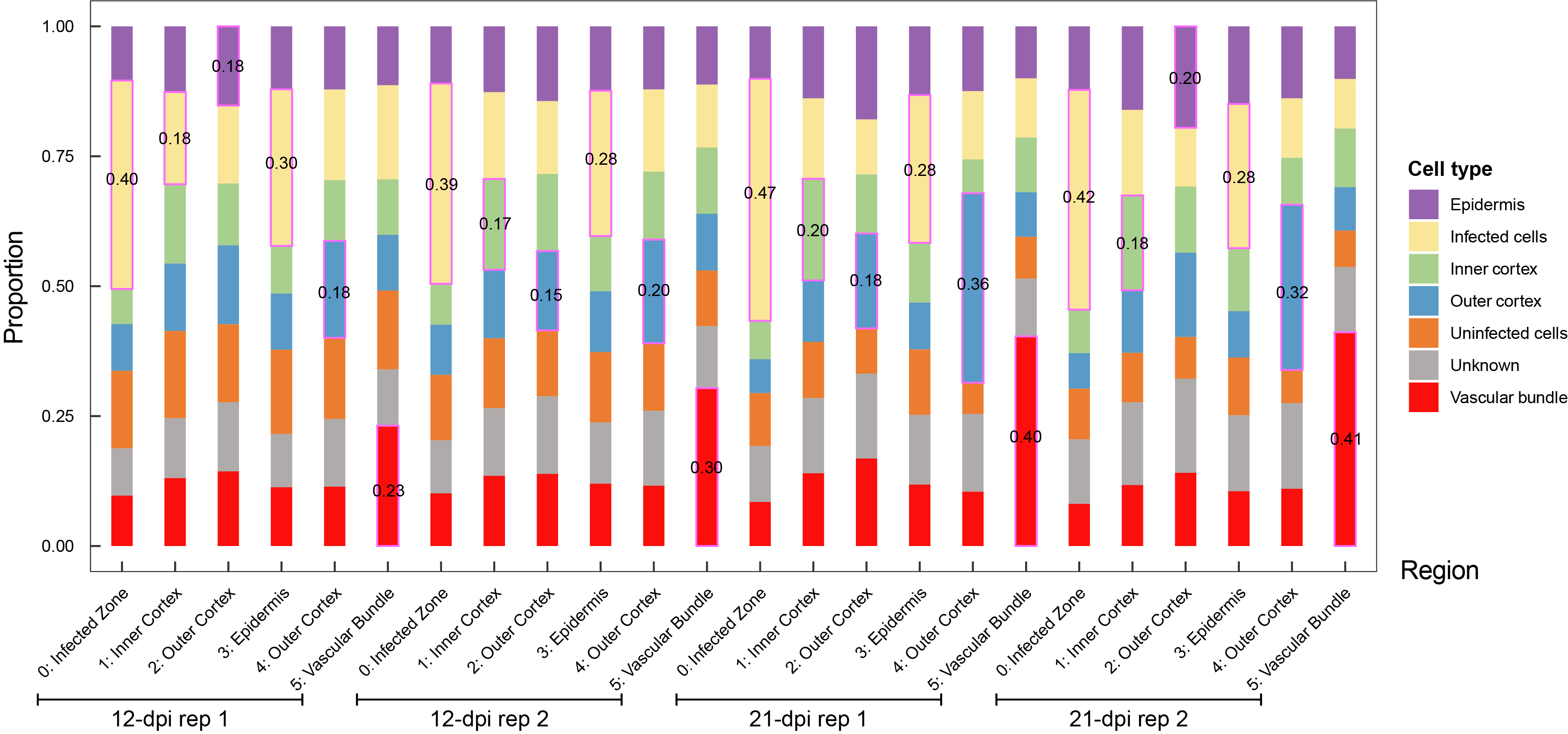


**Figure S14.** **The average percentage of different cell types in diverse regions of each soybean nodule’s** **replicate section.** The bar chart represents the average percentage of different cell types deduced by eMCI in various regions of each replicate section. The region annotation for each spot is determined based on the original literature.

## Fig. S15. Overview of ligand–receptor interactions between different cell types in soybean nodule maturation


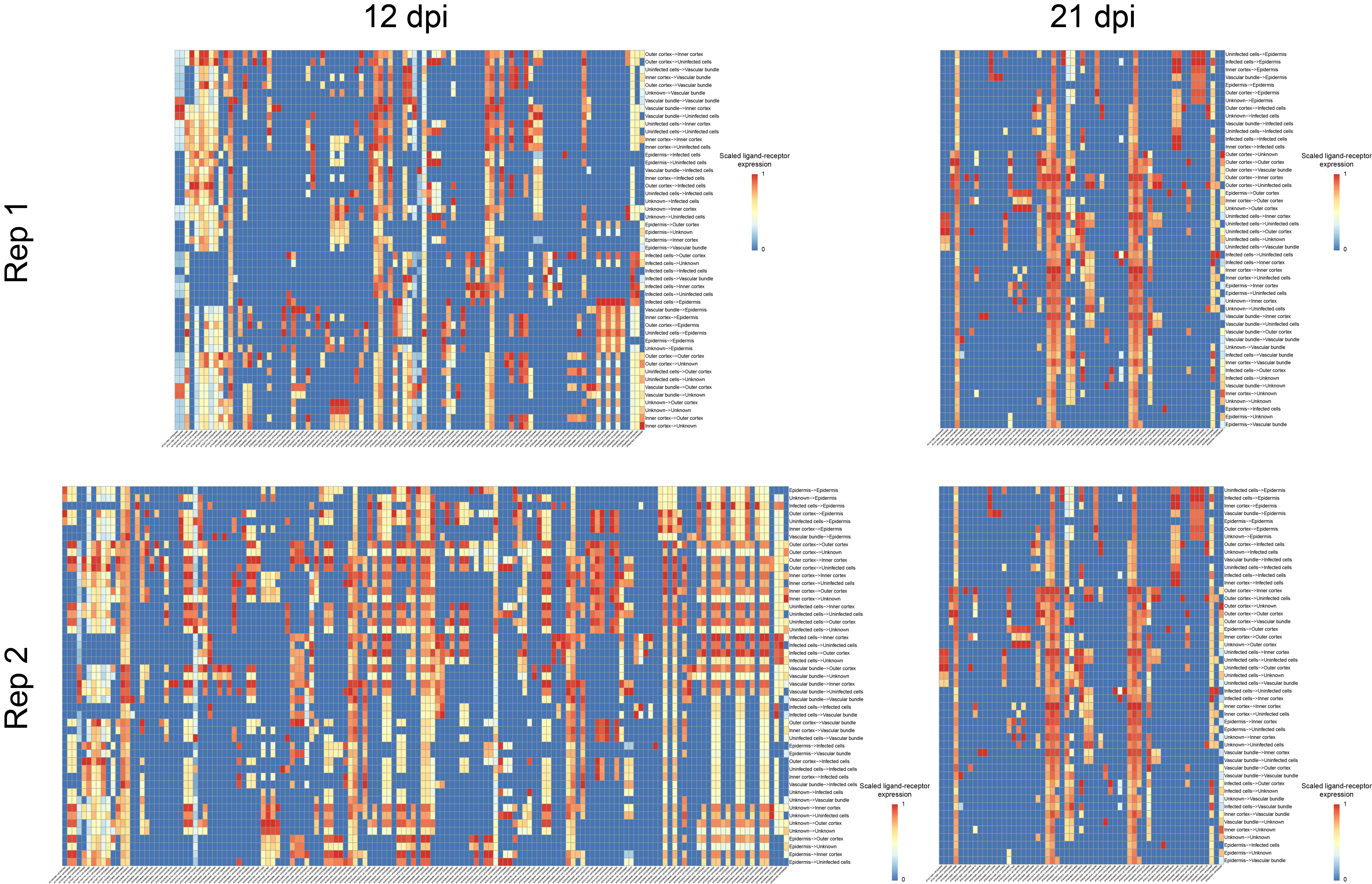


**Figure S15. Overview of ligand–receptor interactions between different cell types in soybean nodule maturation.** X-axis and Y-axis represent ligand–receptor pair and cell–cell pair, respectively. Each grid represents a ligand–receptor pair in a cell–cell interaction, colored by the scaled ligand-receptor expression.

## Fig. S16. Cellular deconvolution for all the cell types in the human embryonic lung


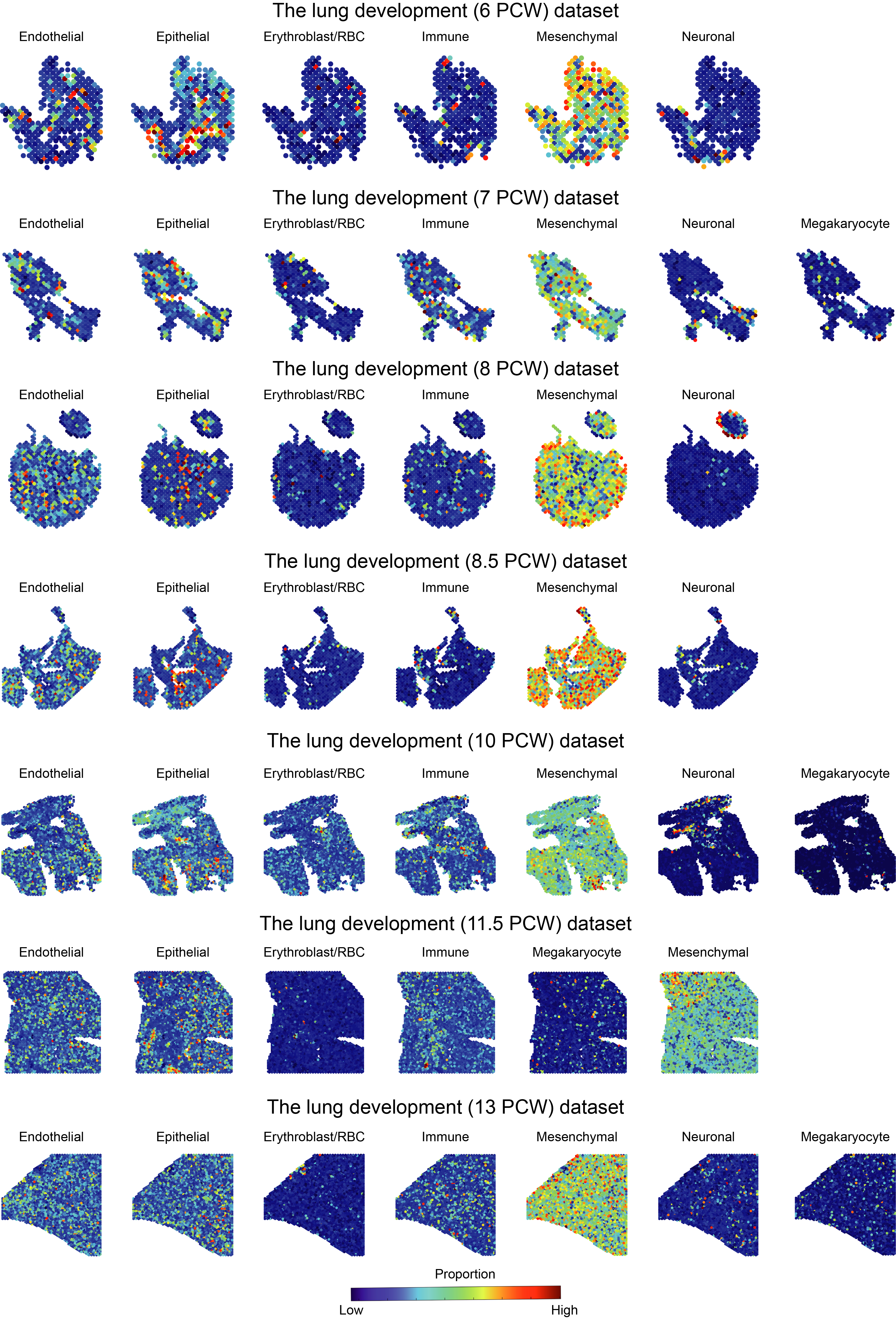


**Figure S16. Cellular deconvolution for all the cell types in the human embryonic lung.** The results of cell-type deconvolution reveal distinct spatial distribution patterns for each cell type.

## Fig. S17. Attribution analysis for all the cell types in the human embryonic lung


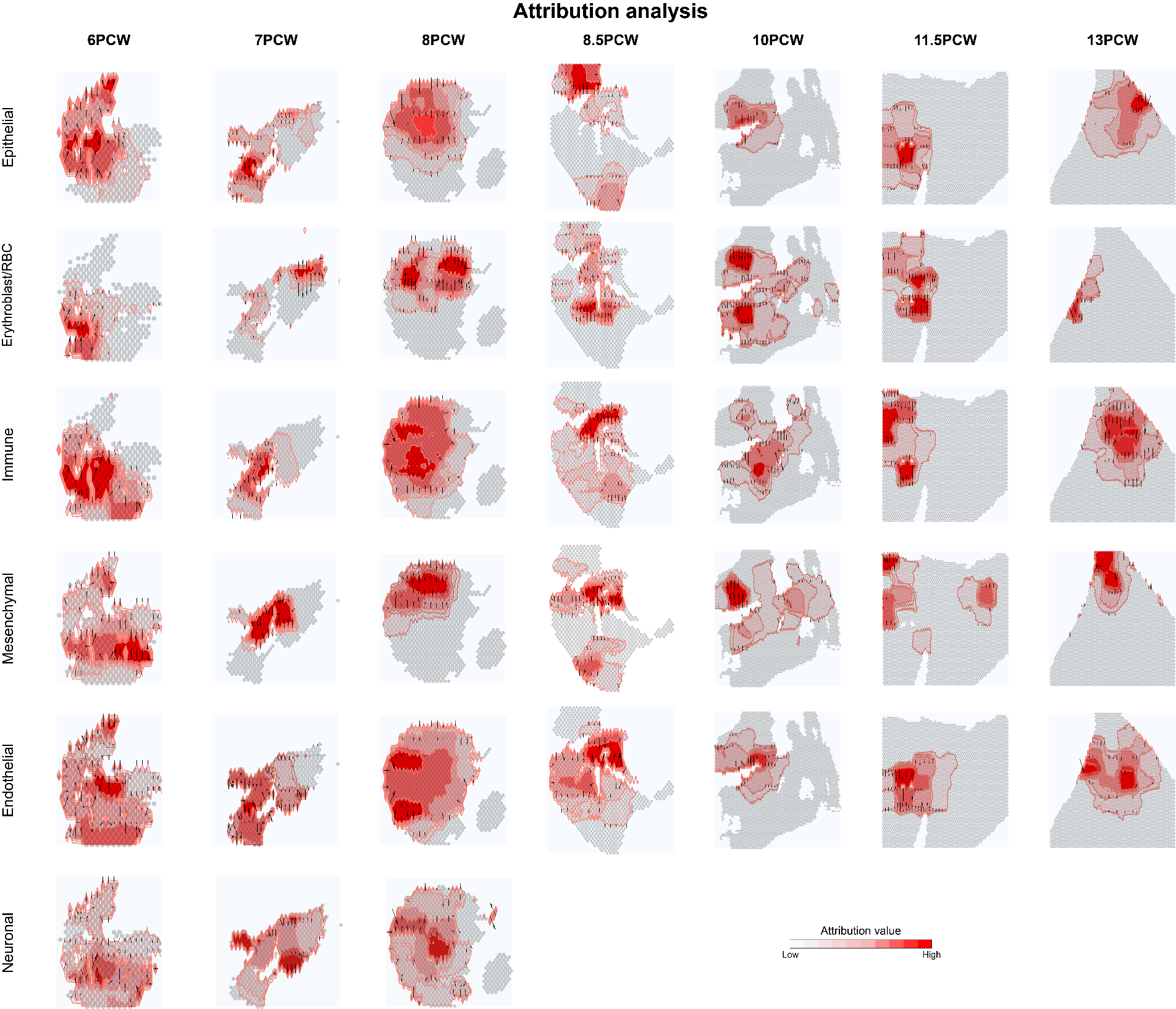


**Figure S17. Attribution analysis for all the cell types in the human embryonic lung.** The cell type-specific attribution maps with dynamic gradient field derived by eMCI for each cell type at different stages are summarized above.

## Fig. S18. Quantification for similarity between cell type-specific attribution maps in the human embryonic lung at different stages


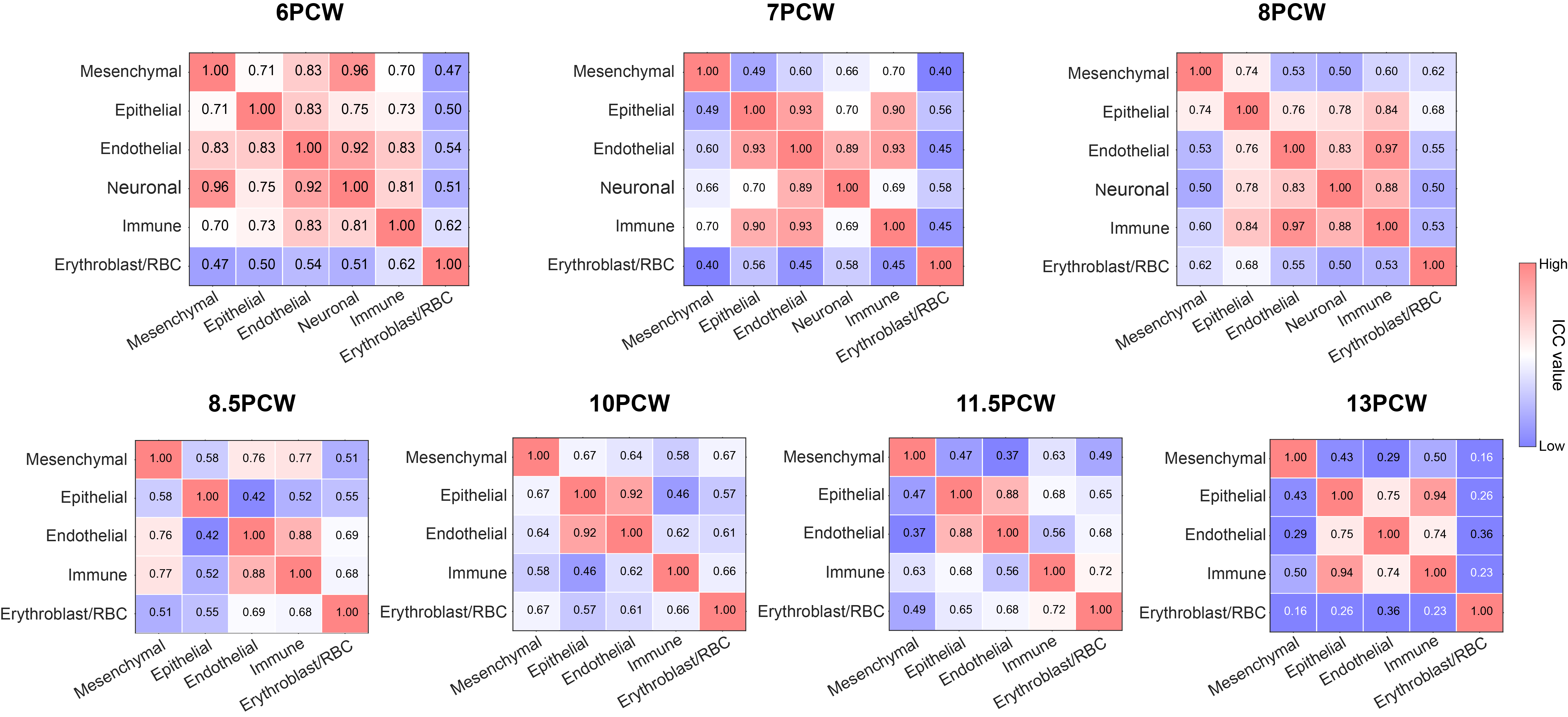


**Figure S18. Quantification for similarity between cell type-specific attribution maps in the human embryonic lung at different stages.** ICC quantifies the similarity among cell type-specific attribution maps derived by eMCI at different stages of lung development.

## Fig. S19. Correlation analysis for ICC matrices at different stages


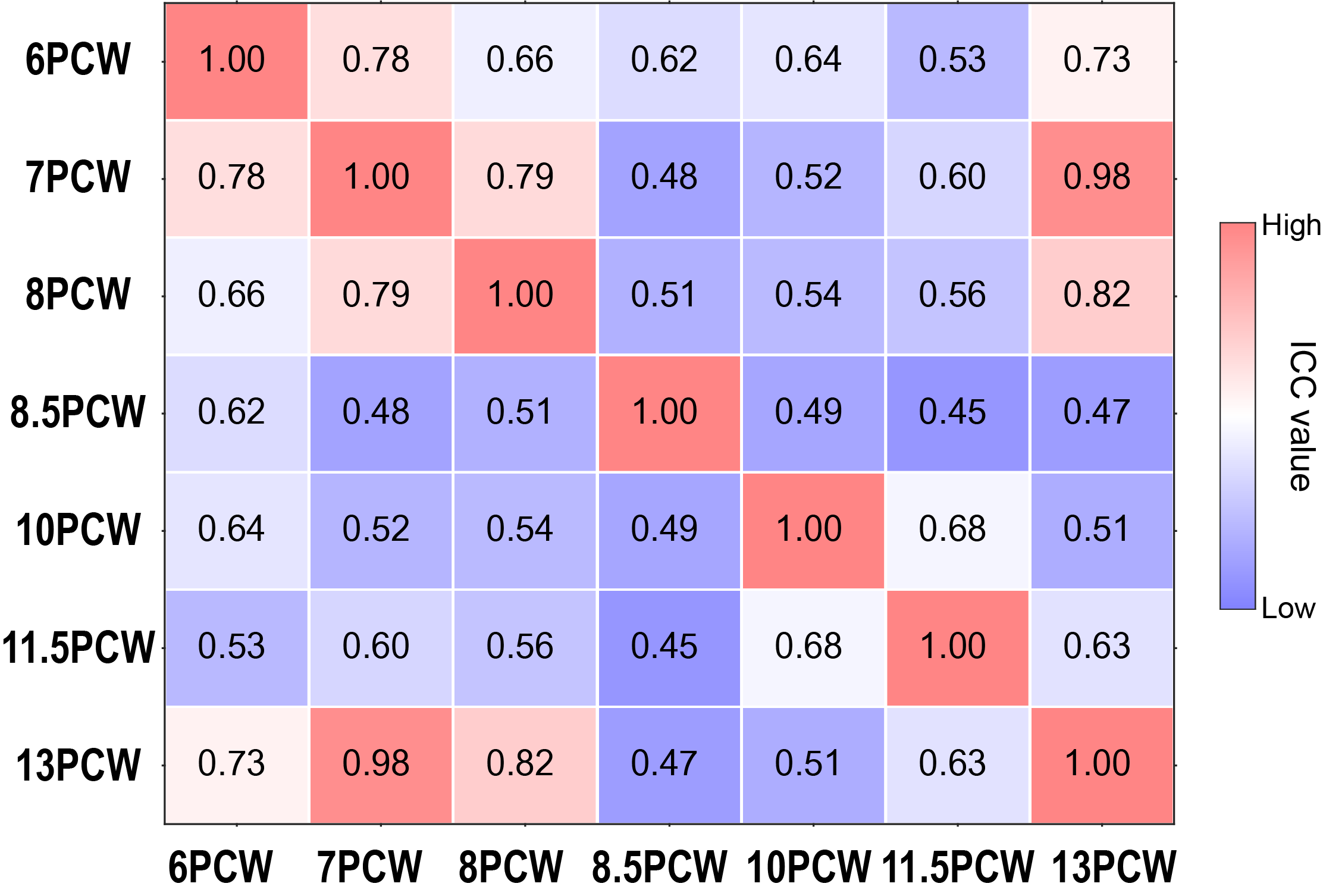


**Figure S19. Correlation analysis for ICC matrices at different stages.** ICC further quantifies the similarity among ICC matrices obtained from different stages of lung development.

## Fig. S20. Comparison of dynamic trends between cellular interaction strengths inferred by CellChat v2 and ICC values

**

**

**Figure S20. Comparison of dynamic trends between cellular interaction strengths inferred by CellChat v2 and ICC values.** The dynamic trends of the above cellular interaction strengths inferred by CellChat v2 are consistent with those of the corresponding ICC values, with positive covariances.

## Fig. S21. Comparison of eMCI performance on the raw count data and batch-corrected data


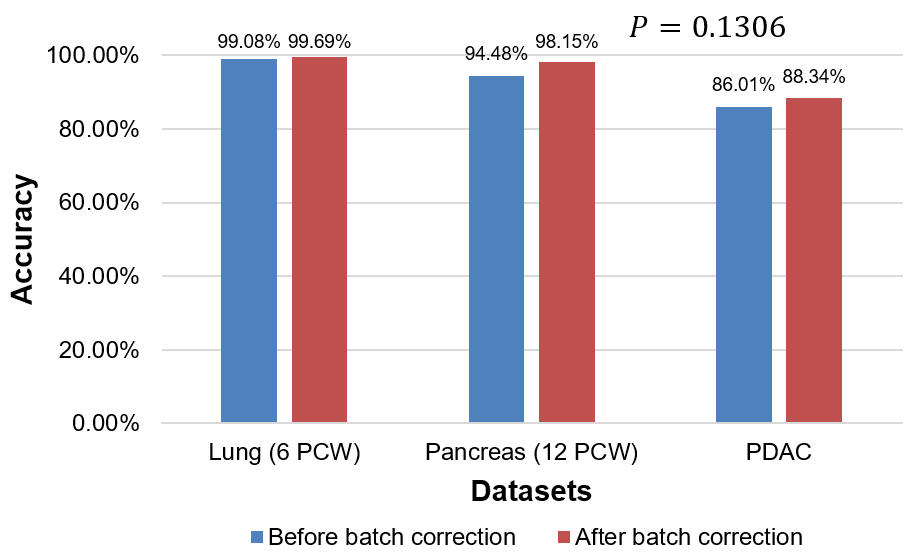


**Figure S21. Comparison of eMCI performance on the raw count data and batch-corrected data.** The results showed that batch correction effectively improved classification accuracy, though the improvement was not statistically significant ($P=0.1306$), indicating that batch effects are indeed non-negligible and should be carefully considered.

## Fig. S22. Benchmarking eMCI’s sensitivity to sequencing quality in ST and scRNA-seq datasets


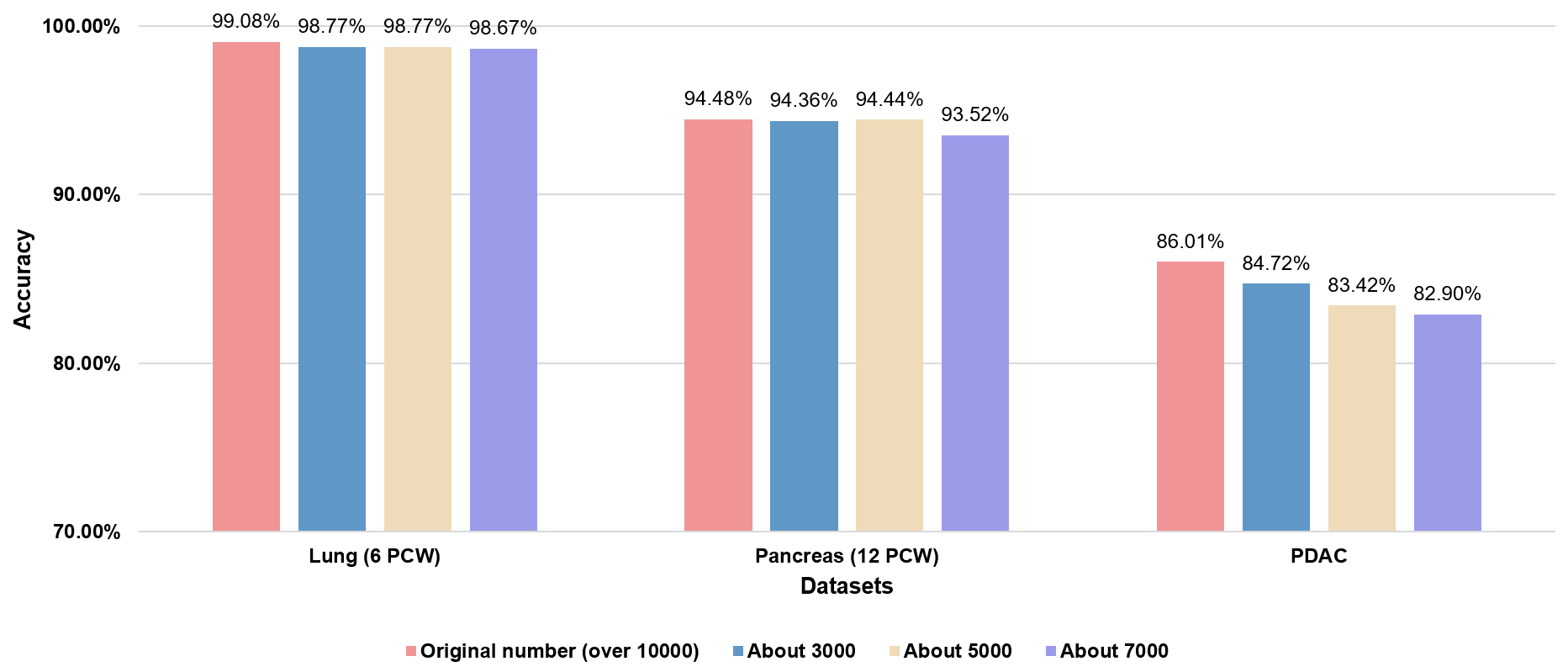


**Figure S22. Benchmarking eMCI’s sensitivity to sequencing quality in ST and scRNA-seq datasets.** The results indicated that eMCI maintains robust performance under different levels of sequencing quality. The general differences in accuracy compared to the original results are within only 1%, with the largest difference being about 3.2%.

## Fig. S23. Benchmarking eMCI’s sensitivity to sequencing quality in ST and scRNA-seq datasets


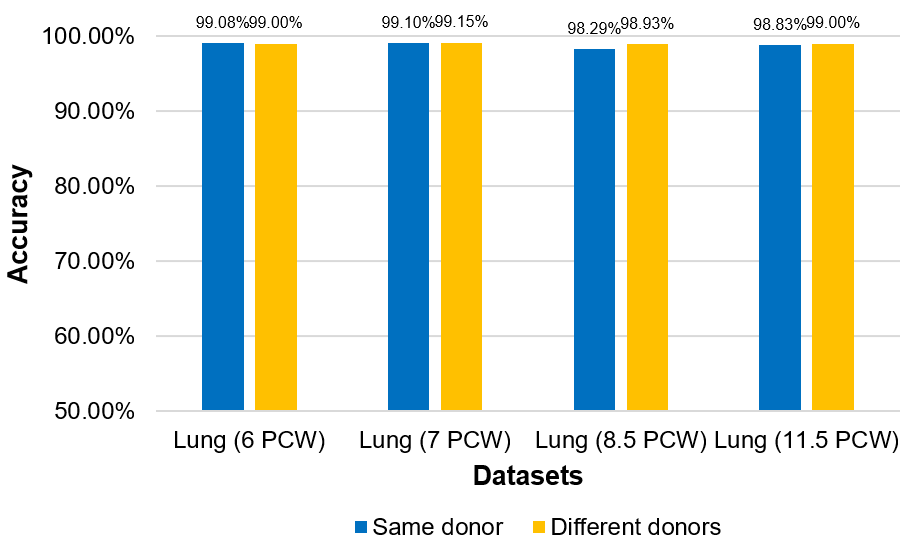


**Figure S23. Benchmarking eMCI’s sensitivity to source of ST and scRNA-seq data.** eMCI still achieved strong accuracy (with 99.00%, 99.15%, 98.93%, and 99.00% accuracy in the lung datasets from 6PCW, 7 PCW, 8.5 PCW, and 11.5 PCW, respectively) in the cell type classification task, indicating its robustness even when working with heterogeneous datasets.

## Fig. S24. Comparison of individual correlation metrics with the combination of eMCI in the cell-type deconvolution task


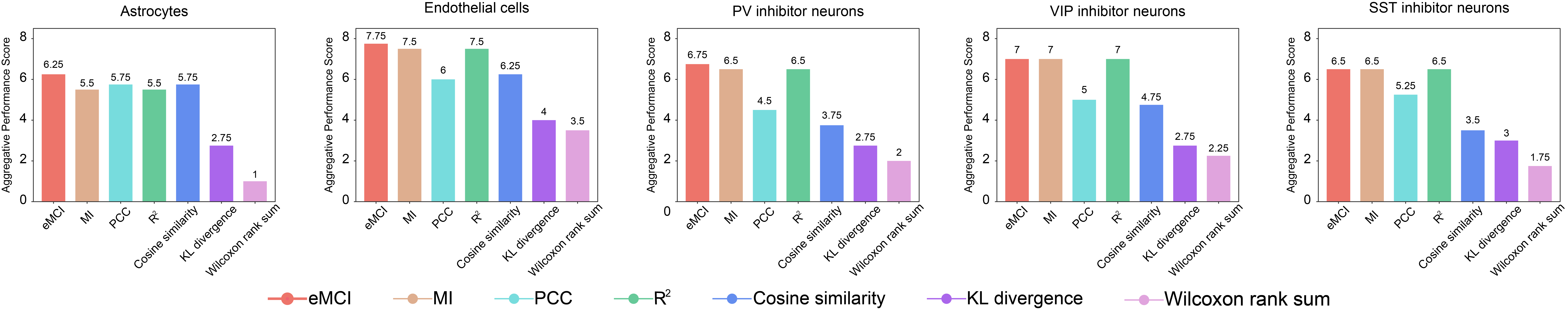


**Figure S24. Comparison of the model’s performance in the cell-type deconvolution task using multiple correlations versus individual metrics in eMCI.** The multiple correlations used in eMCI outperformed the other individual metrics in each cell type deconvolution task, validating the effectiveness and robustness of our approach.

## Fig. S25. Comparison of the model’s performance in the cell-type classification task using multiple correlations versus individual metrics in eMCI


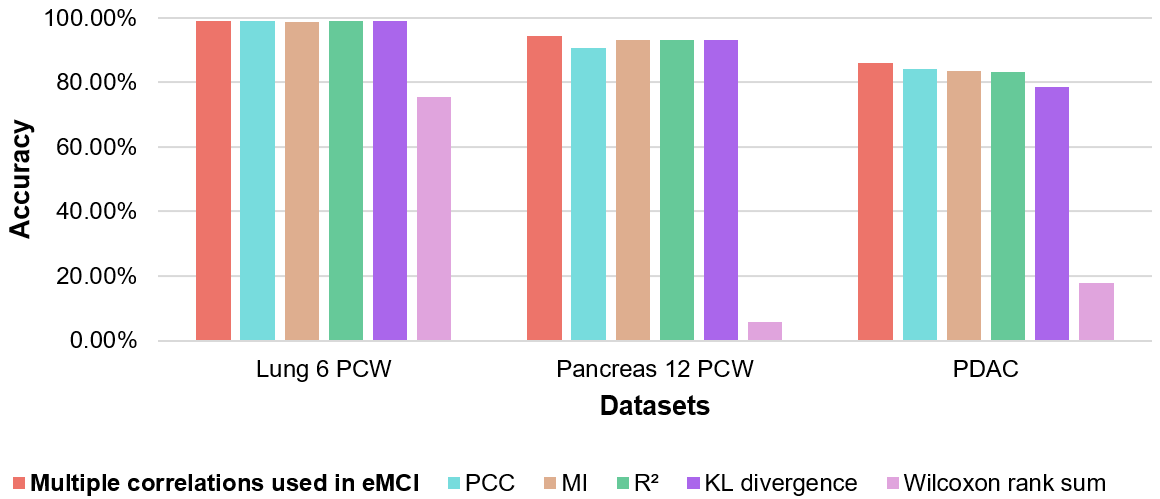


**Figure S25. Comparison of the model’s performance in the cell-type classification task using multiple correlations versus individual metrics in eMCI.** The multiple correlations used in eMCI outperformed the other individual metrics in each cell type deconvolution task, validating the effectiveness and robustness of our approach.

## Fig. S26. An illustration for the ResNet50 architecture





**Figure S26. An illustration for the ResNet50 architecture.** Overall, the adopted ResNet50 architecture includes initial convolution and pooling layers, four stages of residual blocks, a global average pooling layer, and a final fully connected layer. ResNet50 achieves deep network training by leveraging a residual learning framework and skip connections. Through these key design elements, ResNet50 effectively addresses the challenges of training very deep networks, leading to exceptional performance.

**Section S2. The supplementary tables**

## Table S1. Detailed information for the applied datasets

### Table S1.1. Detailed information for the applied single-cell RNA-seq data

| **Dataset** | **Data accession** | **Time point/sample** | **Cells in total** | **Cell-type composition** |
| --- | --- | --- | --- | --- |
| BRCA | GSE176078 |  | 100064 | B-cells  CAFs  Cancer Epithelial  Endothelial  Myeloid  Normal Epithelial  PVL  Plasmablasts  T-cells |
| Human pancreas | GSE197064 | 12 PCW | 4321 | cluster 0  cluster 1  cluster 2  cluster 3  cluster 4  cluster 5  cluster 6  cluster 7  cluster 8 |
|  |  | 20 PCW | 2122 | cluster 0  cluster 1  cluster 2  cluster 3  cluster 4  cluster 5  cluster 6  cluster 7  cluster 8  cluster 9 |
| PDAC | GSE111672 | Sample A | 1926 | Acinar cells  Cancer clone A  Cancer clone B  Ductal - APOL1 high/hypoxic  Ductal - CRISP3 high/centroacinar like  Ductal - MHC Class II  Ductal - terminal ductal like  Endocrine cells  Endothelial cells  Fibroblasts  Macrophages A  Macrophages B  Mast cells  Monocytes  RBCs  T cells & NK cells  Tuft cells  mDCs A  mDCs B  pDCs |
| Lung | GSE215895 | 6 PCW | 4884 | Endothelial cell  Epithelial cell  Erythroblast/RBC  Immune cell  Mesenchymal cell  Neuronal cell |
|  |  | 7 PCW | 11730 | Endothelial cell  Epithelial cell  Erythroblast/RBC  Immune cell  Megakaryocyte  Mesenchymal cell  Neuronal cell |
|  |  | 8 PCW | 4356 | Endothelial cell  Epithelial cell  Erythroblast/RBC  Immune cell  Mesenchymal cell  Neuronal cell |
|  |  | 8.5 PCW | 5557 | Endothelial cell  Epithelial cell  Erythroblast/RBC  Immune cell  Mesenchymal cell  Neuronal cell |
|  |  | 10 PCW | 3781 | Endothelial cell  Epithelial cell  Erythroblast/RBC  Immune cell  Megakaryocyte  Mesenchymal cell  Neuronal cell |
|  |  | 11.5 PCW | 15845 | Endothelial cell  Epithelial cell  Erythroblast/RBC  Immune cell  Megakaryocyte  Mesenchymal cell |
|  |  | 13 PCW | 6579 | Endothelial cell  Epithelial cell  Erythroblast/RBC  Immune cell  Megakaryocyte  Mesenchymal cell  Neuronal cell |
| Zebrafish | GSE159709 | Sample E | 1911 | Tumor  Macrophages  Erythrocytes  Interface (tumor)  Unknown  Neutrophils |
|  |  | Sample F | 1085 | Tumor  Macrophages  Keratinocytes  Neutrophils  Interface (tumor) |
| Plants | OMIX002290 | 12 dpi | 8229 | Epidermis  Infected cells  Inner cortex  Outer cortex  Uninfected cells  Unknown  Vascular bundle |
|  |  | 21 dpi | 12004 | Epidermis  Infected cells  Inner cortex  Outer cortex  Uninfected cells  Unknown  Vascular bundle |

### Table S1.2. Detailed information for the applied spatial transcriptomics data

| **Dataset** | **Data accession** | **Time point/sample** | **Spots in total** | **Replicate section** |
| --- | --- | --- | --- | --- |
| BRCA | 10X Genomics Website (https://support.10xgenomics.com/  spatialgene-expression/datasets) |  | 4727 |  |
| Human pancreas | GSE197317 | 12 PCW | 294 | Rep 2 |
|  |  | 20 PCW | 2126 | Rep 1 |
| PDAC | GSE111672 | Sample A | 428 |  |
| Lung | GSE215897 | 6 PCW | 355 | Rep 1 |
|  |  | 7 PCW | 386 | Rep 6 |
|  |  | 8 PCW | 894 | Rep 1 |
|  |  | 8.5 PCW | 954 | Rep 1 |
|  |  | 10 PCW | 3212 | Rep 4 |
|  |  | 11.5 PCW | 4400 | Rep 2 |
|  |  | 13 PCW | 3468 | Rep 1 |
| Zebrafish | GSE159709 | Sample A | 2179 |  |
|  |  | Sample B | 2677 |  |
|  |  | Sample C | 2425 |  |
| Plants | OMIX002290 | 12 dpi | 2229(sm1) | Rep 1 |
|  |  | 12 dpi | 1871(sm2) | Rep 2 |
|  |  | 21 dpi | 1593 (lg1) | Rep 1 |
|  |  | 21 dpi | 1981 (lg2) | Rep 2 |

## Table S2. Classification accuracy of the eMCI framework under different conditions for different datasets

| Condition  Data sets | Input with single-cell and ST data | Input with only single-cell data | Classification for cell types with single-cell and ST data as input | Classification for time with single-cell and ST data as input |
| --- | --- | --- | --- | --- |
| human embryonic lung data | 98.49% | 94.71% | 98.49% | 100.00% |
| Pancreas (12 PCW) | 94.48% | 91.09% |  |  |
| PDAC | 86.01% | 69.69% |  |  |

## Table S3. Comparison of eMCI deconvolution performance with other methods using different evaluation metrics on simulated data

| **JSD** | | | | | | | | | | | | | | | | | | | | | |
| --- | --- | --- | --- | --- | --- | --- | --- | --- | --- | --- | --- | --- | --- | --- | --- | --- | --- | --- | --- | --- | --- |
|  | | | eMCI | | | | Cell2location | | | SPOTlight | | | Seurat | | DestVI | | STRIDE | | SpaOTsc | | novoSpaRc |
| **Astro** | | | 0.4409 | | | | 0.4323 | | | 0.4486 | | | 0.5723 | | 0.5293 | | 0.4463 | | 0.7184 | | 0.7205 |
| **Endo** | | | 0.4660 | | | | 0.4559 | | | 0.4825 | | | 0.5947 | | 0.5435 | | 0.5212 | | 0.7897 | | 0.6898 |
| Excitatory L2/3 | | | 0.5019 | | | | 0.4849 | | | 0.3432 | | | 0.3570 | | 0.6118 | | 0.4736 | | 0.4868 | | 0.6077 |
| Excitatory L4 | | | 0.5392 | | | | 0.5085 | | | 0.5122 | | | 0.3870 | | 0.6537 | | 0.5151 | | 0.4192 | | 0.6665 |
| Excitatory L5 | | | 0.5840 | | | | 0.5453 | | | 0.4857 | | | 0.5997 | | 0.6375 | | 0.5336 | | 0.5211 | | 0.6743 |
| Excitatory L6 | | | 0.4783 | | | | 0.4521 | | | 0.3958 | | | 0.4009 | | 0.5306 | | 0.4376 | | 0.5468 | | 0.7808 |
| **Inhibitory Pvalb** | | | 0.5810 | | | | 0.5924 | | | 0.5855 | | | 0.4601 | | 0.6751 | | 0.5834 | | 0.6859 | | 0.7128 |
| **Inhibitory Sst** | | | 0.6228 | | | | 0.6459 | | | 0.6221 | | | 0.5043 | | 0.6770 | | 0.6295 | | 0.5902 | | 0.6798 |
| **Inhibitory Vip** | | | 0.7119 | | | | 0.7240 | | | 0.7198 | | | 0.7267 | | 0.7483 | | 0.7285 | | 0.7607 | | 0.7748 |
| Micro | | | 0.7380 | | | | 0.7359 | | | 0.7347 | | | 0.8199 | | 0.7309 | | 0.7290 | | 0.8326 | | 0.7739 |
| Olig | | | 0.4213 | | | | 0.2515 | | | 0.4270 | | | 0.3442 | | 0.5970 | | 0.4488 | | 0.6195 | | 0.7976 |
| Smc | | | 0.8175 | | | | 0.8045 | | | 0.8165 | | | NaN | | 0.8119 | | 0.8054 | | 0.6985 | | 0.7645 |
| **RMSE** | | | | | | | | | | | | | | | | | | | | | |
|  | | eMCI | | | | Cell2location | | | SPOTlight | | | Seurat | | DestVI | | STRIDE | | SpaOTsc | | novoSpaRc | |
| **Astro** | | 0.1366 | | | | 0.1221 | | | 0.1361 | | | 0.1690 | | 0.1641 | | 0.1300 | | 0.1830 | | 0.1889 | |
| **Endo** | | 0.1507 | | | | 0.1794 | | | 0.1583 | | | 0.1782 | | 0.1840 | | 0.1760 | | 0.1934 | | 0.1793 | |
| Excitatory L2/3 | | 0.2670 | | | | 0.2364 | | | 0.2751 | | | 0.1933 | | 0.3065 | | 0.2486 | | 0.2396 | | 0.2885 | |
| Excitatory L4 | | 0.1851 | | | | 0.1798 | | | 0.1806 | | | 0.1866 | | 0.2265 | | 0.1753 | | 0.1812 | | 0.2333 | |
| Excitatory L5 | | 0.1457 | | | | 0.1372 | | | 0.1389 | | | 0.1964 | | 0.1613 | | 0.1318 | | 0.3020 | | 0.3690 | |
| Excitatory L6 | | 0.2640 | | | | 0.2398 | | | 0.2485 | | | 0.2130 | | 0.2953 | | 0.2411 | | 0.3319 | | 0.3884 | |
| **Inhibitory Pvalb** | | 0.0620 | | | | 0.0606 | | | 0.0619 | | | 0.1657 | | 0.0875 | | 0.0643 | | 0.1984 | | 0.2819 | |
| **Inhibitory Sst** | | 0.0622 | | | | 0.0789 | | | 0.0630 | | | 0.1802 | | 0.0727 | | 0.0807 | | 0.2269 | | 0.1721 | |
| **Inhibitory Vip** | | 0.0731 | | | | 0.0530 | | | 0.0803 | | | 0.0805 | | 0.0587 | | 0.0927 | | 0.1747 | | 0.1626 | |
| Micro | | 0.0765 | | | | 0.0469 | | | 0.0751 | | | 0.0735 | | 0.0749 | | 0.0418 | | 0.0451 | | 0.0545 | |
| Olig | | 0.2179 | | | | 0.2245 | | | 0.1865 | | | 0.1779 | | 0.2502 | | 0.2248 | | 0.2566 | | 0.2810 | |
| Smc | | 0.1271 | | | | 0.0961 | | | 0.1086 | | | 0.0959 | | 0.1193 | | 0.0980 | | 0.0903 | | 0.1178 | |
| **PCC** | | | | | | | | | | | | | | | | | | | | | |
|  | eMCI | | | Cell2location | | | | SPOTlight | | | Seurat | | | DestVI | | | STRIDE | | SpaOTsc | | novoSpaRc |
| **Astro** | 0.6033 | | | 0.7425 | | | | 0.7477 | | | 0.5707 | | | 0.0737 | | | 0.7217 | | 0.2922 | | 0.2733 |
| **Endo** | 0.5483 | | | 0.5273 | | | | 0.4239 | | | 0.5206 | | | 0.0612 | | | 0.2022 | | 0.0203 | | 0.3625 |
| Excitatory L2/3 | 0.6609 | | | 0.7665 | | | | 0.8505 | | | 0.7708 | | | -0.0550 | | | 0.7809 | | 0.6353 | | 0.4461 |
| Excitatory L4 | 0.7938 | | | 0.8282 | | | | 0.7950 | | | 0.7639 | | | -0.0081 | | | 0.8010 | | 0.7496 | | 0.2660 |
| Excitatory L5 | 0.3174 | | | 0.6539 | | | | 0.5523 | | | 0.2972 | | | -0.0801 | | | 0.5610 | | 0.5029 | | 0.1069 |
| Excitatory L6 | 0.7186 | | | 0.8378 | | | | 0.8358 | | | 0.7522 | | | 0.2764 | | | 0.7739 | | 0.4748 | | -0.1929 |
| **Inhibitory Pvalb** | 0.6819 | | | 0.7089 | | | | 0.6273 | | | 0.6376 | | | 0.0100 | | | 0.6806 | | 0.2386 | | 0.1442 |
| **Inhibitory Sst** | 0.6135 | | | 0.4888 | | | | 0.5031 | | | 0.5902 | | | 0.0509 | | | 0.5198 | | 0.5068 | | 0.3472 |
| **Inhibitory Vip** | 0.5716 | | | 0.4003 | | | | 0.4314 | | | 0.1007 | | | 0.0178 | | | 0.3851 | | 0.1617 | | 0.1709 |
| Micro | -0.0668 | | | -0.0312 | | | | -0.0499 | | | -0.0462 | | | -0.0495 | | | -0.0107 | | -0.0382 | | 0.1885 |
| Olig | 0.8500 | | | 0.9530 | | | | 0.9116 | | | 0.8733 | | | 0.0133 | | | 0.9041 | | 0.3567 | | -0.0034 |
| Smc | -0.0872 | | | 0.1383 | | | | -0.1442 | | | NaN | | | -0.0384 | | | 0.0482 | | 0.3511 | | 0.0398 |
| **SSIM** | | | | | | | | | | | | | | | | | | | | | |
|  | eMCI | | | | Cell2location | | | SPOTlight | | | Seurat | | | DestVI | | | STRIDE | | SpaOTsc | | novoSpaRc |
| **Astro** | 0.5419 | | | | 0.4422 | | | 0.3592 | | | 0.4670 | | | 0.0474 | | | 0.3434 | | 0.0962 | | 0.1039 |
| **Endo** | 0.5072 | | | | 0.4776 | | | 0.2490 | | | 0.4286 | | | 0.0443 | | | 0.1061 | | 0.0186 | | 0.0989 |
| Excitatory L2/3 | 0.4755 | | | | 0.4864 | | | 0.8248 | | | 0.7356 | | | -0.0407 | | | 0.5584 | | 0.4422 | | 0.1802 |
| Excitatory L4 | 0.5552 | | | | 0.5520 | | | 0.4749 | | | 0.7426 | | | 0.0023 | | | 0.5015 | | 0.7433 | | 0.2165 |
| Excitatory L5 | 0.1052 | | | | 0.2558 | | | 0.5536 | | | 0.2980 | | | -0.0237 | | | 0.2714 | | 0.2669 | | 0.0559 |
| Excitatory L6 | 0.5248 | | | | 0.5741 | | | 0.6983 | | | 0.7429 | | | 0.2384 | | | 0.6307 | | 0.4444 | | -0.1636 |
| **Inhibitory Pvalb** | 0.3998 | | | | 0.3761 | | | 0.3533 | | | 0.6316 | | | 0.0130 | | | 0.3038 | | 0.2451 | | 0.1243 |
| **Inhibitory Sst** | 0.2669 | | | | 0.1308 | | | 0.2144 | | | 0.5831 | | | 0.0350 | | | 0.1642 | | 0.4681 | | 0.3484 |
| **Inhibitory Vip** | 0.1150 | | | | 0.0930 | | | 0.0848 | | | 0.1140 | | | 0.0093 | | | 0.0740 | | 0.1456 | | 0.1791 |
| Micro | -0.0311 | | | | -0.0016 | | | -0.0183 | | | -0.0051 | | | -0.0142 | | | 0.0097 | | -0.0016 | | 0.0263 |
| Olig | 0.7606 | | | | 0.9483 | | | 0.7542 | | | 0.8119 | | | 0.0154 | | | 0.6553 | | 0.0396 | | 0.0021 |
| Smc | -0.0052 | | | | 0.0080 | | | -0.0041 | | | NaN | | | -0.0008 | | | 0.0071 | | 0.3516 | | 0.0879 |

* The target cell types in the main text are marked in bold.

## Table S4. Matching relationship between clusters and cell type annotations in zebrafish melanomas

| Sample E | | Sample F | |
| --- | --- | --- | --- |
| Cluster | Cell type | Cluster | Cell type |
| E-cluster 0 | Tumor | F-cluster 0 | Tumor |
| E-cluster 1 | Macrophages | F-cluster 1 | Macrophages |
| E-cluster 2 | Erythrocytes | F-cluster 2 | Keratinocytes |
| E-cluster 3 | Interface (tumor) | F-cluster 3 | Neutrophils |
| E-cluster 4 | Unknown |  |  |

## Table S5. GO enrichment analysis for zebrafish melanoma based on the cell-type deconvolution by Cell2location

### Table S5.1. GO enrichment analysis for the DEGs selected from the paired dataset of samples A and F

| Enriched Gene Ontology (GO) terms | Gene Ratio | Enriched p-adjust |
| --- | --- | --- |
| DNA replication origin binding (GO:0003688) | 6/65 | 7.277E-09 |
| Pre-replicative complex assembly involved in nuclear cell cycle DNA replication (GO:0006267） | 5/71 | 3.0196E-08 |
| Pre-replicative complex assembly (GO:0036388） | 5/71 | 3.0196E-08 |
| Pre-replicative complex assembly involved in cell cycle DNA replication (GO:1902299） | 5/71 | 3.0196E-08 |
| DNA metabolic process (GO:0006259） | 15/71 | 6.5144E-08 |
| DNA replication initiation (GO:0006270） | 6/71 | 8.9448E-08 |
| Double-strand break repair via break-induced replication (GO:0000727） | 5/71 | 1.5233E-07 |
| DNA replication (GO:0006260） | 9/71 | 2.8121E-07 |
| Cellular response to DNA damage stimulus (GO:0006974） | 13/71 | 1.1155E-06 |
| Nuclear DNA replication (GO:0033260） | 5/71 | 1.1974E-06 |
| Cell cycle DNA replication (GO:0044786） | 5/71 | 1.8192E-06 |
| DNA repair (GO:0006281） | 11/71 | 3.8106E-06 |
| ATPase activity (GO:0016887） | 12/65 | 4.358E-06 |
| Single-stranded DNA binding (GO:0003697） | 6/65 | 7.7697E-06 |
| MCM complex (GO:0042555） | 4/74 | 1.5672E-05 |
| Helicase activity (GO:0004386） | 7/65 | 2.3486E-05 |
| DNA-dependent DNA replication (GO:0006261） | 6/71 | 6.6323E-05 |
| Cell cycle DNA replication initiation (GO:1902292） | 3/71 | 6.7907E-05 |
| Nuclear cell cycle DNA replication initiation (GO:1902315） | 3/71 | 6.7907E-05 |
| Mitotic DNA replication initiation (GO:1902975） | 3/71 | 6.7907E-05 |

### Table S5.2. GO enrichment analysis for the DEGs selected from the paired dataset of samples B and E

| Enriched Gene Ontology (GO) terms | Gene Ratio | Enriched p-adjust |
| --- | --- | --- |
| Chromosome segregation (GO:0007059) | 24/61 | 7.06602E-31 |
| Mitotic cell cycle process (GO:1903047) | 28/61 | 7.06602E-31 |
| Cell division (GO:0051301) | 25/61 | 2.91321E-27 |
| Nuclear chromosome segregation (GO:0098813) | 20/61 | 1.26814E-26 |
| Nuclear division (GO:0000280) | 21/61 | 3.95959E-25 |
| Mitotic nuclear division (GO:0140014) | 19/61 | 1.68169E-24 |
| Organelle fission (GO:0048285) | 21/61 | 1.14245E-23 |
| Sister chromatid segregation (GO:0000819) | 17/61 | 1.22884E-23 |
| Mitotic sister chromatid segregation (GO:0000070) | 16/61 | 3.0193E-23 |
| Spindle (GO:0005819) | 15/59 | 2.89539E-17 |
| Regulation of cell cycle (GO:0051726) | 20/61 | 1.50486E-15 |
| Chromosome, centromeric region (GO:0000775) | 12/59 | 1.89744E-15 |
| Chromosomal region (GO:0098687) | 12/59 | 2.37458E-14 |
| Microtubule cytoskeleton organization involved in mitosis (GO:1902850) | 11/61 | 1.22592E-13 |
| Mitotic spindle organization (GO:0007052) | 9/61 | 1.4815E-11 |
| Tubulin binding (GO:0015631) | 13/52 | 3.05114E-11 |
| Microtubule binding (GO:0008017) | 12/52 | 4.45608E-11 |
| Regulation of cell cycle process (GO:0010564) | 12/61 | 4.57729E-11 |
| Condensed chromosome (GO:0000793) | 9/59 | 1.01381E-10 |
| Microtubule (GO:0005874) | 12/59 | 1.40525E-10 |

## Table S6. Quantification of the overlap between the attribution distribution and the spatial distribution for each cell type

## Table S6.1. The number of intersections between high-attribution spots and high-proportion spots for each cell type

| Replicate sections | Number of high-attribution/high-proportion spots | Epidermis | Infected cell | Inner cortex | Outer cortex | Uninfected cell | Unknown | Vascular bundle |
| --- | --- | --- | --- | --- | --- | --- | --- | --- |
| 12-dpi nodule1 | 445 | 50 | 11 | 121 | 33 | 118 | 104 | 89 |
| 12-dpi nodule2 | 374 | 165 | 11 | 142 | 31 | 77 | 91 | 133 |
| 21-dpi nodule1 | 318 | 79 | 156 | 100 | 104 | 82 | 102 | 31 |
| 21-dpi nodule2 | 396 | 188 | 172 | 110 | 122 | 95 | 170 | 35 |

## Table S6.2. The proportion of intersection spots within high-attribution/high-proportion spots for each cell type

| Replicate sections | Number of high-attribution/high-proportion spots | Epidermis | Infected cell | Inner cortex | Outer cortex | Uninfected cell | Unknown | Vascular bundle |
| --- | --- | --- | --- | --- | --- | --- | --- | --- |
| 12-dpi nodule1 | 445 | 0.1124 | 0.0247 | 0.2719 | 0.0742 | 0.2652 | 0.2337 | 0.2000 |
| 12-dpi nodule2 | 374 | 0.4412 | 0.0294 | 0.3797 | 0.0829 | 0.2059 | 0.2433 | 0.3556 |
| 21-dpi nodule1 | 318 | 0.2484 | 0.4906 | 0.3145 | 0.3270 | 0.2579 | 0.3208 | 0.0975 |
| 21-dpi nodule2 | 396 | 0.4747 | 0.4343 | 0.2778 | 0.3081 | 0.2399 | 0.4293 | 0.0884 |

## Table S7. The error between the communication strengths inferred by different metrics and that of PlantPhoneDB

| **Cellular interactions**  **Metrics** | **Outer cortex-Uninfected cells**  **(12 dpi)** | **Inner cortex-Uninfected cells**  **(12 dpi)** | **Inner cortex-Uninfected cells**  **(21 dpi)** | **Infected cells-Epidermis**  **(12 dpi)** | **Unknown-Uninfected cells**  **(12 dpi)** | **Infected cells-Outer cortex**  **(12 dpi)** |
| --- | --- | --- | --- | --- | --- | --- |
| Cosine similarity | 0.0445 | 0.1253 | 0.0575 | 0.1686 | 0.2053 | 0.2101 |
| MI | 0.2226 | 0.3476 | 0.3024 | 0.3163 | 0.2816 | 0.2783 |
| PCC | 0.0411 | 0.1259 | 0.0472 | 0.1476 | 0.1878 | 0.1898 |
| ICC | 0.0457 | 0.0301 | 0.0659 | 0 | 0.0371 | 0.0406 |

## Table S8. Scaled cellular interaction strength by CellChat v2

| **Table S8.1 Scaled cellular interaction strength at 6 PCW** | | | | | |
| --- | --- | --- | --- | --- | --- |
|  | endothelial | epithelial | erythroblast/RBC | immune | mesenchymal |
| endothelial | 1.0000 | 0.5924 | 0.1218 | 0.2717 | 0.3424 |
| epithelial | 0.4368 | 0.5299 | 0.0096 | 0.1957 | 0.4034 |
| erythroblast/RBC | 0.1462 | 0.2613 | 0.0000 | 0.0703 | 0.0183 |
| immune | 0.3149 | 0.1698 | 0.1009 | 0.1213 | 0.0999 |
| mesenchymal | 0.4452 | 0.7621 | 0.0874 | 0.2691 | 0.7466 |

| **Table S8.2 Scaled cellular interaction strength at 7 PCW** | | | | | |
| --- | --- | --- | --- | --- | --- |
|  | endothelial | epithelial | erythroblast/RBC | immune | mesenchymal |
| endothelial | 0.6809 | 0.5168 | 0.1657 | 0.2337 | 0.4610 |
| epithelial | 0.5125 | 0.5151 | 0.1281 | 0.2280 | 0.4479 |
| erythroblast/RBC | 0.2950 | 0.1968 | 0.0200 | 0.0392 | 0.0018 |
| immune | 0.2402 | 0.1930 | 0.1479 | 0.1856 | 0.2137 |
| mesenchymal | 0.7843 | 0.7068 | 0.0823 | 0.2433 | 1.0000 |

| **Table S8.3 Scaled cellular interaction strength at 8 PCW** | | | | | |
| --- | --- | --- | --- | --- | --- |
|  | endothelial | epithelial | erythroblast/RBC | immune | mesenchymal |
| endothelial | 1.0000 | 0.6669 | 0.2846 | 0.3929 | 0.3334 |
| epithelial | 0.5055 | 0.5734 | 0.1735 | 0.3540 | 0.1832 |
| erythroblast/RBC | 0.3572 | 0.5541 | 0.0046 | 0.2533 | 0.0293 |
| immune | 0.4388 | 0.3439 | 0.2548 | 0.3577 | 0.1833 |
| mesenchymal | 0.7351 | 0.8389 | 0.1995 | 0.3687 | 0.9222 |

| **Table S8.4 Scaled cellular interaction strength at 8.5 PCW** | | | | | |
| --- | --- | --- | --- | --- | --- |
|  | endothelial | epithelial | erythroblast/RBC | immune | mesenchymal |
| endothelial | 1.0000 | 0.5780 | 0.0645 | 0.3427 | 0.2617 |
| epithelial | 0.3401 | 0.4263 | 0.0102 | 0.2229 | 0.1650 |
| erythroblast/RBC | 0.1804 | 0.4533 | 0.0005 | 0.1950 | 0.0011 |
| immune | 0.2560 | 0.2132 | 0.0036 | 0.1258 | 0.1180 |
| mesenchymal | 0.4646 | 0.6562 | 0.0000 | 0.3061 | 0.4371 |

| **Table S8.5 Scaled cellular interaction strength at 10 PCW** | | | | | |
| --- | --- | --- | --- | --- | --- |
|  | endothelial | epithelial | erythroblast/RBC | immune | mesenchymal |
| endothelial | 0.8574 | 0.7519 | 0.3090 | 0.4777 | 0.4307 |
| epithelial | 0.4984 | 0.5302 | 0.1386 | 0.2888 | 0.2898 |
| erythroblast/RBC | 0.5682 | 0.6159 | 0.0047 | 0.4486 | 0.2530 |
| immune | 0.1501 | 0.0877 | 0.0475 | 0.1395 | 0.0515 |
| mesenchymal | 0.8494 | 1.0000 | 0.3193 | 0.6091 | 0.9233 |

| **Table S8.6 Scaled cellular interaction strength at 11.5 PCW** | | | | | |
| --- | --- | --- | --- | --- | --- |
|  | endothelial | epithelial | erythroblast/RBC | immune | mesenchymal |
| endothelial | 0.6406 | 0.7324 | 0.2559 | 0.5256 | 0.3423 |
| epithelial | 0.4284 | 0.5199 | 0.1613 | 0.3416 | 0.2222 |
| erythroblast/RBC | 0.5125 | 0.8673 | 0.0326 | 0.5722 | 0.3306 |
| immune | 0.1358 | 0.0774 | 0.0563 | 0.1415 | 0.0722 |
| mesenchymal | 0.5730 | 1.0000 | 0.1386 | 0.7239 | 0.7008 |

| **Table S8.7 Scaled cellular interaction strength at 13 PCW** | | | | | |
| --- | --- | --- | --- | --- | --- |
|  | endothelial | epithelial | erythroblast/RBC | immune | mesenchymal |
| endothelial | 0.7015 | 0.6802 | 0.1633 | 0.4837 | 0.3559 |
| epithelial | 0.3944 | 0.5199 | 0.0808 | 0.3243 | 0.2399 |
| erythroblast/RBC | 0.3783 | 0.6739 | 0.0000 | 0.4806 | 0.0603 |
| immune | 0.1081 | 0.0456 | 0.0129 | 0.1304 | 0.0274 |
| mesenchymal | 0.6359 | 1.0000 | 0.0389 | 0.6644 | 0.8093 |

## Table S9. Comparison of dynamic trends between cellular interaction strengths inferred by CellChat v2 and ICC values

| **Cellular interaction** | **Quantitative index** | **6 PCW** | **7 PCW** | **8 PCW** | **8.5 PCW** | **10 PCW** | **11.5 PCW** | **13 PCW** | **Covariance** |
| --- | --- | --- | --- | --- | --- | --- | --- | --- | --- |
| mesenchymal-erythroblast | Scaled interaction strength | 0.11 | 0.08 | 0.23 | 0.00 | 0.57 | 0.47 | 0.10 | 0.0191 |
|  | ICC | 0.47 | 0.4 | 0.62 | 0.51 | 0.67 | 0.49 | 0.16 |  |
| mesenchymal-endothelial | Scaled interaction strength | 0.79 | 1.25 | 1.07 | 0.73 | 1.28 | 0.91 | 0.99 | -0.0101 |
|  | ICC | 0.83 | 0.6 | 0.53 | 0.76 | 0.64 | 0.37 | 0.29 |  |
| epithelial-endothelial | Scaled interaction strength | 1.03 | 1.03 | 1.17 | 0.92 | 1.25 | 1.16 | 1.07 | 0.0129 |
|  | ICC | 0.83 | 0.93 | 0.76 | 0.42 | 0.92 | 0.88 | 0.75 |  |
| epithelial-erythroblast | Scaled interaction strength | 0.27 | 0.32 | 0.73 | 0.46 | 0.75 | 1.03 | 0.75 | 0.00579 |
|  | ICC | 0.5 | 0.56 | 0.68 | 0.55 | 0.57 | 0.65 | 0.26 |  |
| mesenchymal-epithelial | Scaled interaction strength | 1.17 | 1.15 | 1.02 | 0.82 | 1.29 | 1.22 | 1.24 | 0.0078 |
|  | ICC | 0.71 | 0.49 | 0.074 | 0.58 | 0.67 | 0.47 | 0.43 |  |
| mesenchymal-immune | Scaled interaction strength | 0.37 | 0.46 | 0.55 | 0.42 | 0.66 | 0.8 | 0.69 | -0.0103 |
|  | ICC | 0.7 | 0.7 | 0.6 | 0.77 | 0.58 | 0.63 | 0.5 |  |
| epithelial-immune | Scaled interaction strength | 0.37 | 0.42 | 0.70 | 0.44 | 0.38 | 0.42 | 0.37 | 0.0046 |
|  | ICC | 0.73 | 0.9 | 0.84 | 0.52 | 0.46 | 0.68 | 0.94 |  |
| endothelial-immune | Scaled interaction strength | 0.59 | 0.47 | 0.83 | 0.60 | 0.63 | 0.66 | 0.59 | 0.0007 |
|  | ICC | 0.83 | 0.93 | 0.97 | 0.88 | 0.62 | 0.56 | 0.74 |  |
| endothelial-erythroblast | Scaled interaction strength | 0.27 | 0.46 | 0.64 | 0.24 | 0.88 | 0.77 | 0.54 | 0.0032 |
|  | ICC | 0.54 | 0.45 | 0.55 | 0.69 | 0.61 | 0.68 | 0.36 |  |
| immune-erythroblast | Scaled interaction strength | 0.17 | 0.19 | 0.51 | 0.20 | 0.50 | 0.63 | 0.49 | -0.0002 |
|  | ICC | 0.62 | 0.45 | 0.53 | 0.68 | 0.66 | 0.72 | 0.23 |  |
| Total cellular interactions | Scaled interaction strength |  | | | | | | | 0.0082 |
|  | ICC |  | | | | | | |  |

## Table S10. Matching information for homologous genes between soybean and Arabidopsis thaliana

| Arabidopsis thaliana | Soybean | Arabidopsis thaliana | Soybean | Arabidopsis thaliana | Soybean | Arabidopsis thaliana | Soybean |
| --- | --- | --- | --- | --- | --- | --- | --- |
| *AT1G11580* | *GLYMA-01G240300* | *AT5G60640* | *GLYMA-12G172800* | *AT2G23130* | *GLYMA-05G102100* | *AT5G66210* | *GLYMA-16G128600* |
| *AT1G11590* | *GLYMA-01G240300* | *AT4G03210* | *GLYMA-12G101800* | *AT4G37450* | *GLYMA-05G102100* | *AT3G03530* | *GLYMA-16G081200* |
| *AT4G03930* | *GLYMA-01G240300* | *AT5G60640* | *GLYMA-13G326200* | *AT1G55350* | *GLYMA-05G167200* | *AT5G46630* | *GLYMA-17G155100* |
| *AT2G45220* | *GLYMA-01G137700* | *AT3G53230* | *GLYMA-13G323600* | *AT5G46630* | *GLYMA-05G111900* | *AT4G19640* | *GLYMA-18G045000* |
| *AT3G62250* | *GLYMA-01G029200* | *AT5G03340* | *GLYMA-13G323600* | *AT4G20140* | *GLYMA-05G134800* | *AT5G13000* | *GLYMA-18G300200* |
| *AT5G19730* | *GLYMA-01G006800* | *AT4G03210* | *GLYMA-13G304400* | *AT5G44700* | *GLYMA-05G134800* | *AT5G48380* | *GLYMA-18G246400* |
| *AT5G56000* | *GLYMA-02G302500* | *AT2G16385* | *GLYMA-13G201100* | *AT5G09350* | *GLYMA-05G191200* | *AT3G51550* | *GLYMA-18G215800* |
| *AT5G56010* | *GLYMA-02G302500* | *AT3G53230* | *GLYMA-13G143600* | *AT4G25230* | *GLYMA-06G316600* | *AT1G53910* | *GLYMA-19G262700* |
| *AT5G56030* | *GLYMA-02G302500* | *AT5G03340* | *GLYMA-13G143600* | *AT5G51450* | *GLYMA-06G316600* | *AT3G09840* | *GLYMA-19G183400* |
| *AT3G02885* | *GLYMA-02G245600* | *AT4G24190* | *GLYMA-14G219700* | *AT3G25610* | *GLYMA-06G196400* | *AT3G63080* | *GLYMA-19G154100* |
| *AT2G43790* | *GLYMA-02G138800* | *AT2G26300* | *GLYMA-14G098000* | *AT5G25260* | *GLYMA-06G065600* | *AT3G22400* | *GLYMA-20G144600* |
| *AT5G06730* | *GLYMA-02G171600* | *AT5G56000* | *GLYMA-14G011600* | *AT5G09350* | *GLYMA-06G152800* | *AT5G26340* | *GLYMA-20G144300* |
| *AT3G62250* | *GLYMA-02G036000* | *AT5G56010* | *GLYMA-14G011600* | *AT4G04340* | *GLYMA-07G263300* | *AT3G21630* | *GLYMA-20G054500* |
| *AT2G35860* | *GLYMA-03G204300* | *AT5G56030* | *GLYMA-14G011600* | *AT1G53910* | *GLYMA-07G044300* | *AT3G54840* | *GLYMA-20G182400* |
| *AT3G52370* | *GLYMA-03G204300* | *AT2G30860* | *GLYMA-14G031000* | *AT1G17420* | *GLYMA-07G039900* | *AT3G54920* | *GLYMA-20G112600* |
| *AT3G53230* | *GLYMA-03G182800* | *AT2G30870* | *GLYMA-14G031000* | *AT1G72520* | *GLYMA-07G039900* | *AT5G26340* | *GLYMA-20G144100* |
| *AT5G03340* | *GLYMA-03G182800* | *AT1G78580* | *GLYMA-15G210100* | *AT5G13000* | *GLYMA-08G361500* | *AT3G54820* | *GLYMA-20G179700* |
| *AT2G45220* | *GLYMA-03G029400* | *AT3G62250* | *GLYMA-15G129800* | *AT5G55480* | *GLYMA-08G324500* | *AT2G28950* | *GLYMA-02G248500* |
| *AT5G53470* | *GLYMA-04G233600* | *AT2G13820* | *GLYMA-15G052200* | *AT3G25610* | *GLYMA-08G268900* | *AT4G04460* | *GLYMA-07G262600* |
| *AT5G49680* | *GLYMA-04G096600* | *AT2G06850* | *GLYMA-16G045000* | *AT3G15730* | *GLYMA-08G211700* | *AT1G01560* | *GLYMA-07G066800* |
| *AT5G42740* | *GLYMA-04G032600* | *AT2G46070* | *GLYMA-16G032900* | *AT1G55350* | *GLYMA-08G125500* | *AT4G01370* | *GLYMA-07G066800* |
| *AT2G25110* | *GLYMA-04G061900* | *AT1G53830* | *GLYMA-16G014100* | *AT5G09350* | *GLYMA-08G156400* | *AT3G13750* | *GLYMA-08G193500* |
| *AT1G35140* | *GLYMA-04G100400* | *AT3G14310* | *GLYMA-16G014100* | *AT5G36870* | *GLYMA-08G157400* | *AT4G04460* | *GLYMA-09G005600* |
| *AT4G08950* | *GLYMA-04G100400* | *AT2G06850* | *GLYMA-16G150300* | *AT1G67560* | *GLYMA-08G102900* | *AT2G19500* | *GLYMA-09G063900* |
| *AT1G47710* | *GLYMA-04G036200* | *AT5G38910* | *GLYMA-16G060800* | *AT4G20140* | *GLYMA-08G090000* | *AT3G13750* | *GLYMA-13G350700* |
| *AT2G13820* | *GLYMA-05G119100* | *AT5G38910* | *GLYMA-16G060700* | *AT5G44700* | *GLYMA-08G090000* | *AT3G12490* | *GLYMA-13G189500* |
| *AT2G31980* | *GLYMA-05G149800* | *AT4G24190* | *GLYMA-17G258700* | *AT3G51550* | *GLYMA-09G273300* | *AT4G05050* | *GLYMA-13G176100* |
| *AT2G18170* | *GLYMA-05G157200* | *AT2G26300* | *GLYMA-17G226700* | *AT5G48380* | *GLYMA-09G246600* | *AT4G05050* | *GLYMA-13G138600* |
| *AT4G36450* | *GLYMA-05G157200* | *AT5G67400* | *GLYMA-17G163200* | *AT1G32090* | *GLYMA-09G211000* | *AT4G02890* | *GLYMA-13G117900* |
| *AT5G67400* | *GLYMA-05G103600* | *AT4G12420* | *GLYMA-17G138300* | *AT1G25390* | *GLYMA-10G271200* | *AT4G05320* | *GLYMA-13G117700* |
| *AT4G12420* | *GLYMA-05G056100* | *AT5G56000* | *GLYMA-18G074100* | *AT5G26340* | *GLYMA-10G249500* | *AT2G19500* | *GLYMA-13G104700* |
| *AT4G03210* | *GLYMA-06G302300* | *AT5G56010* | *GLYMA-18G074100* | *AT3G54820* | *GLYMA-10G211000* | *AT2G28950* | *GLYMA-14G068000* |
| *AT1G35140* | *GLYMA-06G102100* | *AT5G56030* | *GLYMA-18G074100* | *AT3G54840* | *GLYMA-10G208300* | *AT3G12490* | *GLYMA-15G227500* |
| *AT4G08950* | *GLYMA-06G102100* | *AT2G30860* | *GLYMA-18G111200* | *AT3G09840* | *GLYMA-10G057100* | *AT3G13750* | *GLYMA-15G023800* |
| *AT5G53470* | *GLYMA-06G131200* | *AT2G30870* | *GLYMA-18G111200* | *AT3G46060* | *GLYMA-11G142300* | *AT1G01560* | *GLYMA-16G032900* |
| *AT5G42740* | *GLYMA-06G032500* | *AT4G39640* | *GLYMA-18G021100* | *AT4G34460* | *GLYMA-11G118500* | *AT4G01370* | *GLYMA-16G032900* |
| *AT2G25110* | *GLYMA-06G062900* | *AT4G39650* | *GLYMA-18G021100* | *AT1G17420* | *GLYMA-11G197500* | *AT2G19500* | *GLYMA-17G054500* |
| *AT1G47710* | *GLYMA-06G036300* | *AT5G48485* | *GLYMA-18G152400* | *AT1G72520* | *GLYMA-11G197500* | *AT4G04460* | *GLYMA-17G011500* |
| *AT5G49680* | *GLYMA-06G098300* | *AT2G35860* | *GLYMA-19G201600* | *AT5G66210* | *GLYMA-11G077300* | *AT5G39020* | *GLYMA-19G069300* |
| *AT2G43790* | *GLYMA-07G206200* | *AT3G52370* | *GLYMA-19G201600* | *AT4G35790* | *GLYMA-11G081500* | *AT5G39030* | *GLYMA-19G069300* |
| *AT5G12370* | *GLYMA-07G046100* | *AT3G53230* | *GLYMA-19G183400* | *AT5G05170* | *GLYMA-12G237000* | *AT4G02890* | *GLYMA-20G141600* |
| *AT5G19730* | *GLYMA-07G125100* | *AT5G03340* | *GLYMA-19G183400* | *AT4G27270* | *GLYMA-12G216900* | *AT2G40410* | *GLYMA-02G260600* |
| *AT2G46070* | *GLYMA-07G066800* | *AT3G01420* | *GLYMA-19G011700* | *AT4G34460* | *GLYMA-12G043900* | *AT3G56170* | *GLYMA-02G260600* |
| *AT2G06925* | *GLYMA-07G129900* | *AT5G38910* | *GLYMA-20G220900* | *AT3G46060* | *GLYMA-12G065600* | *AT3G11820* | *GLYMA-02G195400* |
| *AT5G48485* | *GLYMA-08G344300* | *AT2G01270* | *GLYMA-20G124700* | *AT3G09840* | *GLYMA-12G177100* | *AT3G53610* | *GLYMA-02G094100* |
| *AT5G56000* | *GLYMA-08G332900* | *AT4G33420* | *GLYMA-20G214200* | *AT3G15730* | *GLYMA-13G364900* | *AT2G23450* | *GLYMA-04G035100* |
| *AT5G56010* | *GLYMA-08G332900* | *AT1G76850* | *GLYMA-20G174900* | *AT1G54280* | *GLYMA-13G348200* | *AT5G09870* | *GLYMA-05G187300* |
| *AT5G56030* | *GLYMA-08G332900* | *AT5G24090* | *GLYMA-20G164600* | *AT3G13900* | *GLYMA-13G348200* | *AT1G27320* | *GLYMA-05G148100* |
| *AT2G30860* | *GLYMA-08G306800* | *AT5G21100* | *GLYMA-20G051900* | *AT3G46060* | *GLYMA-13G333500* | *AT1G11260* | *GLYMA-07G189500* |
| *AT2G30870* | *GLYMA-08G306800* | *AT5G46630* | *GLYMA-01G199000* | *AT3G09840* | *GLYMA-13G323600* | *AT1G27320* | *GLYMA-08G105000* |
| *AT5G19730* | *GLYMA-08G033000* | *AT5G66210* | *GLYMA-01G166100* | *AT4G27270* | *GLYMA-13G284200* | *AT1G11260* | *GLYMA-08G059700* |
| *AT2G13820* | *GLYMA-08G074100* | *AT4G35790* | *GLYMA-01G162100* | *AT5G01410* | *GLYMA-13G225000* | *AT5G09870* | *GLYMA-08G145600* |
| *AT2G18170* | *GLYMA-08G115200* | *AT1G32090* | *GLYMA-01G010100* | *AT5G05170* | *GLYMA-13G202500* | *AT3G53610* | *GLYMA-10G290000* |
| *AT4G36450* | *GLYMA-08G115200* | *AT2G38460* | *GLYMA-01G128300* | *AT4G19640* | *GLYMA-13G153000* | *AT3G11820* | *GLYMA-10G082400* |
| *AT5G56000* | *GLYMA-08G032900* | *AT5G03570* | *GLYMA-01G128300* | *AT3G09840* | *GLYMA-13G143600* | *AT1G02680* | *GLYMA-10G001200* |
| *AT5G56010* | *GLYMA-08G032900* | *AT3G25610* | *GLYMA-01G092900* | *AT5G12110* | *GLYMA-13G073200* | *AT1G02680* | *GLYMA-10G014500* |
| *AT5G56030* | *GLYMA-08G032900* | *AT2G04780* | *GLYMA-02G307700* | *AT3G02880* | *GLYMA-14G214700* | *AT1G47830* | *GLYMA-17G257900* |
| *AT2G06925* | *GLYMA-08G028800* | *AT5G55480* | *GLYMA-02G296400* | *AT2G04780* | *GLYMA-14G005300* | *AT3G53610* | *GLYMA-18G287600* |
| *AT4G26140* | *GLYMA-09G062900* | *AT5G12110* | *GLYMA-02G276600* | *AT5G12110* | *GLYMA-14G039100* | *AT3G53610* | *GLYMA-20G099300* |
| *AT5G56870* | *GLYMA-09G062900* | *AT5G21170* | *GLYMA-02G275500* | *AT5G21170* | *GLYMA-14G040700* | *AT5G49360* | *GLYMA-09G038600* |
| *AT2G01270* | *GLYMA-10G265900* | *AT2G27080* | *GLYMA-02G274400* | *AT2G27080* | *GLYMA-14G041700* | *AT2G15230* | *GLYMA-10G262800* |
| *AT1G76850* | *GLYMA-10G217200* | *AT5G66210* | *GLYMA-02G048300* | *AT5G05170* | *GLYMA-15G275000* | *AT5G39180* | *GLYMA-16G060800* |
| *AT3G53230* | *GLYMA-10G057100* | *AT1G53910* | *GLYMA-03G263700* | *AT5G36870* | *GLYMA-15G268800* | *AT5G39180* | *GLYMA-16G060700* |
| *AT5G03340* | *GLYMA-10G057100* | *AT3G09840* | *GLYMA-03G182800* | *AT1G54280* | *GLYMA-15G025800* | *AT5G39180* | *GLYMA-20G220900* |
| *AT5G67400* | *GLYMA-11G049600* | *AT3G63080* | *GLYMA-03G151500* | *AT3G13900* | *GLYMA-15G025800* | *AT2G15230* | *GLYMA-20G127800* |
| *AT1G78580* | *GLYMA-12G234200* | *AT3G08710* | *GLYMA-03G001800* | *AT3G46060* | *GLYMA-15G040600* | *AT2G46710* | *GLYMA-03G259800* |
| *AT3G45640* | *GLYMA-12G073000* | *AT3G60280* | *GLYMA-03G109900* | *AT5G01410* | *GLYMA-15G087200* | *AT5G64440* | *GLYMA-15G060700* |
| *AT3G53230* | *GLYMA-12G177100* | *AT5G09350* | *GLYMA-04G213400* | *AT1G53910* | *GLYMA-16G012600* |  |  |
| *AT5G03340* | *GLYMA-12G177100* | *AT4G34460* | *GLYMA-04G013100* | *AT3G54820* | *GLYMA-16G155000* |  |  |
| *AT5G60640* | *GLYMA-12G067700* | *AT3G25610* | *GLYMA-04G166100* | *AT5G48380* | *GLYMA-16G079200* |  |  |

**Section S3. The Supplementary Notes**

## Supplementary Note S1. Detailed description for the application workflow of eMCI

As for interpretability, most traditional methods rely on known ligand-receptor databases, which represent prior biological knowledge [1-3]. These methods detect the expression levels of ligands and receptors in different cell types to identify potential pairs and predict whether these pairs play a role in cell-to-cell communication, thereby effectively inferring their interactions. However, their applicability becomes limited in scenarios where well-established biological knowledge is absent. Unlike these traditional methods, eMCI could effectively characterize spatial expression patterns of cell signaling within the spatial context, even without the guidance of prior knowledge of ligands/receptor information. The detailed procedure of eMCI is provided in Fig. 1 of the main text. Note: The tissue section image in Fig. 1A is adapted from [4], used with permission from the original publisher.

Taking the case study for soybean as an example, eMCI identifies key spatial expression patterns and intercellular interactions as follows:

1. *Cell-type deconvolution based on multiple correlation metrics*. Generally, similarity measurements based on correlation coefficients between commonly examined genes in both spatial transcriptomics data and scRNA-seq data were used to reconstruct spatial gene expression or map cells in scRNA-seq data to their potential spatial origins [5-7]. To comprehensively explore the various types of relationships between the single-cell and spatial transcriptomics datasets, we chose to employ multiple correlation metrics, including Pearson correlation coefficient (PCC), mutual information (MI), and coefficient of determination ($R^{2}$). Specifically, Pearson correlation coefficient is used to measure the linear relationship between variables [6, 8, 9]. Mutual information was included to capture non-linear relationships [10]. The coefficient of determination is used to assess how well the variability of one variable can be explained by the other in a linear model [11]. These metrics are complementary in nature, with each one providing distinct insights into the relationships within the data. By transforming gene expression profiles into a multimodal spot-cell correlation spectrum and averaging the spot-cell correlation spectrums across cells belonging to the same type, we obtain a combined correlation score between this spot and this cell type, wherein a high combined correlation score reflects the similarity expression patterns between the spot and the cell type. We assume that this correlation indicates the contribution of that cell type to the mRNA expression in the spot, which can serve as an indicator of the deconvolution results.
2. *Transformation of spot-cell correlation spectrum into pseudo-image representations for image-based cell type classification*. We preserved the spatial positioning information of tissue sections and transformed the spot-cell correlation matrix into a pseudo-image representation. This approach enables CNNs, which excel in image-based methods, to extract more latent biological patterns, thereby improving the accuracy of classification in distinguishing cell types. It also lays the foundation for further analysis involving cell communication.
3. *Inference of key spatial expression patterns by LIME*. To enhance the interpretability of the eMCI model, the Local Interpretable Model-agnostic Explanations (LIME) method is employed to identify the most influential features (spatial domains in the spatial images) contributing to each cell-type prediction. These influential spatial regions identified by LIME are considered to be the areas most enriched with cell-type-specific information. By highlighting these regions, one can better understand the spatial domains that play a critical role in each cell-type prediction, offering insights into the underlying biological processes associated with these spatial patterns. For example, in the case study of soybean, based on the ligand-receptor information and cell communication results from PlantPhoneDB, it is found that the spatial expression distribution of the receptor gene *GLYMA-08G125500* in the outer cortex at 12 days post-infection (dpi) was statistically significantly correlated with the outer cortex-specific attribution map produced by eMCI, with a $P$-value of less than 0.0001 according to a hypergeometric test. This finding highlights the model’s interpretability, demonstrating its ability to identify key spatial distributions that are specifically associated with cell types, thus providing valuable insights into the spatial context of cellular interactions.
4. *Inference of intercellular interactions by ICC*. In the discussion above, we have reached the conclusion that multiple correlation metrics can comprehensively quantify the relationship between different variables. Therefore, the co-localization of cell-type-specific information in the attribution maps is analyzed through the ICC correlation metric, which include Pearson correlation coefficient, mutual information, and cosine similarity. Specifically, PCC evaluates their linear correlation [6], mutual information measures the shared information between two maps, revealing non-linear correlation [10], and cosine similarity assesses the similarity between the spatial patterns based on the angle between two variables [12]. A high ICC value between the attribution maps of two cell types indicates a strong co-localization pattern of the biological information of these cells within the overall spatial context. This suggests a higher likelihood of biological molecular interactions and information transfer between the two cell types, highlighting potential cellular communication and functional relationships. Taking the case study of soybean for an example, we identified a strong interaction between inner cortex cells and infected cells (0.91 ICC) at 12 dpi, which is validated by the network inferred from PlantPhoneDB, revealing a total of 59 ligand-receptor pairs. Furthermore, at 21 dpi in soybeans, the ICC between the attribution maps of infected and uninfected cells reached 0.76. Additionally, we found that the spatial expression characteristics of some key ligand-receptor pairs involved in communication between infected and uninfected cells were statistically significantly aligned with the spatial patterns observed in our attribution maps, further validating the co-localization and molecular exchange patterns predicted by our model.

In summary, eMCI integrated multimodal biological data into pseudo-images by preserving spatial information and utilizing multiple correlations, which allowed the CNN to extract deeper patterns and improve cell-type classification. By applying LIME for interpretability, eMCI identified key spatial domains enriched with cell-type-specific information. Furthermore, using ICC to analyze the co-localization of cell-type-specific attribution maps, eMCI inferred potential molecular communication between cells, with higher ICC values indicating stronger co-localization between the cell-type-specific information and increased likelihood of biological interactions.

# Supplementary references

[1] Browaeys R, Saelens W, Saeys Y. NicheNet: Modeling intercellular communication by linking ligands to target genes. *Nat. Methods*. 2020;**17**:159-162.

[2] Efremova M, Vento-Tormo M, Teichmann SA, Vento-Tormo R. CellPhoneDB: Inferring cell-cell communication from combined expression of multi-subunit ligand-receptor complexes. *Nat. Protoc*. 2020;**15**:1484-1506.

[3] Jin S, Guerrero-Juarez CF, Zhang L, Chang I, Ramos R, Kuan C-H, Myung P, Plikus MV, Nie Q. Inference and analysis of cell-cell communication using CellChat. *Nat. Commun*. 2021;**12**:1088.

[4] Olaniru OE, Kadolsky U, Kannambath S, Vaikkinen H, Fung K, Dhami P, Persaud SJ. Single-cell transcriptomic and spatial landscapes of the developing human pancreas. *Cell Metab*. 2023;**35**:184-199. e185.

[5] Bageritz J, Willnow P, Valentini E, Leible S, Boutros M, Teleman AA. Gene expression atlas of a developing tissue by single cell expression correlation analysis. *Nat. Methods*. 2019;**16**:750-756.

[6] Cang Z, Nie Q. Inferring spatial and signaling relationships between cells from single cell transcriptomic data. *Nat. Commun*. 2020;**11**:2084.

[7] Karaiskos N, Wahle P, Alles J, Boltengagen A, Ayoub S, Kipar C, Kocks C, Rajewsky N, Zinzen RP. The drosophila embryo at single-cell transcriptome resolution. *Science*. 2017;**358**:194-199.

[8] D’haeseleer P, Wen X, Fuhrman S, Somogyi R. *Mining the gene expression matrix: Inferring gene relationships from large scale gene expression data*. 1998. Springer.

[9] Liu Y, Na X, Yin C, Su Y, Sun S, Zhang B, Ren X, Baranwal VC. 3-D joint inversion of airborne electromagnetic and magnetic data based on local pearson correlation constraints. *IEEE Trans. Geosci. Remote Sens*. 2022;**60**:1-13.

[10] Liang K-C, Wang X. Gene regulatory network reconstruction using conditional mutual information. *EURASIP J. Bioinform Syst. Biol*. 2008;**2008**:1-14.

[11] Nakagawa S, Johnson PC, Schielzeth H. The coefficient of determination $R^{2}$ and intra-class correlation coefficient from generalized linear mixed-effects models revisited and expanded. *J. R. Soc. Interface*. 2017;**14**:20170213.

[12] Kotlerman L, Dagan I, Szpektor I, Zhitomirsky-Geffet M. Directional distributional similarity for lexical inference. *Nat. Lang. Eng.* 2010;**16**:359-389.
